# Nanostructured Conducting Polymers and Their Applications in Energy Storage Devices

**DOI:** 10.3390/polym15061450

**Published:** 2023-03-14

**Authors:** M. A. del Valle, M. A. Gacitúa, F. Hernández, M. Luengo, L. A. Hernández

**Affiliations:** 1 Laboratorio de Electroquímica de Polímeros, Pontificia Universidad Católica de Chile, Av. V. Mackenna 4860, Santiago 7820436, Chile; 2 Facultad de Ingeniería y Ciencias, Universidad Diego Portales, Ejército 441, Santiago 8370191, Chile; 3 Laboratorio de Electroquímica, Instituto de Química y Bioquímica, Facultad de Ciencias, Universidad de Valparaíso, Av. Gran Bretaña 1111, Playa Ancha, Valparaíso 2340000, Chile

**Keywords:** conducting polymer, nanostructured conducting polymer, energy storage device, fuel cell, electrochemical capacitor, battery

## Abstract

Due to the energy requirements for various human activities, and the need for a substantial change in the energy matrix, it is important to research and design new materials that allow the availability of appropriate technologies. In this sense, together with proposals that advocate a reduction in the conversion, storage, and feeding of clean energies, such as fuel cells and electrochemical capacitors energy consumption, there is an approach that is based on the development of better applications for and batteries. An alternative to commonly used inorganic materials is conducting polymers (CP). Strategies based on the formation of composite materials and nanostructures allow outstanding performances in electrochemical energy storage devices such as those mentioned. Particularly, the nanostructuring of CP stands out because, in the last two decades, there has been an important evolution in the design of various types of nanostructures, with a strong focus on their synergistic combination with other types of materials. This bibliographic compilation reviews state of the art in this area, with a special focus on how nanostructured CP would contribute to the search for new materials for the development of energy storage devices, based mainly on the morphology they present and on their versatility to be combined with other materials, which allows notable improvements in aspects such as reduction in ionic diffusion trajectories and electronic transport, optimization of spaces for ion penetration, a greater number of electrochemically active sites and better stability in charge/discharge cycles.

## 1. Introduction

The world is trapped in its first global energy crisis, not only because of the increase in population, which implies an increase in the need for energy but also because of the war and invasion of Russia in Ukraine, which until the end of 2022 managed to reverse the rapid recovery of energy markets and supplies around the world. A clear example of this is the record prices for the purchase of coal, the 100 dollars per barrel of oil, and even the purchase price of natural gas, equivalent to 250 dollars per barrel of oil in mid-2022. This global crisis not affect It only affects the industries that depend on the continuous consumption of these inputs but also directly affects the poorest households, and it is expected that by the year 2023, for the first time in history, it will increase to approximately 100 million people without access to electricity, due to the costs that this means in the family budget.

Times of crisis put governments and their way of reacting in the spotlight. At the level of formal institutions, at the COP21/CMP11 meeting—held in Paris in 2015—the governments of 195 countries signed an agreement where they committed to the Stated Policies Scenario or STEPS and Announced Pledges Scenario or APS to achieve the Zero Net Emissions by 2050 scenario (Net Zero Emissions by 2050 or NZE) and chart the path forward to achieve stabilization of the increase in global temperature by 1, 5 °C and universal access to electricity and modern energy systems by 2030. An example of this is driving annual investment in clean energy to over $2 trillion by 2030 in the STEPS scenario, which is an increase in more than 50% compared to current levels. In contrast to these investments, until 2022, the increase in global consumption of fossil fuels has continued to grow since the industrial revolution, which makes it possible to project more than 37,000 million tons of CO_2_ emissions per year by 2025, which is expected to be reduced thanks to the contribution of new, more efficient clean energy technologies, allowing emissions to drop to 32Gt by the year 2050 This would be equivalent to an 81% reduction in greenhouse gas emissions by 2050.

Together with the perspectives that propose a decrease in energy demand [1,2,3], from the point of view of technological development, many proposals aim to improve the conversion and storage of clean energy [4,5,6,7]. In the last decades, electrochemical energy storage, EES, has been widely considered a category to which **rechargeable batteries, supercapacitors, and fuel cells** belong [8].

In this field, the application of Conductive Polymers (CP) for EES has acquired important prominence in recent times [9,10]. CP has been shown to be a viable and competitive option for EES. Readers may note that within the three SEEs analyzed, although CPs have shown improvements in the devices studied, in recent years, the available information on their inclusion in batteries and fuel cells has decreased due to the fact that work with metallic nanocomposites has presented better results. However, readers will also be able to observe, within the field of supercapacitors, the synergy implied in these EES by the mixture of CP and metallic or carbonaceous materials, among others, which is still a matter of investigation, since their inclusion represents significant improvements in these devices.

Next, the supercapacitors and the evolution that they have presented, from the point of view of materials, to improve their energy storage capacity are discussed.

## 2. How Do Supercapacitors Contribute to Energy Shortages? 

Supercapacitors or electrochemical capacitors are promising devices for energy storage, which surpass conventional capacitors because they achieve excellent performances in terms of the power density they develop and the number of life cycles. In addition, they require almost no maintenance and do not experience memory effects, and from the point of view of power and energy, they bridge the gap between batteries and conventional capacitors [11,12,13]. They can be used as a complement or even as a substitute for batteries, particularly when high electrical power is required [9]. They have already been used in applications of various scales, such as microchips in portable electronic devices, electric vehicles, and electrical networks based on solar or wind energy [11]. 

However, despite the notable advances, both in the development of supercapacitors and the rest of EES devices, their use is still far from being fully used. There are still many systems and applications that are not fully decarbonized. Along with this, all the energy demands that would have to be sustained by renewable resources must be considered. In order to achieve these objectives, there is still a field that can be covered by EES technologies, according to the following considerations.

In current electrical grids, there has been some progress in incorporating renewable energy sources (RES) for electricity generation [12]. The most exploited RES is hydroelectric, photovoltaic, and wind [13]. Some less-used emerging sources correspond to the conversion of tidal energy and the conversion of biomass. Greenhouse gas emissions could decrease significantly if the energy sector were to become a contributing asset in the decarbonization process.

Some predictions indicate that energy production will double by 2050, reaching an annual output of 40,000 TW [12]. In addition, under the most ambitious scenario, according to the Deep Decarbonization Pathways Project (DDPP), considering only the electric power industry in the 16 countries that concentrate 75% of greenhouse gas emissions, there would be a decrease to a quarter of the total emissions [14]. This has been projected, assuming that 90% of electricity generation in 2050 would come from CO_2_-free technologies. The decarbonization of the electricity sector would allow the decrease from 530 gCO_2_ kWh^−1^ to about 33 gCO_2_ kWh^−1^ [14]. This would require adequate infrastructure for proper operation, but some technologies for generating decarbonized electricity, such as photovoltaic panels or wind turbines, do not allow a stable level of energy, given their sensitivity to environmental conditions [15]. To correct this intermittency, the use of EES technology in electrical networks is promising [4,16,17,18,19] mm. In this regard, an evolution of smart grids is projected in the near future [18], characterized by their decentralization, with a leading role of the Internet of Things, IoT [19], and with the possibility of reconfiguring and self-healing. 

Energy storage systems are considered a key part of modern electrical grids. Advancement in these technologies would allow better management of electrical networks based on RESs, ensuring load leveling in real time and avoiding load disconnection and intermittencies at times of higher energy demand [18,19,20,21,22,23]. However, at present, the world’s stationary energy storage is dominated by reversible hydroelectric power plants. An alternative to this would be based on the development and implementation of EES technologies, such as redox flow batteries or high-power supercapacitors, for applications in uninterruptible power supplies (UPS) or in real-time charge leveling [22]. 

In the transport sector, dependence on fossil fuels is still high, so a transition to decarbonized technologies is also required. Its contribution amounts to around 22% of the world’s total greenhouse gas emissions. Transport by wheel or by road is the largest contributor in this area and is responsible for 72% of CO_2_ emissions, according to the IPCC [23]. According to the International Organization of Automobile Manufacturers (OICA), in 2015, there were already around 1.2 billion vehicles [24]. In addition, commercial air transport, in 2017, emitted around 2% of total CO_2_ and 13% of emissions from the transport sector [25]. Thus, development in the manufacture and performance of electric vehicles could also benefit from the improved design of rechargeable batteries and high-power supercapacitors.

Likewise, the development and consumption of electronic and digital devices have grown notably during the last 30 years. Currently, there is a wide range of so-called consumer electronics (CE) devices, for example, household electronic equipment, laptops, smartphones, audiovisual equipment, etc. A classic example corresponds to the growing number of mobile phones, whose sales between 1994 and 2013 increased by 62 times, and by 2015, there were around 7.5 billion subscribed connections. The development of service robots is also growing, and—according to the International Federation of Robotics—it has been estimated that in 2017 around 6.1 million robots were sold for different services [26].

With the development of IoT and smart grids, where consumer electronics (CE) technologies and robots would increasingly be integrated and interconnected, an adequate supply of electrical energy would be essential for their correct operation [27,28]. It has been estimated that energy consumption for digital technologies, in a moderate scenario, would increase in the period 2013–2025 from 1.9% to 5.2% of the total global energy demand [29]. Again, it is in this area where supercapacitors can offer their comparative advantages due to the high energy power developed for the operation of a wide range of electronic devices, allowing them to meet their energy requirements.

As noted, in relation to energy demand and decarbonization, the challenge is still enormous. Under this panorama—as has been highlighted—some key contributions come from EES technologies. Bearing this in mind, many investigations in electrochemistry continue to contribute to this development, through the synthesis and design of new materials, with better properties. 

## 3. Conducting Polymers

In recent decades, a wide range of materials for the manufacture of electrodes has been investigated, trying to improve aspects such as gravimetric and volumetric density, power, costs, safety, and half-life.

Several of the commercially available EES systems include electrochemically active inorganic systems [30,31,32]. However, this involves problems in terms of the limitation of metallic resources, production costs, and the environmental footprint. **The manufacture of energy storage devices based on organic materials, including CP, may offer opportunities to improve EES technologies, allowing them to obtain electrodes of various configurations and with better mechanical properties** [33].

In addition to the above, many efforts in the synthesis and design of electrode materials are dedicated to the development of nanostructures, which improve aspects such as ionic transport, electrical conductivity, and specific capacitance [34]. Currently, it is possible to find a wide range of metallic, ceramic, and polymeric materials, among others, that can take various forms, for example, nanowires (nw) [35], nanotubes, nanocomposites, nested nanostructures, etc. **These nanostructured materials can be used for applications that provide a better quality of life and that allow the development of new decarbonized technologies [22,36,37]**.

Along these lines, this bibliographic compilation addresses these problems, where electrochemistry, polymer physicochemistry, and nanoscience, among other disciplines, converge in the development of nanostructured CP, which is currently being used for applications in various electronic devices. Their synthesis will be evaluated—mainly by electrochemical means—the ways in which the nanostructures have been achieved and how, in addition to being used to obtain electrochemical capacitors from renewable energies, they have been proposed for use in other electronic energy storage devices.

**CP has been widely considered for various applications** due to their adjustable electrical conductivity, controllable chemical, and electrochemical properties, low cost, low density, flexibility, corrosion resistance, easy processability, and good biocompatibility (when used for biological purposes) [9,38,39,40,41,42]. 

In this area, electrochemistry has played a fundamental role, both in the preparation and characterization of CP [43], because electrochemical techniques are suitable for the controlled synthesis of such macromolecules and for the control of a well-defined oxidation state [44]. Furthermore, electrochemical polymerization is very advantageous when the polymeric product will be used as a layer deposited on an electrode to prepare a sensor, biosensor, etc., since the control of the potential is a requirement to produce these materials with good quality. However, when electrochemical production is not possible or large amounts of polymer are required, the chemical route may also be recommended [44].

**Figure 1:**
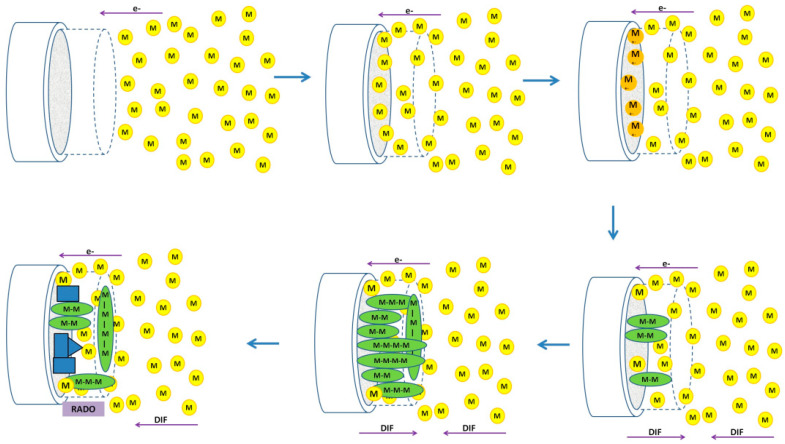
Proposed model for the electropolymerization process.

The **electropolymerization process** can be summarized in the following main stages: (a) formation of a high-density oligomeric region (HDOR) in the diffusion layer through the consecutive reactions of oxidation of the monomer, dimerization, oligomerization, and release of protons; (b) nucleation (when the interface is saturated or the oligomer becomes insoluble); (c) growth of short-chain oligomeric structures and (d) solid-state redox reactions, which expand chains and conjugation, generating a cross-linked porous matrix [39]. This has been verified by working with a rotating disk electrode and spectroscopic observations [44,45,46], in addition to the systematic analysis of the variables involved in the electrosynthesis process, such as solvent, type, and concentration of monomer, type, and concentration of support electrolyte, applied potential, etc., in the CP electrodeposition process [47,48,49,50,51,52]. These studies have made it possible to propose the electropolymerization mechanism shown in Figure 1, whose understanding allows to design and modulate the working conditions to control the morphology of the polymer that is deposited on the working electrode [53].

As noted, the main property of CP is its ability to change from a neutral state to a doped or electrically charged state, notably increasing its conductivity to a metallic regime [53,54,55]. The *p*-type doping corresponds to the partial oxidation of the polymer, and the *n*-type doping corresponds to its partial reduction. For both types of doping, the charge carriers are delocalized in the conjugate system. However, *n*-type doping is less common than *p*-type doping, since, generally, in the latter case, charge carriers are more stable [38]. In chemical polymerization processes, it is common to use suitable oxidizing agents, such as iodine [56], bromines [57], H_2_O_2_ [58], and salts of Fe^3+^ [59], Cu^2+^ [60], S_2_O_8_ [2,3,4,5,6,7,8,9,10,11,12,13,14,15,16,17,18,19,20,21,22,23,24,25,26,27,28,29,30,31,32,33,34,35,36,37,38,39,40,41,42,43,44,45,46,47,48,49,50,51,52,53,54,55,56,57,58,59,60], Cr_2_O_7_^2−^ [61], etc. In the case of electropolymerization, an external potential is applied in an electrochemical cell, promoting redox processes. To maintain electroneutrality, during the oxidation process, counter ions (also called dopants) are simultaneously incorporated into the polymeric matrix, causing a structural change due to the solvent molecules that surround the ionic species [62,63,64,65]. During the reduction process, the counter-anions and solvent molecules diffuse out of the polymeric matrix in response to external perturbation, generating a compact structure [39].

During the partial oxidation process (*p*-doping), a fraction of the polymer stabilizes a certain number of positive charges, depending on the polymer structure and the charge potential. The mole fraction of the corresponding charged monomers is the doping level. Typical values for doping levels are between 0.1 (low doping level) and 0.5 (high doping level) [39]. If the doping level exceeds a certain threshold, coulombic repulsion increases dramatically, and polymer over-oxidation occurs. Thus, the electrochemical properties, the level of charge, and the conductivity, among others, depend directly on the level of doping of the polymer.

Several theories have arisen to explain the conductivity of CP and have been tried to verify experimentally. Some refer to charge transporters based on the formation of non-local excitons, such as solitons, polarons, and bipolarons [38,66,67]. The band theory, based on Hückel’s theory of molecular orbitals, has also been used since the early 1980s to understand the known properties of CP until then [68]. Theoretical calculations suggested three absorption bands for polarons and two for bipolarons. Experimentally, this was observed in absorption spectra of various CP [68,69]. 

Regarding the model based on band theory, criticisms have arisen because it was conceived for ideal linear polymers with an infinite number of monomeric units and without defects, but some experiments have shown that electropolymerization processes produce materials with a finite number of monomers and mainly porous materials with cross-linked chains are obtained [39]. Therefore, electrical conduction cannot be linear but rather incorporates an inter-chain component. This would be better explained by a hopping process [70].

## 4. Nanostructured Materials 

Nanomaterials, compared to their counterpart, bulk materials, have attracted attention in recent decades due to their outstanding electrochemical, photonic, optical, electronic, optoelectronic, thermal, magnetic, and mechanical properties and their potential applications [30]. That is, the **construction of nanostructured materials allows new and remarkable properties**. According to their morphology, nanostructures can be generically classified into zero-dimensional (0D), one-dimensional (1D), two-dimensional (2D), and three-dimensional (3D) and, by definition, at least in one of these dimensional axes has a size smaller than 100 nm [71,72,73].

Recently, there has been a great development of one-dimensional (1D) nanostructured materials, such as nw [73], nanotubes [74], nanofibers [75], nanoribbons [76], nanorods [77], among others, with notable emergent properties [78,79,80]. Obtaining this type of nanostructure has been achieved through a wide variety of synthetic approaches. Although it is not the objective of this compilation to review each one of them, for each type of structure, it is convenient to dwell on some production and design strategies for nw due to the advantages they offer from a mechanical and electrochemical point of view. 

Nw has been manufactured using different synthetic approaches (chemical and physical), for example, hydrothermal/solvothermal synthesis, sol-gel, coprecipitation, template-assisted, laser-assisted catalytic growth, electrospinning, thermal evaporation, chemical vapor deposition. (CVD), among others [35,81]. By controlling some characteristics of the nw, such as chemical composition, morphology, diameter, length, purity, crystallinity, and surface defects, improvements in the performance of these materials can be achieved. For example, when designing electrodes with nw, it is fundamental to monitor the structural changes of active materials during electrochemical processes [35]. Thus, nw manufacturing strategies can be classified into two broad categories, namely: spontaneous growth and growth by spatial confinement. For some materials based on elements such as V, Mo, and Ti, among others, it is easier to obtain 1D morphologies through a spontaneous growth strategy [82,83,84]. On the other hand, in other materials, whether organic or inorganic, auxiliary tools such as templates and molds are used, which corresponds to a strategy based on growth by spatial confinement [85,86,87]. 

The nw synthesis strategies, depending on the environment in which they are carried out, can also be classified in wet or dry chemistry. Among the wet strategies is the electrodeposit. This is based on the growth of materials due to the energy coming from an applied electric field and involves the use of an electrode substrate. The morphology of the product depends on factors such as the concentration and temperature of the solution, type of support electrolyte, applied potential for the deposit (electrochemical perturbation) [88], etc. 

The controlled synthesis of nanostructured CP by means of chemical or electrochemical oxidation of the appropriate monomers can be carried out with or without the assistance of templates (template-based or template-free, as they are commonly known). Template-assisted synthesis strategies are recurrent when controlling the size and morphology of nanostructures [89]. This type of synthesis can be classified—according to the template—into two types: methods based on soft-template and on hard-template. Both methods have been applied for the chemical and electrochemical synthesis of a wide range of CP nanostructures, such as polythiophene, PTh [85]; poly(3,4-ethylenedioxythiophene), PEDOT [90,91]; polypyrrole, PPy [92,93]; polyaniline, PANI [61,94,95]; polyphenylene vinylene, PPV [96]; etc.

The methods based on hard-template are those conventionally used in the synthesis of nanostructured CP. Some examples are the use of anodic aluminum oxide (AAO) membranes [97,98], track-etch polycarbonate [99], zeolites [100], silica-based mesoporous molecular sieves [101,102], polyoxometalates [103], among others. This type of synthesis has allowed high control in the production of nanostructures, such as the manufacture of nw and nanotubes. In addition, the adjustment of the dimensions of the deposited nanostructures can be controlled by regulating the pore size of the template used and the experimental conditions of the deposit [89,104]. 

The soft-template-based approach is a relatively simpler manufacturing process, which also allows the controlled synthesis of CP nanostructures. This synthesis strategy includes polymerization by surfactants [92,105], inverted micro-emulsion [106], layer-by-layer self-assembly [107], etc.

Nanostructured materials stand out in terms of the ease and precision with which they can be used to make electrodes. Nw has numerous advantages from an electrochemical and electrical conductivity point of view. For example they offer a continuous path for the flow of electrons, consequently increasing the conductivity and kinetics of the electrodes; smaller diameter materials adapt better to volume changes and structural damage related to electrochemical processes; the specific surface area at the electrode|electrolyte interface is increased, facilitating the access of ions to the active sites of the electrode, increasing the usefulness of the material; they offer a high length/diameter ratio, which is useful for constructing flexible electrodes with autonomous networks and free of agglutination, in 3D configurations; allow their use as easily assembled building blocks in more complex structures, for various applications [81].

The use of nw in electrochemical energy storage applications, despite all the advantages it offers, entails a series of challenges that must be overcome when synthesizing them to maximize the use of all the benefits associated with nanostructuring. For example, they can be easily added to each other after repeated charge/discharge cycles, decreasing the electrode capacity and increasing the internal impedance; higher surface area exposure can induce interface side reactions, reducing coulombic efficiency and life cycle; the conductivity of the nw is lower than its counterpart in bulk, due to a lower mean free path of the electron, due to surface scattering; the compact density of nw is usually lower compared to micrometric particle structuring, which results in a lower volumetric energy density; the synthesis of nw of controllable shape and size is relatively complex and its production on a large scale at low cost, still needs research and development [35].

Despite these difficulties, **the advantages of nw allow their use in potential electronic applications**, such as light-emitting diodes (LEDs), lasers, field emitters, solar cells, nanogenerators, and electrodes in energy storage devices such as rechargeable batteries, supercapacitors, and fuel cells. Capacitors can be classified into three general classes: electrostatic capacitors, electrolytic capacitors, and electrochemical capacitors [108]. Those of the first type is made up of a pair of conductors separated by a dielectric material. These capacitors operate at charging times of 10^−9^ s and are the ones that store the least energy. Electrolytic capacitors contain metal plates separated by a very thin dielectric, with a high dielectric constant and an electrolyte, to achieve a high capacitance. They can store up to 10 times more energy than an electrostatic capacitor, and their response time is on the order of 10^−4^ s. Lastly, electrochemical capacitors are based on two electrodes separated by an electrolyte. The electrodes can be based on carbon materials, metal oxides, CP, or a mixture of them. They store more energy than previous capacitors, and their response time is on the order of 1 s. Due to their outstanding performance, these capacitors are also called supercapacitors or ultracapacitors.

Supercapacitors are devices that develop an electrical performance halfway between traditional capacitors and batteries. This is reflected in the power and energy densities they have: they have a lower power density than traditional capacitors but higher than batteries. On the contrary, its energy density is higher than that of traditional capacitors but lower with respect to batteries. The charged energy they store is mainly based on reactions on the surface of the electrode materials [109,110]. However, unlike batteries, ion diffusion does not occur within the electrodes. This allows a rapid supply of energy and can be a complement to rechargeable batteries. However, the type of electrode materials and structures impose restrictions on the amount of charge they can store.

In recent years, different ways have been sought to optimize the energy density supported by a supercapacitor. In this regard, there are two main types of approach: the development of nanostructured and porous electrodes [111] and, on the other hand, the manufacture of hybrid [112] and asymmetric supercapacitors [113]. The first approach improves the specific capacitance, and the second increases the total cell voltage. In particular, with the development of new nanostructured electrodes, is sought to increase the contact surface area and/or decrease the length of the ionic diffusion path [34]. Among the electrochemical parameters that have been enhanced are specific capacitance, power, energy densities, and the stability of charge/discharge cycles, among others [114]. In addition, there has been an increase in the development of materials that have remarkable flexibility [33]. Consequently, devices with outstanding electrochemical and mechanical performances have been manufactured.

## 5. CP as Supercapacitors 

**Figure 2:**
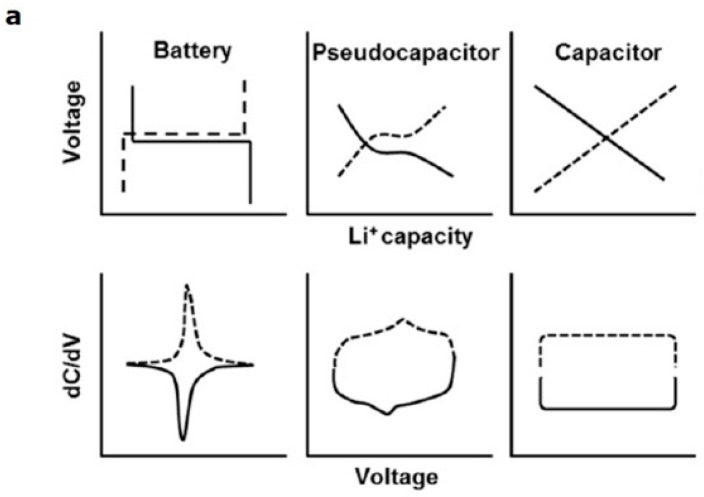

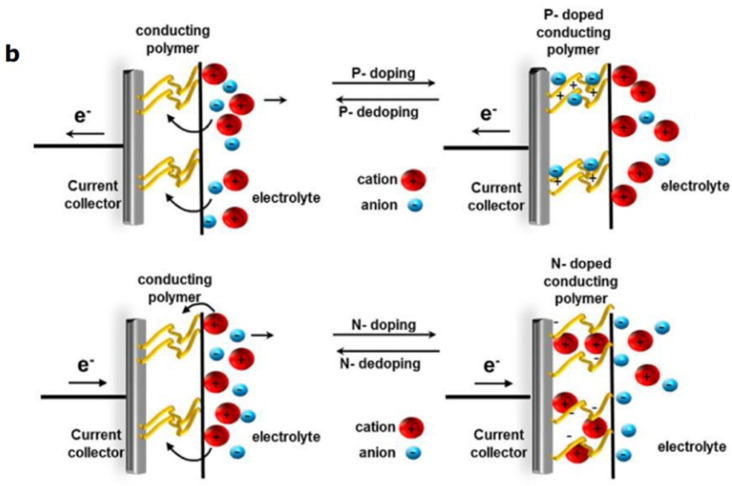
(**a**) Potential and differential capacity scheme for the three basic charge/discharge mechanisms. (**b**) charge/discharge processes for p-type and n-type doping in conducting polymers.

Supercapacitors are classified according to their charge/discharge mechanism (Figure 2a): electrochemical adsorption/desorption of cations and anions at the electrode/electrolyte interface; faradaic charge transfer reactions at the same interface and intercalation/deintercalation of cations within active materials of the electrode [109,114]. Accordingly, we speak about capacitive, pseudocapacitive, and pseudocapacitive interleaving behavior (or battery type), respectively. Consequently, supercapacitors can be classified into double-layer electrochemical capacitors (EDLCs), pseudocapacitors, and interleaving pseudocapacitors, respectively. Pseudocapacitive behaviors (with and without interleaving) close the gap between EDLCs and batteries in relation to the energy density they manage to store.

Another way to classify supercapacitors depends on the composition of the electrodes. Thus, it is possible to find symmetric, asymmetric, and hybrid supercapacitors. Symmetrical supercapacitors are made up of two identical supercapacitor-type electrodes. Generally, they are based on activated carbon (AC), reduced graphene oxide (rGO), or pseudocapacitive materials. Asymmetric supercapacitors are made up of two different supercapacitor-type electrodes [113]. One electrode is capacitive in behavior (based on carbon), and the other is made of pseudocapacitive material. This type of configuration allows the expansion of the potential operating window. Hybrid supercapacitors are composed of one electrode of the supercapacitor type and the other of the battery type [115]. The electrodes—being of a different type—operate in different potential windows.

In summary, the characteristics that define the type of electrode of a supercapacitor are based on the materials used and the possible configurations that they can adopt. In recent years, a significant number of studies can be found that report electrodes with new combinations of materials, generally nanostructured. Thus, the evaluation of the advantages and disadvantages offered by a particular material or structure allows a critical judgment to be made in the design of supercapacitors.

Due to their redox properties, CP such as PPy [116,117], polyacetylene, PA [118,119], PANI [117,120], o PEDOT [121] have been regarded as remarkable materials in supercapacitor electrodes. Both within and on their surface, these CPs can undergo redox reactions that allow for outstanding specific capacitances. Furthermore, these processes are highly reversible since transformations, such as phase changes, do not occur.

### Electronic Storage Devices

Among the mentioned polymers, both PPy and PANI have stood out, which have shown promise as materials in supercapacitor electrodes due to their low cost, environmental stability, and easy electrochemical synthesis. Both polymers can undergo *p*-doping, so it is common to use protic solvents, acids, or protic ionic liquids, for their synthesis. Unfortunately, these CPs can be damaged in the charge/discharge process. By relying on doping/undoping cycles, they suffer expansion and shrinkage in their structure (Figure 2b) [108]. This implies stress on the material and impacts the stability of its charge/discharge cycles and explains why the focus has been put on mitigating these damages and that it has not only been tried to improve its mechanical stability but also its electrical conductivity [42,108].

Another important type of electrochemical energy storage devices is batteries, an energy source made up of a system of electrochemical cells, connected in series or parallel, capable of transforming chemical energy into electrical one [122]. They are sources of power that generate a direct current in an electrical circuit.

Perhaps the main way in which batteries can be classified depends on their ability to be rechargeable or not. On the one hand, there are primary or non-rechargeable batteries, which can only be used until they are completely discharged. On the other, there are secondary or rechargeable batteries, which are useful during several charge/discharge cycles, behaving as an electrolytic cell during charging and as a galvanic cell during discharge [123].

Currently, a wide variety of rechargeable battery systems exist, in part due to the composition of the electrolytes and electrodes. Conventionally, two main types can be distinguished, namely, lead-acid [124] and alkaline batteries [125], depending on whether the electrolyte is acidic or alkaline. Within the latter are lithium-ion batteries [126], sodium ions [127], redox flow [128], nickel-cadmium, nickel-iron, nickel-zinc, nickel-metal hydride, nickel-hydrogen, silver-zinc, zinc-manganese dioxide, among others [129]. In addition to this variety related to the composition of the electrodes, the different types of electrolytes used in batteries, both liquid and solid, can be added. Beyond the conventional acidic and basic aqueous electrolytes, liquid electrolytes such as conductive molten lithium salts have been used [130], molten salts of alkali halides [129], lithium salts [131], ionic liquids [132], organic electrolytes [133], etc. On the other hand, solid electrolytes are materials that allow high ionic mobility within their crystalline structure. In this class, the lithium-ion conductors, both ceramic (inorganic) and polymeric (organic), used in lithium-ion batteries stand out [134].

In contrast to fuel cells and supercapacitors, rechargeable batteries have been predominant in terms of their use and participation in the market for energy storage and conversion devices, found in applications such as cell phones, laptops, cars, and other portable electronic devices [135]. The best performances that can be achieved with batteries that incorporate new materials allow further development and design of electronic devices of various kinds and increasingly massive use in domestic electric power generating systems.

Despite the variety of batteries that are used and sold, the real satisfaction of current energy requirements has not been achieved, demanded mainly by portable electronic devices, electric vehicles, and energy storage networks for domestic use. In addition, there is a trend towards the development of lighter, more flexible high-performance batteries [136], environmentally friendly, and less toxic [137]. In order to progress in this area, it is essential to develop materials with new functionalities, and understanding their physical-chemical properties and structure-property relationships. **One of the approaches consists of the research and development of better electrodes and battery electrolytes, totally or partially organic** [138,139].

Compared to inorganic rechargeable battery electrode materials, the highly conjugated and electrochemically active polymeric structures allow good electronic conductivity, excellent energy storage, and fast reversible redox processes. However, the use of pure conventional CP and without further morphological optimization would not allow for obtaining performance comparable to that of commercial rechargeable batteries [140]. The level of doping required for good performance in rechargeable battery electrodes could be achieved by modified structures.

It has been suggested that the formation of nanostructures, composites with inorganic materials, and the synthesis of new conjugated polymers would be promising strategies for the advancement of batteries, partially or totally organic, whose charge storage is based on reversible electrochemical reactions in amino and thioether groups, ether, carbonyl, among others [140]. In this regard, some cases can be mentioned: new types of heterocyclic conjugated polymers with ladder-type structures, have shown good performance in electrodes, or conjugated systems based on organic carbonyl compounds, which undergo *n*-type doping, have been shown as potential materials for anodes [141]. A high specific capacity of up to 230 mAh g^−1^ has been achieved for NIBP doped with Li^+^ (*n*-type doping), which means an improvement in energy storage compared to *p*-type doping with larger anions [142]. This type of research is a stimulus to explore in greater depth this class of materials.

The third type of electrochemical energy storage device is fuel cells, which store and convert chemical energy into an electrical one, for which there is a continuous supply of fuel such as hydrogen, natural gas, or methanol and an oxidizing substance, such as oxygen, air or hydrogen peroxide [122]. It can be aided by systems that provide reactants or batteries that allow its operation. Similar to other electrochemical systems, these cells are made up of an anode where oxidation occurs, a cathode where reduction occurs, and an electrolyte that allows the transport of ions between the electrodes. However, their operation differs from that of batteries and supercapacitors due to the need for a continuous supply of oxidizing substances and fuel by an external system [143]. Fuel cells are not electrically recharged, but they do require recharging of the fuel used. Directly, these cells operate due to the oxidation of hydrogen gas as fuel. Other types of substances, such as methane or methanol, in order to be used as fuels, are previously converted to hydrogen [143]. The energy density they can store is even higher than that of batteries. This allows fuel cells to be established as primary sources of energy conversion, applicable in remote systems that require a continuous supply of electricity to generate and distribute electrical power. 

Fuel cells can be classified into biological (BFCs) [144], alkaline (AFCs) [6], polymer electrolyte membrane (PEMFCs) [145], direct methanol (DMFCs) [146], direct-ethanol (DEFCs) [147], phosphoric acid (PAFCs) [148], molten carbonate (MCFCs) [149] and solid oxide (SOFCs) [150]. In this order of presentation, fuel cells operate from low to high temperatures. For example, an EFC cell can operate at 35 °C, and on the other hand, a SOFC type can operate at almost 1000 °C.

In recent decades, the most extensively researched fuel cells are the DMFC and SOFC type but above all, the PEMFC. Some of the advantages of this last type of cell are high gravimetric and volumetric power densities, fast behavioral responses due to the relatively low operating temperatures, and the possibility of using reformed gas mixtures from high energy density fuels, as well as pure hydrogen.

Cells of the BFC type are distinguished from the other more conventional classes, using a component of biological origin. They are somewhat more versatile in terms of the type of fuel since they are not limited to the use of hydrogen or methanol, allowing the generation of electrical energy from a wide range of organic substrates [151]. This class of cells can, in turn, be distinguished between those that use catalysts extracted from cells (enzymes or even mitochondria) and those that use living cells. According to these criteria, there are enzymatic fuel cells (EFCs) and microbial fuel cells (MFCs), respectively [144]. These bioelectrochemical systems have received notorious attention in recent decades because they allow the conversion of biomass into electrical energy and the treatment of wastewater. From the metabolism of organic substrates, a flow of electrons can be generated that can be diverted to an external circuit and used for various applications.

For a few years, efforts have been made to achieve CP-based, high-performance fuel cell materials. A notable example has been a cobalt-PPy nanocomposite, which has shown a good ability to reduce oxygen as well as high durability at low cost [152]. Systems based on PEDOT have been tested for oxygen reduction, showing as a good candidate for use as an electrocatalyst, free of precious metals [153]. From pyrolytic treatments of PANI, a carbon electrocatalyst doped with nitrogen and iron has been obtained, in a direct way and with good performance, which encourages similar procedures, to obtain thin layers of nanostructured material from CP [154]. In an MFC-type cell, an a-Fe_2_O_3_/PANI nanocomposite has been used, capable of electrochemically reducing Cr (VI) ions, which allows the generation of electricity, together with the remediation of wastewater [155]. These are just some examples of potential applications that CP can have in different structures for the optimization of fuel cells.

## 6. Nanostructuring CP 

Considering the antecedents summarized so far, in this compilation, a bibliographic search is carried out that allows us to know the current use of nanostructured CP, to produce new modified materials, which are used in the development of the electrochemical energy storage devices. A detailed comparison is made of the real impact of the use of materials of organic origin vs. its inorganic counterpart, with experimental data that have the report of figures of merit, comparing synthesis methods, nanostructuring methods, reproducibility, storage capacity, etc. Thus, it is possible to deliver, in short, a projection of new CP that has not been tested in this area, and that may mean a concrete advance in the search for new materials for energy storage devices.

In order to show the evolution and variety of configurations in the design of CP nanostructures, this first section provides a general review of the enormous number of publications in this field. The focus has been put on a morphological perspective of rationally designed polymeric nanostructures and how this can have an impact on the performance sought for energy storage devices, sensors, remediators, and electrocatalysts, among others. For this, various types of synthesis of polymeric nanostructures are reviewed, mainly based on PANI, PPy, PTh derivatives, co-polymers, polymer mixtures, or COFs that, in some cases, have been synergistically combined with other materials, forming hybrids. Thus, an overview of the various designs that have been achieved can be had, alternatively using oxidative chemical polymerization, electrosynthesis, CVD, pyrolysis, and hydrothermal methods, with or without the assistance of templates, to name just a few.

### 6.1. PANI 

Since the early 2000s, it has been possible to find some reports on the nanostructuring of PANI, which opened new possibilities in the optimization and application of this material. Qiu et al. [156] reported one of the first chemically synthesized PANI nanotubes, without the use of templates, by oxidative chemical polymerization, using ammonium persulfate as an oxidizing agent, in the presence of PAMAM4.0 [naphthyl(SO_3_H)_2_]_24_ or C_60_(OSO_3_H)_6_ as proton dopants, which also had an important impact on the diameters of the nanotubes achieved. By SEM micrographs, PANI nanotubes 100–300 nm in diameter and several microns long were observed. Although this constituted an advance in the way of nanostructuring PANI, development was still lacking regarding a variety of structures, reproducibility, types of reagents used, and potential applications.

A small demonstration of how some properties can be optimized from PANI nanostructuring can be seen in Huang’s et al. work [157], where the obtaining of PANI nanofibers was reported, by means of an interfacial polymerization chemical pathway, in an aqueous/organic biphasic system, synthesizing PANI nanofibers, with diameters of 30–50 nm. Both synthesis and purification did not require template removal steps, observing uniform nanofibers via TEM, forming interconnected networks. An important contribution of this study was that doping with HCl and NH_3_ gases demonstrated a greater reduction in electrical resistance (greater conductivity) compared to a conventional NIBP film in bulk, even though the thickness of the former was greater [95]. This would be explained by greater ease in the diffusion of gases through the spaces between nanofibers.

In addition to the benefits that can be achieved with PANI nanostructuring, various investigations have tested the possibility of forming nanocomposites with carbonaceous materials. For example, studies by Wang et al. [157] reported the preparation of PANI in the form of pointed nanofibers or whiskers, with a high degree of order on mesoporous carbon, obtaining a nanostructured composite. One of the main contributions made by the researchers was the obtaining of V-shaped channels with an average diameter of 3–4 nm, which facilitated the diffusion of electrochemically active materials. Another more current case is the preparation of graphene/PANI nanocomposites by in situ polymerization, reported by Zhang et al. [158]. By using ammonium persulfate as an oxidant, a chemical polymerization of aniline was carried out, forming PANI nanofibers of approximately 50 nm in diameter, uniformly distributed between graphene oxide sheets. Subsequently, the material was reduced with hydrazine to obtain the final nanocomposite. One of the optimized properties to highlight was the better electrical conductivity of the nanocomposite, compared to pristine PANI fibers.

Likewise, in the field of nanostructures based on PANI/carbonaceous material, the contributions made by some studies have been important, which have proposed their synthesis by electrochemical means. Depending on the particularities with which an electrosynthesis is carried out, this can mitigate or eliminate factors such as the use of toxic reagents, the complexity of the preparation steps, and the contamination of the product and its environment. In a Feng et al. investigation [159], the electrochemical synthesis, in one stage and on a large scale, of a nanocomposite based on graphene/PANI was developed. This was conducted from exfoliated graphene oxide—which can be reduced by a cathodic potential—and from aniline monomers. When applying an anodic potential, they polymerize, forming PANI. A series of graphene nanosheets covered with thin polymer films were obtained. The images obtained by TEM corroborate the obtaining of uniform films of the G/PANI nanocomposite, with thicknesses of 20–30 nm, reporting high electrical conductivities and surface area, as well as good biocompatibility. Consequently, due to its enhanced properties, this type of design is optimal for energy storage applications, such as supercapacitor electrodes.

The new demands, which imply obtaining more flexible and portable materials, because of scientific and technological progress, have forced scientists to work on the nanostructuring of composites that include PANI, incorporating the design of deformable materials as the main objective. The in situ electropolymerization strategy has proven to be beneficial in the preparation of this type of nanocomposite. A clear example of this was reported by Cong et al. [160], who designed a paper-type nanocomposite based on G/PANI. Without the requirement of binders, PANI nanorods were obtained on graphene nano-sheets by means of aniline electrosynthesis at 0.8 V vs. Ag|AgCl. The scalability in obtaining graphene paper was corroborated by producing a prototype of up to 22·16 cm^2^. Through images obtained by SEM, it was verified that the nanorods were not only electrodeposited on the external surface of the paper but also between graphene sheets. In this way, a material with better mechanical and electrochemical properties was achieved through a relatively simple method. 

Another approach with which flexible materials can be obtained, based on PANI/ carbonaceous material hybrids, is the formation of hydrogels or aerogels. Zeng et al. [161] obtained a hydrogel nanocomposite based on carbon nanotubes and PANI (CNT/PANI hydrogel). A dense CNT film was subjected to electrochemical activation by cyclic voltammetry (CV), managing to expand the interior of the structure due to the formation of hydrogen bubbles. Subsequently, PANI was electropolymerized, forming homogeneously distributed nanofilms around the surface of the nanotubes. Thus, a fairly porous nanocomposite was achieved, with interconnected networks of optimal electrical conductivity, which presented good mechanical performance when subjected to mechanical stress tests such as curvature, stretching, or twisting of the electrosynthesized material.

The combination with carbonaceous materials is not the only benefits one since notable cases of nanostructured hybrid materials based on PANI and metal oxides can also be found. Among these, the nanoarchitecture stands out, where PANI acts as a covering on a metallic oxide, providing not only better electrical and electrochemical properties but also mechanical ones. In a study by Lu et al. [162], the design of a three-dimensional (3D) nanostructure composed of a-Fe_2_O_3_ nw electrodeposited on a carbon fiber, which was subjected to annealing in air and covered by a PANI nanofilm, also electrosynthesized, was reported. The diameters of the a-Fe_2_O_3_ nw and the PANI nanofilm were ~20.3 nm and ~8.3 nm, respectively. The final nanocomposite, 3D a-Fe_2_O_3_@PANI, was shown to have a highly ordered structure with optimized surface area and excellent mechanical performance. 

Under a similar paradigm of NIBP-based nanocomposites, Zhou et al. [163] designed core-shell and yolk-shell type nanostructures, where a chemically synthesized NIBP film acted as a layer on a core of sulfur nanoparticles. The yolk-shell type structure differed from the other by incorporating an empty space between the core and the shell. Thanks to this, the nanostructure allowed to better accommodate the volumetric expansion of the sulfur nanoparticles during lithiation processes, compared to the core-shell type. The yolk-shell type nanostructure was achieved by heat treatment of the core-shell type nanostructure, where a vulcanization reaction and partial evaporation of elemental sulfur occurred. In addition, the porous structure of the network formed by the PANI shell was verified, whose thickness reached approximately 15 nm. This nanoarchitecture was tested as an electrode material for a lithium-sulfur (Li-S) battery, showing better performance in terms of specific capacity and cycle stability compared to a cathode based on the core-shell type structure.

Also, in two-dimensional (2D) morphologies—usually designed for battery electrodes—it is possible to find these binary materials based on NIBP/metal oxide. In this sense, it’s interesting to the work of Huang et al. [164], where the design of MnO_2_ nanosheets interspersed with PANI is shown. The nanocomposite is prepared with a simplified, one-step method using an organic/inorganic interface reaction. The oxidative polymerization of aniline was carried out with KMnO_4_. This strategy facilitated layer-by-layer assembly. During the process, there was continual diffusion of aniline from the organic phase and of the MnO_4_^−^ ion from the inorganic phase, simultaneously forming the PANI and MnO_2_ layers after the reaction at the interface. The images obtained by HRTEM revealed a porous sponge-like structure with MnO_2_ nanosheets interspersed with PANI, up to 10 nm thick. By subjecting the nanocomposite to 400 °C, the pore size increased, but the expansion did not cause material degradation, confirming the good mechanical stability. For this, PANI nanofibers sandwiched between MnO_2_ nanosheets were fundamental. The main objective was to achieve a mechanically reinforced and nanostructured material since MnO_2_ nanosheets without PANI were more easily degraded when undergoing Zn^2+^ and H^+^ ions insertion/disinsertion processes.

### 6.2. PPy 

The nanostructures and composites formed by PPy are quite numerous, and there are studies that reflect not only this but also the conditions under which it is possible to synthesize them. In this sense, Zhang et al. [165] showed the controlled chemical synthesis of various PPy nanostructures using cationic, anionic, and non-ionic surfactants and with the oxidizing agent ammonium persulfate (APS) and FeCl_3_. Because of the formation of laminar mesostructures, self-assembly of the cations of long-chain surfactants such as CTAB or DTAB, with the anions of APS, PPy nw, and nanoribbons was obtained. Whereas, with short-chain cationic surfactants (OTAB) or non-ionic, using any oxidizing agent, it was possible to synthesize PPy nanospheres. On the other hand, with anionic surfactants, it was not possible to obtain nanostructures. Through these studies, it was found that the morphology of the PPy nanostructures depended on the concentration of the pyrrole monomer and the concentration and length of the surfactant chains. In this way, control of the desired nanostructure could be achieved. However, nanostructures with a low level of doping were obtained, which could be explained by the low concentrations of monomer and oxidant and the low oxidant/monomer molar ratio. The contribution of this study lies in the demonstration of the great versatility of PPy, by demonstrating the possibility of varying and controlling its chemical synthesis in the form of nanostructures.

The formation of nanostructures based on PPy and some carbonaceous material, taking advantage of the synergy that occurs between them, can already be found at the beginning of the previous decade. Jurewicz et al. [166] synthesized a nanocomposite consisting of MWCNTs (multi-walled carbon nanotube) covered by homogeneous PPy nanolayers. Nanotubes with internal and external diameters of 2–6 nm and 10–25 nm, respectively, formed an interconnected three-dimensional mesoporous network. On this matrix, PPy was deposited both in chemical and electrochemical form, and, when comparing, it was observed that with the second method, the nanolayers had a homogeneous morphology with a thickness of ~5 nm. The incorporation of the PPy nanolayer not only improved the electrical conductivity but also, by testing the performance of the nanocomposite as an electrode in a supercapacitor, it developed a greater energy storage capacity compared to MWCNTs in the pristine form [166].

Along the same lines, new approaches and designs were developed to optimize electrical and mechanical properties. This can be seen in the work of Biswas and Drzal [167], where the design of a three-dimensional multilayer nanoarchitecture composed of graphene nanofilms and PPy nw was demonstrated. By oxidative chemical polymerization, using ammonium persulfate as the oxidant and CTAB as the surfactant, a network of 40–60 nm diameter PPy nw was obtained, where aligned monolayers of graphene nanofilms were intercalated. The integration of these materials, through this direct self-assembly approach, was produced by the large van der Waals attractive forces between the basal planes of graphene and the conjugated chains of the polymer. The G/PPy nanocomposite was investigated as an electrode in a supercapacitor. Because of the homogeneous dispersion of graphene, optimized surface area, greater ease for ion transport, high electrical conductivity, and the best mechanical stability due to the interaction between graphene and PPy, better electrochemical performances were obtained (165 F g^−1^ after 1000 cycles at 1 A g^−1^), compared to nw directly obtained in a current collector, without the presence of graphene [167].

A somewhat different case but framed in new approaches related to carbonaceous materials and PPy consists of obtaining carbons doped with nitrogen from PPy as precursor material. For example, Sevilla et al. [168] reported the design of a carbonaceous material with high porosity and high nitrogen content, using PPy as a precursor. The synthesis of the N-doped porous carbon was carried out in a single chemical activation step with KOH. Depending on the temperature at which PPy was activated (600–800 °C) and the ratio between the amounts of KOH and PPy used, products of different morphology were obtained. For a KOH:PPy ratio of 2:1 and at 600 °C, considered mild conditions, micropores of ~1 nm and 10.1% nitrogen by mass were achieved. The product thus obtained, with a three-dimensional nanostructured morphology, proved to be a good CO_2_ storage, thanks to its abundant porosity, high surface area, and significant nitrogen content, which acted as active adsorption sites [168].

Some studies have considered the combination of PPy and sulfur with different morphologies, in an increasingly common way, potentially usable in cathodes of lithium-sulfur (Li-S) batteries. In these cases, PPy does not play a role in the contribution of active redox sites but rather increases the electrical conductivity and mechanical stability of the material. Regarding one-dimensional nanostructures, Sun et al. [169] showed the synthesis of a composite based on PPy-sulfur nw networks (PPy-S). The PPy nw were synthesized chemically, using ammonium persulfate as the oxidant and CTAB as the surfactant template, reaching a diameter of ~50 nm. Then, the PPy nw network was mixed with sublimated S and subjected to high temperatures to allow S diffusion in the network. With a view to its use as a cathode in a lithium-sulfur battery, the performance of the PPy-S nanocomposite was investigated. In addition to the better electrical conductivity, the PPy nw network allowed the absorption and protection of S, which can be dissolved in an electrolyte in the form of polysulfides. The latter decreases the energy storage capacity and the life cycles of the device. The porous morphology of the PPy nw network allowed a good diffusion of ions within the cathode. In general, better mechanical and electrochemical stability of the PPy-S nanocomposite was obtained with respect to pristine S [169].

As with other polymers, the combination of PPy and S in core-shell-type structures is remarkable. For example, Li et al. [170] reported the design of a core-shell type nanostructure made up of sulfur-covering PPy nanofibers deposited on a PPy film. The PPy nanofibers were synthesized chemically, using ammonium persulfate as an oxidizing agent, in the presence of hexadecyltrimethylammonium bromide, as a surfactant. A homogeneous sulfur layer was chemically deposited on the PPy nanofibers. The nanofibers had a diameter of 50–70 nm and a highly rough surface, but when covered with sulfur, they became smoother [170]. The highlight of this study does not lie in the way the nanostructures were prepared but in how these materials were used in a lithium-sulfur battery. A PPy@S-based cathode deposited on an electrosynthesized PPy film was designed, which was assembled into a commercial separator covered with PPy nanofibers. Therefore, not only a nanostructured and independent cathode was achieved without the need for other current collectors, but also a separator was obtained that acted as a polysulfide scavenger and enhancer of the cathode capacity. This made it possible to attenuate the “shuttle effect,” characterized by the dissolution of lithium polysulfides, which reduces the battery’s cycling capacity. In addition, the device was subjected to curvature tests, demonstrating good flexibility [170]. 

In the field of nanocomposites based on PPy and inorganic materials, there are several studies that demonstrate the advantages of incorporating metal oxides. Regarding the formation of two-dimensional nanostructures, the achievement of interspersed nanosheets or nanosheets of PPy and metal oxides is noteworthy. Tang et al. [171] reported the design of an intercalated structure composed of PPy nanolayers on MoS_2_ monolayers. The nanocomposite was prepared by chemical polymerization of the pyrrole monomers, using ammonium persulfate as an oxidant, in the presence of MoS_2_. Thus, the monolayers of this inorganic compound, up to 1.4 nm thick, functioned as templates, where the ultra-thin layers of PPy were intercalated. Depending on the mass ratios of MoS_2_ and pyrrole with which the nanocomposites were prepared, different thicknesses of the hybrid MoS_2_/PPy layers were achieved, reaching up to 15 nm. By being able to control the thickness of the nanolayers, the amounts of MoS_2_ and pyrrole used were optimized to achieve a nanocomposite with good performance as an electrode in a supercapacitor. Improvements in properties such as electrical conductivity, surface area, mechanical resistance, and energy storage capacity were demonstrated with respect to its components separately.

Inorganic materials such as metals have been difficult to use in energy storage applications, either because of cost or because of the difficulty of optimizing their properties to obtain good yields. For this, the combination with PPy forming stable nanostructures can allow taking advantage of the properties that a metal and a CP grant. Within this scope, Moon et al. [172] reported the synthesis of a network of Ag/Au/PPy-based nw with a core-shell type structure. The 30 nm diameter Ag nw was covered by a 3–5 nm thick Au layer through a chemical process in an aqueous solution. Then, on this structure, the PPy nanolayer was electrodeposited until reaching Ag/Au/PPy nw of ~100 nm, forming a matrix with mesh morphology. One of the keys to the formation of this nanocomposite lies in the incorporation of the Au nanolayer. Ag nw is not compatible with the electropolymerization of pyrrole since Ag is oxidized at a potential of 0.8 V (vs. SHE), which in turn corresponds to the oxidation potential of pyrrole [172]. Instead, Au is oxidized at a potential of 1.5 V (vs. SHE), which allowed the formation of the PPy electrodeposit at 0.8 V. Although a matrix based on Ag nw demonstrated high electrical conductivity, optical transparency, and mechanical flexibility, the incorporation of a PPy nanolayer, assisted by the Au nanolayer, allowed to obtain a material with better electrochemical and mechanical performance when tested as an electrode of a supercapacitor [172]. This has demonstrated the advantages that emerge from combining both the nanostructuring of materials, as well as the synergistic effects between materials of different types.

### 6.3. PTh 

The studies related to the nanostructuring of PTh, with a view to using it in energy storage electrodes, are less in relation to those of other polymers. However, it is worth highlighting some significant research due to its great contribution to the development of nanostructures. For example, already at the beginning of the previous decade, Fu et al. [173] showed the design of PTh nanotubes and microtubes, electrochemically synthesized, using microporous alumina membranes 60 µm thick and pores with a diameter of 20–200 nm. Thanks to such templates, aligned nanotubes were achieved, with an orderly and uniform distribution, on a thin layer of Au. When comparing the cyclic voltammograms of the nanotube-PTh/Au systems, microtubes-PTh/Au, and a PTh/Pt film, higher responses were obtained for the first two. Mainly, this would be explained by the increase in the surface area and in the electrochemically active sites in contact with the electrolyte, compared to the PTh bulk film [173].

Another one-dimensional (1D) nanostructure of great relevance in this field corresponds to nw. With regard to the synthesis methods, it is possible to highlight what was performed by Karim et al. [174], where the synthesis of PTh nw was reported by a method that involved oxidative chemical polymerization and gamma radiation, which is called radiolysis polymerization. Through images obtained by FE-SEM, nw of 50–100 nm in diameter were observed, distributed in a disorderly way. The use of gamma radiation was what allowed self-assembled nanostructuring without the need for templates. On the other hand, chemical polymerization without radiation only allowed to obtain of the bulk material [174]. Although the nanostructure obtained was not tested for any application, it stands out as one of the first attempts at the nanostructuring of this CP.

Subsequently, our research group reported for the first time obtaining PTh nw using only electrochemical methods, using a mesoporous silica template. First, a silica film was potentiostatically deposited on a glass electrode covered with SnO_2_-F; then, on this modified electrode, the electropolymerization of thiophene was carried out, which grew within the spaces confined by the pores of the silica template. When comparing the electrochemical responses of this material with those of PTh bulk, it was found that they were significantly higher for the first case. This method is characterized by its simplicity, low cost, and the possibility of nanostructuring a wide variety of CP in a highly reproducible way [90,175]. In this sense, it is also worth highlighting the possibility that this method offers to work in a totally anhydrous medium, which is essential to achieve reproducibility in cases of monomers that oxidize at higher potentials, such as thiophene, where water, even in quantities minimal, interferes greatly.

Regarding the formation of PTh nanocomposites with carbonaceous materials, the work of Fu et al. [176], where the electropolymerization of thiophene on multi-walled carbon nanotubes (MWCNT) in an ionic liquid (bmimPF_6_) was shown for the first time. By means of SEM images, the structure of PTh was compared on an MWCNT/glassy carbon electrode and another only one of glassy carbon. In the first case, nanofibers interwoven with the MWCNT surfaces were obtained, achieving a rather porous structure. In the second case, stacked films were obtained, forming a dense layer of PTh. Therefore, the nanocomposite based on MWCNT/PTh proved to be more apt to be applied as an electrode material in an energy storage device, given the optimization of the surface area and the best electrical conductivity delivered by the synergy between the components [3].

Also, in combination with carbonaceous materials—and taking advantage of their porosity—there are approximations with a more two-dimensional PTh morphology, acting as a nanolayer. Nejati et al. [177] showed the formation of nanocomposites based on a high porosity material on which PTh nanolayers were deposited. This was performed by oxidative chemical vapor deposition of the thiophene monomers, using ammonium pentachloride as the oxidizing agent. Good control was achieved in the deposition of the PTh layers, its thickness being able to vary within a wide range, reaching ultra-thin layers of even ~4 nm. The porous substrates on which the PTh layers were deposited were anodized aluminum oxide, a matrix of TiO_2_ nanoparticles, and activated carbon. An attempt was made to optimize the thickness of the PTh nanolayers so as not to completely obstruct the porosity of the substrate. In this way, nanocomposites of high porosity and surface area were achieved when compared with samples based on thicker and flatter layers of the polymer. Due to these characteristics, a composite of PTh nanolayers on activated carbon was tested as an electrode material in a supercapacitor, developing up to 1.5 times more capacitance compared to PTh layers and allowing an optimized diffusion of BF_4_^−^ anions to the active sites of the electrode [177]. 

Nanostructures incorporating PTh with layer morphology can also be found by combining it with inorganic materials, such as metal oxides. For example, Lu and Zhou [178] show the preparation, in a single container, of a nanocomposite based on MnO_2_/PTh, with a hierarchical structure of the sub-micrometric spheres/nanosheets. An organic/inorganic interface polymerization was carried out simultaneously occurring the oxidation of the thiophene monomers (forming PTh) and the reduction in MnO_4_^−^ (forming MnO_2_). According to images obtained by FESEM, the spheres whose diameters ranged from 500 to 800 nm consist of radial nanosheets with a thickness of less than 10 nm and an abundant distribution of pores, with up to 4 nm in diameter. Therefore, by means of a simple method, a nanostructured hierarchical structure was achieved, suitable for applications in the field of sensors, catalysts, and energy storage.

In addition to structures forming intercalated layers, one-dimensional nanostructures of a metal oxide can be found in combination with PTh. For example, Ambade et al. [179] reported the synthesis of a nanocomposite based on TiO_2_ nanotubes (TNTs) and PTh embedded in its pores. The TNTs were manufactured by anodization in two stages, in a water-free electrolyte, obtaining a highly ordered and compact structure of nanotubes. Then, on this arrangement, the electropolymerization of thiophene was carried out, which infiltrated the nanoporous matrix of TNTs. TEM images revealed a continuous structure with uniformly distributed fragments of PTh on the surface of the TNTs, ~100 nm in diameter. The PTh nanofibers embedded in the mesopores of TNTs made it possible to optimize the surface area, which would be beneficial for faradaic redox processes by increasing the number of active sites. Furthermore, the synergy between PTh and TNTs allowed better mechanical stability and electrical conductivity with respect to the separate components. This was verified by testing the electrochemical performance of the nanocomposite, participating as electrodes of a supercapacitor.

Returning to the function of PTh as a nanolayer, in terms of core-shell type nanostructures, some important contributions can be found at the beginning of the decade. It is worth mentioning what was reported by Wu et al. [180], who synthesized core-shell composites where a sulfur microsphere was covered with a PTh nanolayer. The S-PTh composite was prepared for different mass ratios between sulfur and PTh. The chemical polymerization of the thiophene monomers was carried out using FeCl_3_ as an oxidant in the presence of sulfur. The sulfur particles had the crown structure of S8 and self-aggregated to form microspheres of approximately 20 µm. On the other hand, the PTh nanolayers presented abundant porosity and reached a thickness of approximately 20–30 nm. This structure allowed the protection of sulfur, preventing its dissolution in the electrolyte, in turn facilitating the insertion/disinsertion of ions. In this way, the material was studied as a cathode in a Li-S-type battery.

Along with the classical investigations of core-shell type nanostructures, generally based on sulfur centers, they have been developed with other inorganic materials, given the advent of new types of batteries, such as sodium or potassium batteries. For example, Ali et al. [181] reported the preparation of a core-shell type material composed of olivine phase NaFePO_4_ particles covered by a PTh nanolayer (NaFePO_4_@ PTh). The method consisted of LiFePO_4_ delithiation, subsequent coating with PTh added as dispersion and subsequent insertion of Na^+^ ions into the structure. The PTh was synthesized via in situ polymerization prior to its use as a coating. The NaFePO_4_ particles showed sizes from a few hundred nm down to a micrometer scale, while the PTh nanolayers had thicknesses of 5 to 12 nm. In addition, these nanolayers allowed the olivine phase NaFePO_4_ particles to interconnect, increasing not only the mechanical stability but also the electrical conductivity of the material. The use of olivine phase NaFePO_4_ has been proposed as an option in sodium battery electrodes. However, it has a high resistance to charge transfer, the diffusion of Na^+^ is low, and it is prone to degradation in Na^+^ insertion/extraction processes. The incorporation of a porous PTh nanolayer showed better performance compared to NaFePO_4_ in terms of better energy storage capacity, higher electrical conductivity, ionic diffusion, and stability of the NaFePO_4_ core. 

### 6.4. PEDOT 

Moreover, among PEDOT’s variety of nanostructure designs, one-dimensional ones can immediately be positioned as optimal candidates in sensor or energy storage applications. However, problems of mechanical stability and reduction in the surface area used arise when self-aggregation phenomena occur. This can be safeguarded by incorporating other materials and/or achieving high-order structures. For example, in this pathway, Liu et al. [182] reported the electrochemical synthesis of PEDOT nanotubes, assisted by an alumina membrane template with cylindrical pores. An ordered arrangement of high conductivity nanotubes, with ultra-thin and porous walls, was obtained on an Au film. In order to study this nanostructure as a possible electrode candidate for a supercapacitor, nanostructuring was carried out with the alumina membrane included to prevent the self-aggregation that the nanotubes can undergo. The hollowed-out morphology of the nanotubes allowed easy insertion/disinsertion of ions from the Et_4_NBF_4_ electrolyte. When comparing with an electrode based on nw enclosed by an alumina membrane, higher charge storage was obtained for the case of nanotubes. In this way, the rational design applied to an electrode based on PEDOT allowed a better performance in terms of kinetics and stored energy. However, the requirement of alumina as mechanical reinforcement to attenuate the degradation of the polymeric material showed that nanostructuring is not enough if it is not optimized, in addition, depending on the design that considers better mechanical stability.

In the field of sensors, under the strategy of optimizing performance through nanostructuring, a work by our group [183] can be highlighted, in which using only electrosynthetic methods, PEDOT nw (PEDOT-nw) of diameter in a range 6.7–13.9 nm were obtained with the assistance of SiO_2_ as a template, on Pt substrates. PDA was electrodeposited on Pt/PEDOT-nw type electrodes to obtain a sensor capable of selectively detecting dopamine in the presence of uric acids and ascorbic. Compared to its bulk Pt/PEDOT/PDA counterpart, the cyclic Pt/PEDOT-nw/PDA voltammograms showed a higher current response. Additionally, for a concentration of 1.0 µM of dopamine, the Pt/PEDOT-nw/PDA electrodes presented linear calibration curves with LOD and LOQ of 0.47 and 1.49 µM, respectively. On the other hand, for its counterpart in bulk, non-linear curves were obtained, and the values of LOD and LOQ could not be determined. Consequently, obtaining sensors by simple electrochemical methods was demonstrated, the nanostructuring of which markedly improved the ability to selectively detect dopamine.

Based on the benefits of electrochemical synthesis techniques to achieve 1D nanostructures, Hsu et al. [184] reported the electrochemical synthesis of PEDOT nw on a conductive carbon fiber (CC) fabric substrate. EDOT’s electropolymerization process consisted of stages: (i) nucleation by cyclic voltammetry and (ii) growth of nw by galvanostatic pulse. By means of FESEM images, a dense arrangement of nw was observed, with a diameter of 60–80 nm and a length of several micrometers. In addition, depending on the time used for the growth of the nw, the porosity of the material could be controlled. That is, it was concluded that an excess of synthesis time caused auto aggregation of the nw, the optimal growth condition being only five cycles by cyclic voltammetry plus 5 min of the galvanostatic pulse. In general, the PEDOT/CC nanocomposite demonstrated a greater surface area and greater energy storage capacity compared to an electrosynthesized PEDOT film on CC. The good performance of PEDOT/CC was due to a better penetration of electrolytes based on Na_2_SO_4_ or H_2_SO_4_, greater use of redox-active sites, and the high electrical conductivities of both PEDOT and carbon fibers.

In the previous case, there was the incorporation of a carbonaceous material as a current collector, optimizing the electrical conductivity. In this regard, it is worth mentioning other studies that mix PEDOT with carbonaceous materials. For example, Alvi et al. [185] presented the preparation of a nanocomposite based on graphene and nanofibers from PEDOT. By chemical polymerization, using APS and FeCl_3_ as oxidants, a G/PEDOT nanocomposite was obtained with a high surface area and abundant porosity. PEDOT nanofibers ~30 nm in diameter were formed between the graphene nanofibers, which, when aggregated, could form fibers of 100–200 nm in diameter. Not only the morphology of the material but also the synergistic effect between PEDOT and graphene allowed us to obtain a good performance when testing it as a supercapacitor electrode. Compared to a pristine PEDOT film, the nanocomposite demonstrated better electrical conductivity and greater ease of ion transport to electrochemically active sites, reflected in greater energy storage capacity, fast kinetics, and greater mechanical stability.

In the same area, Liu et al. [186] prepared a composite in the form of a thin sheet, based on reduced graphene oxide (rGO) and PEDOT/PSS, by a simple rod coating method. Commercial PEDOT/PSS pellets were used, which were mixed with graphene oxide dispersions. Therefore, a polymerization process was not carried out. Then, it was subjected to reduction treatment, and the coating was formed on PVDF, which could then be easily removed to obtain a flexible and independent structure. In this case, the nanostructuring occurs within the rGO-PEDOT/PSS films since among the rGO nanofibers, some species of PEDOT/PSS nanofibers were intercalated. This increased the space between rGO nanosheets and, in addition, managed to attenuate the usual self-aggregation that occurs in them. Given the achievement of a three-dimensional nanostructuring, an optimization of properties such as mechanical flexibility, abundant porosity, high surface area, and less self-aggregation was obtained. For these reasons, this material was tested as a supercapacitor electrode. When comparing with rGO and pristine PEDOT/PSS, the importance of optimizing the sizes of the nanochannels or nanopores and the impediment of self-aggregation was verified, allowing a good ionic diffusion and abundant active sites for redox reactions.

In addition to PEDOT and carbonaceous materials such as graphene, the synthesis of ternary nanocomposites has been possible, incorporating some metallic oxide. In the field of energy storage, metal oxide can increase the pseudocapacitance and have some mechanical role. Wang et al. [187] manufactured a ternary nanocomposite based on graphene, SnO_2_, and PEDOT, chemically through a one-pot synthesis. It was possible to intercalate nanoparticles of PEDOT and SnO_2_ between the nanofilms of G, which has been shown as a good strategy for preventing their self-aggregation. When compared to a G/SnO_2_ nanocomposite, the G/SnO_2_/PEDOT-based nanocomposite revealed a thicker and smoother appearance. The ternary nanocomposite showed an optimized surface area and abundant channels for electrolyte diffusion between the G nanofilms. The material was tested as a supercapacitor electrode, demonstrating good mechanical stability and electrochemical performance due to the synergy between the components and the design of high porosity nanostructured.

As has happened with other CPs, the core-shell type design has also been extended in the case of PEDOT, forming different hybrid nanostructures. For this type of design, work is performed—as in other polymeric materials—mainly combining the polymer with inorganic materials. Among the publications in this regard, the studies by Han et al. [188], where the preparation of a core-shell type nanoplatelet array (NPA) based on laminar double hydroxides (LDH) and PEDOT (LDH@PEDOT NPA) has been demonstrated. The method was based on two main stages: (i) formation of LDH hydrothermally, using Al and Co nitrates, on Ni foam; (ii) electrosynthesis of a ~6 nm nanolayer of PEDOT on LDH. By means of TEM images, the hexagonal shape of the nanoplates was observed, with a diameter of 4–5 µm and a thickness of ~40 nm. In addition, channels suitable for electrolyte transport were obtained between the nanoplates. The nanocomposites were studied as supercapacitor electrodes, showing high electrical conductivity, fast kinetics, good mechanical stability, and flexibility compared to their separate components. In this, the optimization of the surface area, the space between nanoplates, and the core-shell type conformation have been fundamental.

On the other hand, the combination of PEDOT and S has become common, as can be seen in studies by Chen et al. [189], where the synthesis of ultrafine S nanoparticles covered by PEDOT nanolayers was reported, forming a core-shell type structure. The S nanoparticles were synthesized by a hollow fiber membrane-assisted precipitation procedure. On the other hand, the PEDOT nanolayers were synthesized and deposited by polymerizing EDOT by chemical means, using FeCl_3_ as an oxidizing agent. Through images obtained by TEM, the formation of spheres of S was observed, reaching diameters of 10 to 20 nm. On them, PEDOT nanolayers of ~5 nm thick were obtained, with a high porosity structure. On the one hand, the ultra-small size of the nanoparticles facilitates electronic transport, while the PEDOT nanolayer acts as a protection that prevents the loss of polysulfides from the interior of the structure and, at the same time, allows the insertion/disinsertion of ions, particularly Li^+^. In addition, a material with a high surface area was obtained, with abundant redox active sites. For this reason, this nanocomposite was studied as a cathode in a lithium-sulfur battery, obtaining outstanding charge storage capacity and mechanical resistance when compared with its components separately.

Other designs of core-shell type nanostructures that contain PEDOT and some inorganic material may contain some doping modifying agent. For example, Zeng et al. [190] reported the rational design of core-shell nanorods based on crystalline Fe_2_O_3_ doped with Ti and wrapped in a PEDOT nanolayer (Ti-Fe_2_O_3_@PEDOT) on a carbon fiber fabric. The synthesis process was carried out in two stages. First, the Ti-Fe_2_O_3_ nanorods grew uniformly and directly by means of a hydrothermal method on the carbon tissue, reaching a diameter of ~50 nm. Second, the nanorods were covered with an electrochemically deposited layer of PEDOT. Through TEM images, the amorphous nanolayer of PEDOT was observed, estimating a thickness of 5 nm. The Ti-Fe_2_O_3_@PEDOT nanocomposite was demonstrated to have a high surface area and numerous channels for ionic diffusion, for which it was tested as an electrode in a supercapacitor. The results demonstrated a better performance of the nanocomposite compared to pristine Fe_2_O_3_ and Ti-Fe_2_O_3_. On the one hand, doping with Ti improved the electrodonating capacity of Fe_2_O_3_ and, on the other, the PEDOT nanolayer increased electrical conductivity, allowed less degradation of the material by acting as a cover and increased the number of electrochemically active sites.

Within the field of ternary nanocomposites, where PEDOT acts as a protective layer, Su et al. [191] reported the preparation of a composite based on nanocrystals of a Prussian blue analog, Na_2_Fe[Fe(CN)_6_], embedded with S and covered by a layer of PEDOT(S@Na_2_Fe[Fe(CN)_6_]@PEDOT). Both the nanocrystals and the PEDOT shell were prepared chemically, obtaining nanocomposites of ~100 nm. Although the thickness was not determined for the PEDOT coverage, it can be inferred that it is on a nanometric scale. In addition to enhancing the electrical conductivity of S@Na_2_Fe[Fe(CN)_6_], this PEDOT nanolayer functioned as a mechanical stabilizer and prevented the dissolution of polysulfides stored in S@Na_2_Fe[Fe(CN)_6_] (shuttle effect). This is important, considering that this nanocomposite was designed as a cathode for a lithium-sulfur battery. The nanocomposite demonstrated greater reversibility, fast kinetics, and good mechanical stability compared to S@Na_2_Fe[Fe(CN)_6_], being the stabilizing nanolayer of PEDOT the key. 

Other approaches, beyond core-shell type structures, have considered PEDOT as a protective and conductive nanolayer, especially in the design of battery electrodes. For example, Shang et al. [192] reported the preparation of a monolithic composite based on Ni_3_S_2_ and PEDOT. On Ni foam, Ni_3_S_2_ was synthesized hydrothermally, and a PEDOT nanolayer was electrodeposited on its surface. The monolithic structure of Ni_3_S_2_ obtained an internal morphology composed of microparticles between 100 and 400 nm. On the other hand, the PEDOT nanolayer showed a nano-flake morphology with abundant porosity. The monolithic structure of Ni_3_S_2_, with high porosity, allows efficient electron transport, greater use of electrochemically active sites, and easy transport of ions such as Na^+^ or Li^+^. However, ion insertion/disinsertion degrades the material easily. In this, the incorporation of the PEDOT nanolayer has been fundamental, not only providing greater electrical conductivity but also protection and greater mechanical stability. During the expansion and contraction processes of an electrode based on Ni_3_S_2_@PEDOT in a sodium battery, the nanolayer allowed better flexibility and cycle stability when compared to a monolithic Ni_3_S_2_ electrode without PEDOT.

Among the flexible nanocomposites that incorporate PEDOT and that are studied within the field of energy storage, studies such as those by Zhao et al. [193], where the design of a flexible composite based on a cellulose/PEDOT:PSS matrix reinforced with MWCNTs was reported. In the ionic liquid 1-butyl-3-methylimidazolium chloride ([bmim]Cl), a supramolecular assembly was formed with EDOT and cellulose monomers, which guided the in situ chemical polymerization of PEDOT and PSS in the presence of MWCNTs. By means of images obtained via SEM, the formation of cellulose nanofibers/PEDOT:PSS reinforced with MWCNTs was observed, with mesopores of ~20 nm massively distributed. Compared with PEDOT:PSS films, the porosity of the prepared nanocomposite was much higher. By optimizing the amount of MWCNTs, which is fundamental for electrical conductivity, the highest value was obtained for 7% by mass of MWCNTs. Furthermore, for the nanocomposite with said composition, a breaking stress of 95.8 MPa and Young’s modulus of 3.51 Gpa were obtained. Given these characteristics, the nanocomposite was tested as an electrode in a flexible, solid-state supercapacitor. The device did not decrease its operability under deformation and demonstrated a higher energy storage capacity than other PEDOT-based devices. This is a case that exemplifies the participation of nanostructured PEDOT in a tertiary and flexible hybrid material, providing active redox sites and electrical conductivity.

### 6.5. Copolymers 

Some research, since the early 2000s, has been concerned with developing lightweight, ultra-thin, and high-conductivity materials for CP-based electronic applications, such as transistors or LEDs. For this, it has been important to obtain highly ordered conjugated polymer chains so that their electrical conductivity is optimized. An example in this area is a study by Liu et al. [194], where synthetic routes were applied to obtain block copolymers and polyurethane elastomers containing regioregular PTh derivatives, exhibiting nw-shaped structures with good electrical conductivity. For example, in one of the structures, a solvent-casting method was applied, obtaining poly(3-hexylthiophene)-*b*-polystyrene (PHT-b-PS) with a structure of nw separated laterally by 30–40 nm, reaching an electrical conductivity of 4.7 S cm^−1^. The formation of ordered supramolecular structures, by self-assembly of regio-regular chains and a good control in the evaporation of the solvent used were shown as key factors to achieve the polymeric nw. In addition, the importance of forming copolymers with polystyrene was based on the improvement of the mechanical properties of the material, compared to the mere use of regio-regular PTh derivatives. 

Regarding the obtaining of nanostructured copolymers that exhibit some functionality for potential applications, there are reports from the previous decade. In the field of copolymers based on an aniline monomer and another from a different class, the example of Mu et al. [195] can be found, who reported the synthesis by electrochemical route of poly(aniline-*co*-*o*-aminophenol), starting from aniline and *o*-aminophenol, in the presence of ferrocene sulfonic acid, forming a network of nanofibers with average diameters in the range of 70–109 nm, on a Pt substrate. By controlling the diameter of the nanofibers, from the CV cycles, its influence in relation to the electrocatalytic oxidation of catechol was studied. When comparing the CV results, it was obtained that the smaller the diameter and, therefore, the greater the surface area, the lower the anodic oxidation potential of catechol (up to 0.427 V vs. SHE). On the contrary, the smaller the surface area, the more easily polarized the electrode became, so the potential was shifted to more positive values (up to 0.485 vs. SHE). In addition, its electrocatalytic activity showed a dependence on pH and the presence of ferrocene sulfonic acid, being better at pH ≤ 9.0. The above demonstrated how significant the synthesis and operation conditions of the electrocatalyst were, as well as its morphology, in terms of its surface area.

In the field of CP intercalated with 2D carbonaceous materials, there have been relatively fewer reports based on copolymers that achieve structural, pseudocapacitive, electrically conductive, and mechanical stabilizing functionality. Using a container copolymer of aniline and graphene oxide (GO) sheets, Wang et al. prepared an embedded design nanocomposite. The copolymer poly(aniline-*co*-*o*-anisidine)—P(An-co-oAs)—was synthesized by the oxidative chemical route and by means of a delamination/reassembly method, it was intercalated between the GO nanosheets, forming monolayers nanostructured confined to a space of ~1.1 nm. This design allowed for better electrical conductivity and optimal spacing for ion insertion. Therefore, this design falls into the category of those CP that are nanostructured by the presence of another structure, which acts as a template or substrate but is also usable as a functional component (without being removed). The incorporation of poly (aniline-*co*-*o*-anisidine) in the form of nanosheets increased the electrical conductivity (from 4.8·10^−5^ to 1.9·10^−1^ S cm^−1^), increased the electrochemical response in a given cyclic voltammogram a better insertion of Li^+^ ions, and allowed a good electrochemical and mechanical stability, after a few cycles of operation1.

Another similar case of a nanocomposite with intercalated structures corresponds to that reported by Yang et al. [196], although in this case, an inorganic material was used, namely MnO_2_ sheets and the P(An-co-oAs) copolymer, synthesized by oxidative chemistry. The MnO_2_/P(An-co-oAs) nanocomposite was manufactured through a delamination/reassembly process, exhibiting a morphology of MnO_2_ nanosheets interspersed with P(An-co-oAs) monolayers in a confined space of 1.65 nm. This structuring allows better ionic diffusion, greater electrical conductivity, greater surface area, and good mechanical stability during ion insertion/disinsertion processes. This was reflected when comparing with samples of P (An-co-oAs) and Na-MnO_2_. Separately, the nanocomposite showed a greater electrochemical response in CV. Therefore, when studied as electrodes of a supercapacitor, they allowed the obtaining of higher capacitance.

For the development of cathodes in rechargeable batteries, organic materials with radical and carbonyl groups have been investigated since they have been shown capable of storing ions such as Li^+^ or Na^+^. To increase their stability and specific capacity, various research groups have resorted to modifications of such organic structures and to perform tests under different conditions. An example corresponds to the synthesis of anthraquinone-based CP, for their use in LIBs cathodes, according to that reported by Xu et al. [197]. Two poly (anthraquinonyl sulfide) derivatives were synthesized. Namely, poly(1,5-anthraquinonyl sulfide) and poly(1,8-anthraquinonyl sulfide), abbreviated as P15AQS and P18AQS, respectively. Both polymers showed an amorphous morphology of agglomerated nanoparticles, with sizes of 30–50 nm for P18AQS. Beyond this nanostructuring, there are other factors that were considered to evaluate its electrochemical performance. The effect of the position of the sulfur substituent, the type of binder, and the electrolyte used were evaluated. For example, P15AQS reached higher specific capacities for the storage of Li^+^, with respect to P18AQS, due to the lower steric hindrance due to the position of the sulfur substituent. Furthermore, the use of PVDF as a binder and of ether-based electrolytes allowed the best performances in both polymers. Thus, in addition to the nanostructured morphology that the polymeric material may exhibit, there are other key factor modifications that optimize its performance.

Polymers derived from anthraquinone can also be obtained by electrosynthetic methods, as demonstrated in a study by our group [175,198]. Applying a fixed potential, nw of ~30 nm in diameter of poly(1-amino-9,10-anthraquinone) (P1AAQ) were electrodeposited on a stainless steel substrate modified with a thin film of poly(1AAQ-*co*-*o*-phenylenediamine) (P(1AAQ-co-o-PD)), with the aid of a SiO_2_ template. The P(1AAQ-co-o-PD) film allowed the fixation of the nw through chemical bonds, which gives it excellent stability with respect to the direct deposit of the nw on the metal electrode. On the other hand, once the SiO_2_ template had been removed, a highly ordered nw structure was obtained, optimizing the surface area, and presenting higher electrochemical responses in CV, compared to the electrode modified with P1AAQ bulk. It is worth noting the reproducibility of the nw arrangement (something unusual), considering the anodic and cathodic peak potentials, where standard deviations of 3.357·10^−6^ and 3.901·10^−6^ were obtained, respectively (with *n* = 10). Regarding stability, the current density response only decreased by 1.5% after 10 cycles and, even more importantly, by ~30% between cycles 20 and 100. In addition, when performing dsDNA detection assays in solution, we obtained an increase in current density with increasing concentration. In fact, in a subsequent study, the P1AAQ nw array was systematically evaluated as a biosensor for the detection of ssDNA, with detection and quantification limits of 5.7·10^−12^ and 1.9·10^−11^ g L^−1^, respectively [198]. Due to the simplified method used in the manufacture of a reproducible nanostructure and the evident improvement in the electrochemical responses related to the optimization of the surface area, this type of design strategy can be expanded to other types of CP or copolymers to be tested in applications.

### 6.6. CP Conjugated with Carbonyl Functional Groups 

Simultaneously with the investigation of CP conjugated with carbonyl functional groups, structures based on compounds called polycyclic aromatic hydrocarbons (PAHs) have been studied for their potential application in energy storage systems. One of the first reports in this way corresponds to the work of Bachman et al. [199], where nanostructured electrodes for supercapacitors are manufactured, made up of electropolymerized pyrene derivatives and some-walled carbon nanotubes (FWCNTs) functionalized with oxygen-containing groups on their surfaces. The polymers obtained were poly(aminopyrine), polypyrene, and poly(pyrene carboxylic acid), which adopted different morphologies depending on the substituents. In turn, this had an impact on electrochemical performance, as they were tested as supercapacitor electrodes. In this sense, we can highlight poly(aminopyrine), whose better performance could be attributed to its nanostructuring, adopting a tight coverage on the surfaces of FWCNTs, facilitated by the strong electrostatic interactions between the COO^−^ substituents of the carbonaceous material and the NH_3_^+^ groups of the protonated polymer. Thus, the surface area and sufficient space were optimized for the good insertion of Li^+^ ions toward redox active sites. On the contrary, the other two polymeric structures formed more micrometric agglomerations, with more irregular coverage, even inside the nanotubes, in such a way that it had a negative impact on their performance. Thus, although the three polymers had conjugated structures, the type of substituents and their consequent nanostructuring on the FWCNTs were of great importance.

The CP pyrolysis strategy to obtain N-doped carbonaceous materials with electrocatalytic activity for ORR has also been applied to derivatives of conventional CP. An outstanding study in this area was the one carried out by Liang et al. [200], where an ORR electrocatalyst was obtained based on hierarchically porous carbon doped with N. For its manufacture, it was used as a precursor to chemically synthesized poly(*o*-phenylenediamine) (PoPD), with the assistance of a hard template of SiO_2_. Subsequently, the PoPD/SiO_2_ composite was subjected to pyrolysis and activation processes with NH_3_, producing a 3D meso/microporous nanostructure with layers 1–3 nm thick. Compared to a mesoporous sample synthesized without the assistance of SiO_2_ colloids, the surface area of the meso/microporous nanostructure was up to four times greater (1280 m^2^ g^−1^), showing the relevance of the formation of micropores (1.4 nm in diameter). This morphological optimization had a fundamental impact when evaluating the nanostructures in terms of electrocatalytic activity for ORR. Not only was the content of the active N sites important, but also their greater availability in the meso/microporous structure, also allowing a better transport of O_2_ within the electrocatalyst.

## 7. Nanostructured CP as Supercapacitors 

Among electrochemical energy storage devices, supercapacitor electrodes have been the preferred applications for testing various nanostructured CP designs. This has been intended to capture in this compilation, where it has been chosen to analyze and discuss, mainly, research from 2018–2019, since—as will be evident—the number of publications in this area is too extensive. In this way, an updated view of the trends in the use of nanostructured CPs in supercapacitors can be achieved. In terms of the electrochemical performances in electrodes of these devices, different bibliographic reviews have shown the advance that has meant the nanostructuring of polymeric materials, forming or not, hybrids with other materials [39,41,121,201,202]. 

Some theoretical values of specific capacitance that have been determined for PANI, PPy, PTh, and PEDOT correspond to 750, 620, 485, and 210 F g^−1^, respectively [203,204]. This partially explains the preference for PANI for use in supercapacitors. Although they are values that have been exceeded, depending on the optimizations to which these materials have been subjected, they provide an order as to who is potentially better to store energy.

### 7.1. PANI 

The nanostructuring of PANI has already made it possible some years ago to achieve a specific capacitance of 950 F g^−1^ (at 1 A g^−1^) and an energy density of up to 130 Wh kg^−1^ when evaluating a vertical nw arrangement [205]. However, a significant loss of capacitance was verified during the first 100 cycles (16%) in HClO_4_ aqueous electrolyte, demonstrating once again the structural degradation of this class of materials during the charge/discharge processes. Although some strategies to improve long-term stability are based on using non-protonated electrolytes, ionic liquids, or optimizing the potential operating window, this can cause worse energy storage.

On the other hand, what the present review shows regarding nanostructured CP in supercapacitors are that the mixture with carbonaceous or inorganic materials, the incorporation of dopants, pyrolysis, or the formation of hydrogels have been the preferred strategies to improve the electrochemical yields in parameters such as specific capacitance, speed capacity or long-term structural stability. However, not necessarily in all cases, the desired optimization has been achieved. Furthermore, the emergence of research areas framed in the design of flexible, self-repairing, multifunctional, or small-scale devices (microsupercapacitors) has required the combination of nanostructured CP with other materials. Otherwise, using only CP, it is not possible to achieve the properties necessary for the proper functioning of such devices.

The combination of CP with materials such as GO, rGO, or CNTs has led to the largest number of publications selected in this review. On the one hand, GO, or CNTs, allow to increase in the surface area and provide electron-conducting carbonaceous structures, and their functional groups act as anchors for the nucleation and growth processes of the chains and mechanically stabilize the CP. On the other, the latter provide active redox sites to store charge, can enhance electrical conductivity—especially PEDOT and other derivatives of PTh [41] and prevent the self-aggregation of nanofilms or nanotubes of those carbonaceous materials. Other improvements can be achieved by increasing conductivity through reduction to obtain rGO or graphene without functional groups, optimization in the number of materials used, depositing nanocomposites on flexible substrates—such as SS, PET, CC, or CF—the inclusion of noble metals, synthesis of hydrogels, subjecting to pyrolysis to form doped carbons, among others.

In turn, either with such types of nanocomposites or only with nanostructured CP, the research groups that work with inorganic compounds have tested the inclusion of those materials to enhance the yields of metal oxides, hydroxides, sulfides, or selenides and materials, such as MXenos or LDH, in supercapacitor electrodes. The integration of CP—with or without carbonaceous materials—can give more structural stability, electrical conductivity, active redox sites, and less self-aggregation of the components. This allows electrodes to achieve better performance, compared to the only use of inorganic materials, with greater long-term stability and even higher levels of charge storage. Compared with some electrodes based on inorganic materials, several reports considered here demonstrate performance parameters at the same or higher level [206,207].

Other classes of polymeric nanostructures have also been considered in this review. For example, the use of traditional CP to form carbons doped with N or S, hydrogels with different modifiers, and combination with materials derived from biomass and mixtures of these polymers. Other categories are copolymers derived from traditional CP, with other substituent groups, polymers with carbonyl groups, and 2D or 3D microporous polymeric structures of COFs and CMPs. All these varieties, which here have been grouped into different categories, account for the wide range of polymeric nanostructures that have exhibited equal or higher levels, in specific capacitance, energy density, long-term stability, and speed capacity, with respect to polymeric structures, more conventional. More details about the recent uses of PANI, PPy, PEDOT, and other polymer nanostructures will be developed below.

PANI nanostructures used in supercapacitors, which are reviewed below, have been classified into the following categories: combined with carbonaceous materials (Table 1); combined with inorganic materials (Table 2, entries 1–10); in hybrids with carbonaceous and inorganic materials (Table 2, entries 11–24) and other nanostructures (Table 2, entries 25–41).

The mixture of PANI with carbonaceous materials in various nanostructures is part of most of the publications in the field of CP used in supercapacitors. Here, nanostructures composed of PANI and some carbonaceous materials have been considered, which include (or not) another modifying component of a more auxiliary nature, namely, current collectors, binders, or dopants, which enhance some desired properties (Table 1). Moreover, carbon-based materials that come from biomass will be addressed later. 

The incorporation of PANI on materials such as rGO, GO, or graphene (G) may not only be convenient due to its synergy in terms of greater electrical conductivity but also demonstrates the feasibility that a substrate can act as an optimizing template for some properties. This kind of electrically conductive template is incorporated into the hybrid material, anchoring PANI through p-p interactions, contributing to mechanical stability and electrochemical performance. On the other hand, PANI can prevent the self-aggregation of G, rGO, or GO sheets, which would decrease the electrochemical performance. In relation to the combination of PANI with rGO or GO, structures such as PANI nanofibers can be found in rGO, PANI/rGO or PANI/GO gels, PANI/rGO nanoparticles or porous 3D nanostructures, which may lead to additional modifications, for improve their electrochemical performances. In general, rGO is preferred due to fewer functional groups, which increases electrical conductivity and optimizes alignment between nanosheets.

Significant performances have been obtained in electrodes made up of PANI nanofibers or nw on the surface of rGO sheets, using different optimization strategies. In a study by Ock et al. [258], the manufacture of an aqueous asymmetric supercapacitor was reported, with an anode of PANI nanofibers obtained by in situ polymerization, forming networks on rGO nanosheets and a cathode of NiO nanoparticles on rGO. In addition to the larger voltage window that allows an asymmetric supercapacitor, the device was based on an aqueous KOH electrolyte, demonstrating a long service life, with retention of up to ~100% in specific capacitance after 100,000 charge/discharge cycles. Very remarkable was the optimization in the amount of NIBP since the specific capacitance obtained (445.7 F g^−1^ maximum) was dependent on its quantity, morphology, and obstruction (or not) of the spaces available for the insertion/disinsertion of ions. In a different perspective, but where the amount of PANI nanofibers obtained by chemical means was also important, we find the work of Jin et al. [209], where the functionalization of rGO with S was chosen, improving porosity and permeability to the electrolyte. Although the pseudocapacitive mechanism of charge storage granted by PANI was significant, the electrochemical performance was optimized with the functionalization and reduction in GO, increasing the response obtained by CV and decreasing the electrical and ionic resistances, according to Nyquist diagrams.

The achievement of current collectors with abundant porosity has also been shown as an approach to improve the performance of the electrodes in nanocomposites of the PANI/rGO type, as Hong et al. [210] show by manufacturing a ternary carbon fiber (CF) nanocomposite, wrapped in rGO sheets and PANI nanofibers. The CF frame, forming a macroporous 3D structure and functioning as a current collector, allowed independent electrodes to be achieved without the need for another support, with good mechanical stability to support rGO and NIBP and increase the surface area for ion insertion/disinsertion. In this way, not only was the amount of active redox sites offered by PANI optimized but also structural stability reflected in the retention of 94.1% in capacitance was achieved after 2000 cycles for CF@rGO/PANI electrodes. Of importance was the concentration of aniline used to form PANI, obtaining the best capacitance levels for 0.5 M, above the rest of the prepared composites.

Another type of auxiliary modification, at the current collector level, corresponds to the incorporation of a flexible material, such as stainless-steel fabric (SSF), as shown by Yu et al. [211]. The electrosynthesis of rGO and PANI on SSF was reported, forming a nanocomposite with a sandwich-like structure (GP@SSF). The three materials combined presented high porosity, lower electrical resistance, and a greater number of active sites available. Furthermore, it was possible to deposit PANI nw not only on the surface of the rGO nw but also between their channels. The electrode eventually reached an area capacitance of up to 4760.6 mF cm^−2^. When examining a flexible, solid-state symmetric supercapacitor with H_2_SO_4_/PVA gel electrolyte, the actual capacitance of up to 1506.6 mF cm^−2^ (at 6 mA cm^−2^) was obtained, with retention of 92% capacitance, after 5000 charge/discharge cycles. Furthermore, when subjected to 1000 bending cycles of 0–180°, the capacitance was retained at 95.8%. In this, the high porosity facilitating ionic diffusion, the pseudocapacitance of PANI, the sandwich-type structure, which gave good mechanical stability, and the SSF current collector have been fundamental. 

Obtaining PANI/rGO or PANI/GO gels, forming a porous 3D network, allows the design of flexible electrodes with stable electrochemical performance. By means of a two-stage self-assembly method, PANI@rGO nanosheets were obtained, forming a 3D network of colloidal gels, with high porosity, as reported by Wu et al. [212]. Uniformly, and even at the molecular level, PANI coatings were achieved around the rGO nanosheets. The nanocomposite showed a specific capacitance of up to 824 F g^−1^, with a speed capacity of 98% retention, when increasing the current density 24 times. The high speed demonstrated is based on the optimized morphology of the PANI-on-rGO uniform layered gels, which would have formed loosely obstructed channels for rapid ionic diffusion. Likewise, Li et al. has reported a PANI/rGO hydrogel, in the form of flexible fibers [213], by self-assembly of PANI and GO hydrogels and subsequent reduction. Although PANI 3D networks can show poor mechanical properties, in combination with rGO, a flexible hydrogel has been achieved, where electrostatic interactions, p-p type, and hydrogen bonds, between the precursors, GO, PA, and PANI, were fundamental. The inclusion of PANI between rGO nanosheets was essential to avoid their self-aggregation, which would decrease porosity, flexibility, and electrochemical performance. By integrating the PANI/rGO hydrogel electrodes, with a H_2_SO_4_/PVA gel electrolyte, a fiber-shaped device was obtained, delivering 112 F g^−1^ and 8.80 mWh cm^−3^ of specific capacitance and volumetric energy density, respectively. Notably, under 1000 draw cycles, the capacitance was retained at ~100%.

An illustrative study of the influence that an extra component may have on the interactions of PANI and rGO in hydrogels and, ultimately, on the performance of an electrode corresponds to that carried out by Zou et al. [214], who reported the synthesis of hydrogels denoted as GMPH7, composed of rGO/PANI and *m*-phenylenediamine. The latter destabilized the interactions between the residual functional groups in GO and the PANI amines, causing better maintenance of the conjugated structure of the PANI chains, which had an impact on the specific capacitance levels, increasing from 375.3 F g^−1^ to 514.3 F g^−1^. The symmetric GMPH7 supercapacitors had excellent stability under 2000 bending cycles at 135°, which was due to a pseudocapacitance activation process due to the intercalation and rearrangement of PANI between the rGO sheets.

As demonstrated by Xiong et al. [215], it is even feasible to obtain micro-supercapacitors, based on GO/PANI gels, with dynamic 3D networks, by means of a spray printing technique on PET and poly(imide)/Au substrates. By controlling the rheological properties of the gel, they achieved a material capable of rapid recovery after being subjected to constant high-speed stresses, which is important for spray printing manufacturing and for obtaining flexible devices. Compared to other electrodes designed with printing techniques [259,260,261], a higher area capacitance was obtained, reaching 44 mF cm^−2^ at a current density of 0.1 mA cm^−2^. According to what was mentioned by the authors, the structural ordering granted by GO on the PANI nanofibers would have been essential both mechanically and electrochemically [215]. 

In a design closer to the arrangement of nanoparticles or nanorods, there are studies such as that of Tabrizi et al. [216], where PANI was synthesized by in situ chemical polymerization in the presence of GO in an acid medium. Here the influence of the acid used as a dopant was investigated, both on the structure and on the electrochemical performance of the nanocomposite. With respect to HCl, HClO_4,_ and PTSA, the highest specific capacitance was obtained when using H_2_SO_4_, reaching a maximum of 727 F g^−1^ for an electrode and 447 F g^−1^ for a complete symmetric cell. Likewise, retention of 95.7% at 5000 cycles and the speed capacity can be highlighted by increasing the current density 20 times. This would have been related to its abundant microporosity, obtaining the highest surface area (51.43 m^2^ g^−1^), with respect to the rest of the acids, in addition to the abundant nanoparticle/nanorod nanoarrays of PANI on GO, significantly optimizing space for electrolyte insertion. 

To obtain flexible and independent electrodes, Hu et al. [217] demonstrated the chemical preparation of PANI nanoparticles on rGO, assisted by tannic acid. The latter acted as a binding agent for aniline monomers to facilitate the formation of nanoparticles. Although no notable mechanical stability and speed capacity were obtained, stability in charge storage was verified when subjected to bending cycles. Although it would require further optimization to improve some parameters, it is an investigation that proves to obtain PANI/rGO electrodes without the need for additives or supports, which allows the stable operation of flexible devices. Similarly, regarding G/PANI-based nanocomposites, there are different types of structures used in supercapacitor electrodes. In order to mention a few cases, it has been possible to synthesize nanorods, nanofibers, or even carbonized PANI, in direct contact with leaves of G. The benefits achieved from the combination of G, nanofibers, and PANI nanorods, as exemplified in work by Li et al. [218]. With the nanocomposite denoted GP-P, there were improvements in mechanical stability, ion mobilization, and electrical and ionic conductivities. On the one hand, the nanofibers formed connecting bridges between sheets of G, enhancing electrical conductivity, and, on the other, the nanorods would have been sandwiched between said sheets, limiting self-aggregation, granting pseudocapacitance and facilitating ionic diffusion. This was reflected in factors such as obtaining a maximum specific capacitance of 578 F g^−1^ with longer charge/discharge times. In turn, when using this electrode in an asymmetric supercapacitor, a retention of 93% in capacitance was achieved after 10,000 charge/discharge cycles. In a case applied to obtaining microsupercapacitors, Wu et al. [219] prepared nanocomposites consisting of G fibers wrapped by PANI nanorods (PNA/G) by means of a microfluidic method, with self-assembly, annealing, and in situ polymerization steps. The central fiber of G, with high electrical conductivity, allowed a good anchoring of the PANI nanorods, presenting remarkable mechanical flexibility when subjected to bending tests and being able to integrate with different gel-H_3_PO_4_/PVA and EMITFSI/PVDF-HFP electrolytes. Compared to the pristine G fiber, the incorporation of PANI nanorods improved the area capacitance, reaching up to 230 mF cm^−2^, with higher energy and power density levels, compared to other microsupercapacitors found in the literature [262,263,264,265,266,267]. Its practical application was tested using parallel connections, powering LED lights, and a digital clock [219]. 

The electrodes composed of PANI nw and G sheets reported in the recent literature can incorporate other types of additional modifications to optimize some properties. For example, Khosrozadeh et al. [220] designed flexible electrodes made up of PVA nanofibers covered with G sheets, on which PANI nw were deposited through in situ chemical polymerization, which, in turn, were covered with a second layer of G. Inclusion of PVA improves electrolyte permeability, surface area and gives anchoring stability, both to G and PANI. The first layer of G would also have been significant in fixing the nw to avoid their rapid degradation in charge/discharge processes. Additionally, the incorporation of the second layer of G improved the electrical conductivity, speed capacity, specific capacitance, and very long-term cycle stability, reaching a retention of 75.6% after 88,000 cycles, demonstrating an important pseudocapacitive activity in the cyclic voltammograms. A different approach within the same scope is reported by Feng et al. [221], where the nanocomposite has been called GNC. This exhibits a hierarchically porous structure, increasing the surface area and allowing greater storage of charge through double electrical layers and mesopores, which allows easy and fast mobility of ions at higher current densities. The incorporation of EMIMBF_4_/PVDF-HEP (support electrolyte) allows observing retentions of 88.3% of the capacitance after 1000 bending cycles and feeding up to 30 LED lights, with a voltage of 3.5 V. According to a study by Jin et al. [222], the incorporation of some flexible substrate has proven to be viable when integrating with a nanocomposite made up of G/nw-PANI. A core composed of cotton fibers was used, obtaining supercapacitors in the form of fibers with good operational stability under bending. Good adhesion between the different components was demonstrated by evaluating their stability after 3800 charge/discharge cycles, retaining 98% of their area capacitance. Related to charge storage, it is worth highlighting the role of the PANI nw arrangement, which optimized the surface area and the number of active sites available, with better performance obtained from their synergistic combination.

Among other types of G/PANI structures, an investigation carried out by Mangisetti et al. [223], where an electrode with a hierarchically porous 3D nanoarchitecture was manufactured, composed of porous carbon, nanosheets of G and chemically deposited PANI. Something important that emerges from this study is that the structure with the highest surface area and pore volume is not necessarily the one with the best specific capacitance. In fact, for a nanocomposite with 5% by mass of PANI, the best values of surface area (1201 m^2^ g^−1^) and total pore volume (2.14 cm^3^ g^−1^) were obtained, but their specific capacitance levels were lower than 15% NIBP (1198 F g^−1^), reaching a maximum of only 720 F g^−1^. With regard to the latter, the importance of the incorporated NIBP mass percentage emerges. Under this arrangement of supercapacitors (3D PC-g/ PANI-15), an energy density of up to 97.5 Wh kg^−1^ was achieved, which can be applied to power LED lights. 

Another 3D nanoarchitecture has been reported by Zheng et al. [224] regarding the optimization of active sites for cargo storage. A multi-site formation strategy was chosen for the growth of PANI nanoarrays on G leaves (MSG/PANI). In this work, it was possible to increase the amount of oxygen-containing functional groups in the leaves of G, increasing the number of growth sites of PANI nanoparticles and achieving a capacitance of up to 912 F g^−1^, with a retention of 86.4% at an increase up to twenty times the current density. In this study, not only were abundant active sites and optimized porosity for ionic diffusion obtained, but it was also possible to manufacture flexible supercapacitors with stable operation.

The use of CNTs also makes it possible to obtain composites of high porosity and surface area for easy ion transport, they can act as structural support for PANI nanostructures in the face of charge/discharge processes, it provides better electrical conductivity, it makes it possible to obtain flexible electrodes and provides charge storage through the double electrical layer. As an example, Liu et al. [186] presented the manufacture of electrodes with a core-shell type structure based on CNT fibers (CNF and a nanostructured layer of electrosynthesized PANI). With such electrodes, flexible solid-state supercapacitors were manufactured with stable operation under bending cycles. Due to the formation of porous 3D networks of CNTs and nanostructured PANI, good performances were obtained, in terms of speed capacity and cycle stability, with an area capacitance greater than their separate components. A similar ternary nanocomposite can be found in Agyemang et al. [226], although in this case, carbon nanofibers (CNFs) covered by CNTs and a subsequent layer of PANI in the form of nanofilms, synthesized by chemical means in situ, were used. For this arrangement, the PANI nanostructured layer was the main contributor to the electrode capacitance, measuring values up to 1119 F g^−1^. This study shows that is not only porosity at the nanoscale is important, but also macroporosity on the micrometric scale, offering wide channels for rapid and abundant ionic diffusion with less impeded trajectories. CNT/PANI can also be integrated into flexible electrodes with self-healing properties, as demonstrated by Guo et al. [227]: by in situ chemical polymerization, PANI nanoparticles were synthesized, to which networks of SWCNTs were added, which were deposited on a flexible and self-repairing PVA-H_2_SO_4_ hydrogel substrate. The symmetric supercapacitor reached an area capacitance of up to 15.8 mF cm^−2^, being better than only NIBP (3.85 mF cm^−2^) in correspondence with its GCD curves. This was probably attributable to their combination producing a morphology with higher available redox active sites and more spaces for ion penetration. In turn, it presented a self-repair efficiency of 80%, after five cutting and repair cycles.

According to some recent reports, G sheets can also be incorporated into the mixture of PANI and CNT nanostructures. In the work of Lu et al. [228], it was demonstrated to obtain highly flexible supercapacitors, with up to 800% of maximum deformation, constituted by CNT/G/PANI electrodes in the form of fiber. On the one hand, the incorporation of G sheets generated greater porosity when the CNTs beams were intercalated and separated, to the detriment of the electrical conductivity and, on the other hand, the electrosynthesized PANI nanolayer increased the specific capacitance of the nanocomposite (up to 182.6 F g^−1^). These supercapacitors were able to integrate with silicone-covered textile fibers and demonstrated good cycle stability under different stretching conditions. Another type of nanoarchitecture with the same components is reported by Liu et al. [229], who synthesized sandwich-type electrodes formed from PANI nanorods interspersed between G sheets and networks of CNTs, due to p-p-type interactions. The achievement of a high surface area has been remarkable for the easy and rapid entry of ions towards the active sites of PANI, G, and CNTs, as well as the mechanical stability provided by the carbonaceous materials in the PANI nanorods. This was reflected in the maximum specific capacitance of 638 F g^−1^ at 0.5 A g^−1^ (414 F g^−1^ for PANI/G), with retention of 88.2% at 10 A g^−1^ and 93%, for 2000 cycles. Although it demonstrated good stability, it is questionable that the performance of the material has not been studied in a long period (over 10,000 cycles).

In order to improve the electrical conductivity and structural integrity of materials based on metal oxides, some research has resorted to incorporating PANI for hybrid nanostructures. Of course, both metal oxides and PANI chains provide active sites to store charge through redox reactions. For example, Ma et al. [232] showed the manufacture of an electrode with core-shell type 3D morphology based on coaxial networks of Fe_3_O_4_ microspheres covered by chemically obtained PANI nanofibers. In turn, these nanofibers interconnected the microspheres, forming chains and nano-networks. The chain microsphere structure would act as a mechanical stabilizer before the charge/discharge processes, while the PANI nanofibers would be the main contributors of pseudocapacitance. PANI coatings on the surface of the microspheres would have been facilitated by the hydrogen bonds formed between the—NH groups of PANI and the O atoms of Fe_3_O_4_, reaching a maximum capacitance of 620 F g^−1^. 

For use in asymmetric supercapacitor anodes, Yang et al. [268] prepared PANI hydrogels chemically, covering and connecting a-Fe_2_O_3_ nanorods. In this way, a hierarchically porous material was obtained, with a 3D morphology of abundant interconnected nanofibers, with better electrochemical performance, compared to its separate components. The PANI hydrogel would have provided the optimization of different properties, namely: it functioned as a kind of conductive binder without the need for more additives; allowed rapid electrical conductivity through the highly interconnected 3D network; its hydrophilic character through the—NH groups and its porous structure, would have facilitated ionic diffusion to the active sites, through more reduced trajectories; provided mechanical stability against charge/discharge cycles and allowed the storage of charge by pseudocapacitance, even at negative potentials (vs. SCE) [23]. This electrode managed to retain 98.2% of the specific capacitance after 5000 charge/discharge cycles.

In a reverse approach, instead of obtaining a core based on a metal oxide wrapped in PANI, Yang et al. [234] designed a nanocomposite with a PANI core covered by MnO_2_ in order to directly expose this inorganic material to the electrolyte. On a carbon fiber fabric substrate, they chemically synthesized a porous 3D nanostructure of PANI coated with MnO_2_ nanoflakes. This electrode achieved a retention of 98.5% capacitance after 10,000 charge/discharge cycles. The synergy involved avoided the rapid degradation of the materials, relieving the stresses produced by volumetric changes. A disadvantage of the nanocomposite was the lower speed capacity, related to higher electrical and charge transfer resistances. This would have been produced by the greater amount of MnO_2_ present in the PANI@MnO_2_ nanocomposite compared to the electrode with only MnO_2_ nano blades. 

Regarding bimetallic oxides, Ghadimi et al. [235] synthesized PANI nanofibers by in situ chemical polymerization, on whose surface MnFe_2_O_4_ nanoparticles were deposited, forming a nanocomposite, which underwent a final annealing step. Consequently, the PANI nanofibers were carbonized, obtaining a carbonaceous material doped with N (NC) and decorated with nanoparticles. Both MnFe_2_O_4_ and the N atoms of the nanofibers were contributors to the pseudocapacitance obtained, while the carbonaceous structures allowed the storage of charge via a double electrical layer. Other functions that the N atoms would fulfill would be the fixation of the nanoparticles through interactions with the O atoms in MnFe_2_O_4_ and the increase in the permeability for the insertion of ions towards the redox-active sites. Although at a current density of 1 A g^−1^, the non-carbonized PANI/MnFe_2_O_4_ nanofiber-based electrodes showed higher specific capacitance, at higher current densities, the carbonized nanocomposites were superior. The good stability of the nanocomposite was demonstrated by evaluating a symmetric supercapacitor, obtaining a retention of 97% after 10,000 charge/discharge cycles [235]. 

The combination of some metallic sulfide with a CP has been a common strategy to optimize electrode performance, and, of course, PANI has been tested on these nanocomposites. For example, Zhang et al. [236], with the assistance of templates, synthesized hollow MoS_2_ microspheres covered with a PANI nanolayer with a porous structure obtained by in situ chemical polymerization. It was found that for a 1:2 mass ratio of MoS_2_:PANI, the best electrochemical performance was achieved. Regarding the capacitance obtained, in addition to the pseudocapacitance of PANI, the hollow structure of the microspheres would have allowed better ionic access to the active sites of MoS_2_, storing charge by the pseudocapacitive mechanism of intercalation—more common in battery electrodes. The nanocomposites demonstrated remarkable speed capability, retaining 72% of the specific capacitance at 20 A g^−1^ and good long-term cycle stability, but with a rapid decay within the first 400 cycles. Therefore, degradation of the active materials could occur, both within the MoS_2_ microspheres and in the PANI coatings, during the insertion/disinsertion of ions. 

In the synthesis of 2D nanostructures, the incorporation of PANI in an MXeno of Ti_3_C_2_Xx has been shown to improve the results in parameters such as capacitance, cycle stability, and speed capacity. According to Vahid Mohammadi et al. [237], the PANI chains were obtained by oxidant-free in situ chemical polymerization on the surfaces of the nanofilms. The OH groups and O atoms present in Ti_3_C_2_X_x_ were proposed to be the oxidizing agents, with successive oxidations of dimers and oligomers. Regarding specific capacitance, the highest levels were obtained for 4 µm M/P type electrodes, and even for a 13 µm one, its specific capacitance at 20 mV s^−1^ was 13% and 47% higher than M type electrodes 3.5 and 10 µm, respectively. On the other hand, the highest levels of area capacitance were for 90 µm M/P type electrodes since, for the same electrode area, there is a higher active mass to store charge. For 4 µm M/P type electrodes, specific and volumetric capacitances of up to 412 F g^−1^ and 1353 F cm^−3^ were reached, respectively, with an excellent retention of 98.3% after 10,000 cycles. Additionally, the use of MXeno made it possible to obtain independent electrodes, without the need for other additives, to manufacture symmetrical supercapacitors with energy densities up to 79.8 Wh L^−1^.

In the setting of NIBP combined with rGO and some metal oxide, there are several reports to consider [236,238,239,240,241]. Viswanathan and Shetty [238] prepared a ternary nanocomposite of rGO, Cu_2_O-CuO, and PANI by means of a one-stage chemical method, and the influence on the electrochemical performance was studied, according to the variation in the relative quantity of the different components. In this case, the best charge storage was for a nanocomposite of rGO, Cu_2_O-CuO, and PANI, with masses of 75, 250, and 300 mg, respectively, denoted as G12CP. In this way, a maximum specific capacitance of 684.93 F g^−1^ was reached, integrating the pseudocapacitance of PANI and Cu_2_O-CuO and the storage by an electric double layer of rGO. This article demonstrates how essential the balance is between the quantity of active material, optimization of spaces, and effective availability of cargo storage sites. 

To be used in symmetric supercapacitors and as cathodes in asymmetric supercapacitors, Ghosh and Yue [239] designed sandwich-type nanocomposites made of PANI/MnO_2_-rGO/PANI. The enclosed layers of MnO_2_-rGO were synthesized by a method that involved vacuum filtration and hydrothermal reduction, showing a porous 3D structure with MnO_2_ in the form of nanoparticles. This oxide, in addition to preventing the auto aggregation of rGO, would have provided pseudocapacitive active sites. Symmetric supercapacitors delivered a capacitance of up to 148 F g^−1^ (at 1 A g^−1^), with a good retention of 76.5% at 5 A g^−1^. On the other hand, asymmetric solid-state devices with G/MoO_3_ airgel anodes demonstrated stable performance when flexed under different angles, reached a capacitance of 146 F g^−1^ and an energy density up to 541.91 Wh kg^−1^, testing on 1.8 V green LED lights.

The use of PANI: PSS complexes—more usual with PEDOT—in combination with inorganic and carbonaceous materials has been reported by Fenoy et al. [240]. Using a layer-by-layer assembly process, they synthesized nanocomposites composed of PANI:PSS complexes and graphene modified with Fe_3_O_4_ nanoparticles (5–10 nm in diameter). Specifically, layers of PANI:PSS and G/Fe_3_O_4_ were alternated, obtaining electrodes of 3, 9, 15, and 21 bilayers. The highest capacitance achieved was for 15 bilayer nanocomposites, reaching 768.6 and 659.2 F g^−1^ (at 1 A g^−1^) in electrolytes of HCl and KCl, respectively. The advantage that a layer-by-layer assembly can offer, in terms of electrochemical performance, compared to an electrode based on a direct mixture of PANI:PSS with G/Fe_3_O_4_ was also evaluated. In this regard, for the 15-bilayer nanocomposite, the capacitance was approximately 20 times higher, validating the proposed nanostructuring strategy. In the structures assembled layer by layer, there is a risk of self-aggregation and blocking of channels for the insertion of ions. However, according to the results presented, the above would not have happened, given the optimization in capacitance when adding up to 15 bilayers in the electrodes. On the other hand, considering the cycle stability, with retention of 84% after 1600 cycles, the nanocomposite would require further optimization, and it is not clear which component would have been the most degraded or if there was any structural modification that could increase the electrical resistance of the material. 

The nanostructures of perovskites with oxygen vacancies have demonstrated a pseudocapacitive activity that can be enhanced in combination with other materials, as demonstrated by Shafi et al. [241]. In this study, they have designed electrodes based on a ternary composition made up of LaMnO_3_ nanoparticles deposited on rGO sheets, and interconnected with PANI nanofibers obtained by in situ chemical polymerization. Compared with the separate components, the LaMnO_3_/rGO/PANI nanocomposite showed, in general, better electrochemical performances. Along with obtaining a morphology of abundant porosity, the PANI nanofibers and the rGO sheets would have enhanced the electrical conductivity and the mechanical stability of the electrodes surrounding the LaMnO_3_ nanoparticles. The structural optimization was reflected in the remarkable cycle stability exhibited by an asymmetric supercapacitor, one of whose electrodes were made up of the nanocomposite, reaching a retention of 116% of the initial capacitance after 100,000 charge/discharge cycles. At the level of this bibliographic review, it is one of the highest cycle stabilities found.

To enhance the capacitance, electrical conductivity, and surface area of bimetallic oxides, in addition to nanostructuring, the synergistic combination with PANI and some carbonaceous material has been used, as Shen et al. [242]. Electrodes composed of nw with a NiCo_2_O_4_@NiMoO_4_ core-shell structure were manufactured on carbon fabric fibers, with a final stage in which PANI nanorods were chemically deposited on the surface of the nw. This electrode allowed retention of 63.4% of the initial capacitance by increasing the current density ten times, higher than NiCo_2_O_4_/CC (18.9%) and NiCo_2_O_4_@NiMoO_4_/CC (51.4%). The addition of pseudocapacitance from PANI allowed to increase in the maximum capacitance from 887.5 F g^−1^ to 1322.2 F g^−1^ and decreased the RCT to 2.0 Ω, reflected in the Nyquist diagrams.

Regarding the incorporation of noble metals, to take advantage of the electrical conductivity that an Ag nw arrangement can provide, Chen et al. [243] added carbon layers 20–30 nm thick to allow the subsequent growth of PANI nw by a dilute chemical polymerization method, obtaining a core-shell type nanostructure. The deposit of this nanostructured polymer would be the main contributor with active redox sites in a wide surface area. On the other hand, the carbon layer would have allowed the continuity in the mobility of electrons to and from the Ag nw and functioned as a mechanical stabilizer for PANI. Compared with electrodes from PANI, PANI@C, and nw-Ag/PANI, the nanocomposite nw-Ag@C@PANI showed the highest maximum specific capacity (785 C g^−1^), the lowest ionic strengths (0.52 Ω) and charge transfer (9.87 Ω) and best capacity retention after 3000 charge/discharge cycles (94.1%).

In order to improve some structural or electrochemical properties in supercapacitor electrodes, there are strategies based on combining PANI with materials from biomass [247,248,249,250,251,252]. For example, taking advantage of the advantages offered by hydrogels, a recent work by Han et al. [247] in which they designed independent electrodes of this type with self-healing or repairable properties. On cellulose nanofibers as substrates-molds, they synthesized PANI by oxidative chemical means, obtaining conductive nanocomplexes denoted as CNF@PANI, which were homogeneously deposited on the interconnected fibers of a PVA-borax hydrogel. A parameter related to the mechanical properties of the hydrogel was its maximum compression stress, reaching a value of ~48.8 kPa, 3.5 times higher than that obtained for a PVA gel. The designed electrodes showed an electrical conductivity of up to 5.2 ± 0.07 S m^−1^, a medium-low level of specific capacitance (226.1 F g^−1^ max.), A good retention of 80.7% when increasing the current density 10 times, although with only 74% retention after 5000 charge/discharge cycles. These arrangements made it possible to achieve other properties such as elasticity, biocompatibility, and self-repair with the recovery of operability. In this way, they proposed this promising type of electrode for applications in biomedicine. 

Another case of synthesis of PANI-natural fibers is that in which an inexpensive, lightweight, and flexible cellulose fiber thin film electrode, based on PANI nano-dendrites@fiber, is reported. These electrodes demonstrate an excellent specific capacitance of 296 Fg^−1^ to 1 Ag^−1^ and an aerial capacitance of 5017 mF cm^−2^ at 10 mA cm^−2^. With these electrodes, flexible micro-supercapacitors were fabricated using PANI@ paper, reaching specific capacitance up to 282 Fg^−1^ to 1 Ag^−1^, and maximum energy density of 2.5 Wh cm^−2^, demonstrating superior performance to other reported micro-supercapacitors [268,269,270]. A different way of treating materials derived from biomass can be found in the work of Lyu et al. [249], using N-doped carbonaceous microspheres prepared from yeast (Saccharomyces cerevisiae). On these structures, PANI nw were deposited by in situ chemical polymerization, interacting with the N atoms of the microspheres by means of hydrogen bonds. According to what was reported, the microspheres presented high porosity, with a surface area of 269.18 m^2^ g^−1^, which later decreased to 24.0 m^2^ g^−1^, due to the insertion of the nw. However, this loss in surface area could be compensated with the pseudocapacitance offered by PANI in relatively ordered arrays of nw. Compared to microspheres without PANI, with a maximum capacitance of 234.5 F g^−1^, composites synthesized with an optimized amount of PANI showed a capacitance of up to 500 F g^−1^ (at 1 A g^−1^). Regarding speed capacity and cycle stability, it showed good yields, with a retention of 77% for 20 A g^−1^ and 91.8% after 5000 cycles [249]. Thus, the carbonaceous microspheres optimally supported the PANI nw, avoiding rapid degradation and, in addition, acted as supports of significant electrical conductivity.

The mixture of more traditional carbonaceous materials with biomass derivatives and PANI has also been used recently for the manufacture of electrodes. Wang et al. [250] used PLA, which is a biodegradable aliphatic polyester derived from corn or wheat, as a substrate on which MWCNTs were deposited, on whose surfaces PANI nanoparticles were chemically synthesized. The nanocomposites denoted as P-PCP-3-H showed a morphology of abundant porosity, obtaining the best electrical conductivity (0.25 S cm^−1^) and a maximum capacitance of 458.1 F g^−1^ for 15.74% by mass of PANI and with heat treatment at 60 °C for 1 h. This last process allowed a rearrangement of the PLA chains and a phase separation between them and the CNTs. Therefore, electrodes with a capacitance of up to 510.3 F g^−1^ (at 1 A g^−1^) were obtained, with better speed capacity, long-term stability with retention of 111.5% (2000 cycles), and greater retention under cycles of bending at 180° (93.4%), relative to electrodes without heat treatment. Tahir et al. [271] showed PANI-coated CoRu-LDH (CoRu-LDH/PANI) nanowires as supercapacitors. Based on a 6 M KOH aqueous electrolyte, the SnO_2_/MWCNTs/PANI as an electrode material displays a capacity of 211 Cg^−1^ at 1 Ag^−1^ and exhibits excellent cycling stability with a capacitance retention ratio of 98% after 5000 cycles at a current density of 2 Ag^−1^. In another study in the same area, Choudhary et al. [272] also show the inclusion of carbonaceous materials into the polymeric matrix. They show that the presence of CuS improved the redox activity and charge storage capability of the polymer-derived composites. The PPy-rGO-CuS composite with 10 % rGO and 5 % CuS showed a specific capacitance of 237.7 Fg^−1^ at 1 Ag^−1^, constant capacitance retention up to 2000 galvanostatic charge-discharge (GCD) cycles, and an energy density of 21.1 Wh kg^−1^ at 1 Ag^−1^. Furthermore, Wang et al. [251] carbonized kenaf stems to form a 3D macroporous carbon structure, onto which they electrodeposited rGO and what they describe as vertical nanoarrays of PANI. The incorporation of rGO allowed increasing in the capacitance levels from 337.5–454.8 F g^−1^ to 626.8–1224 F g^−1^, reaching an energy density of 144.4 Wh kg^−1^, being superior performances with respect to other reports of electrodes with these materials [273,274,275,276,277,278,279]. In this optimization, in addition to the electric double-layer mechanism to store charge and the increase in conductivity granted by rGO, the increase in the number of NIBP nano-arrays was key, increasing the amount of redox-active sites.

Another alternative has been the incorporation of inorganic materials in matrices derived from biomass and PANI, as shown by a study by Wu et al. [252], where they designed highly flexible electrodes from a 3D network of lyophilized bacterial cellulose (BC), used as a substrate and template for the deposit of PANI nanolayers chemically synthesized in situ and a final inorganic layer of LDH from NiCo in the form of nanofilms. These different nanostructures coexisting in the same nanocomposite, with hierarchical morphology, allowed optimization of the surface area with active sites and abundant channels for ionic diffusion. PANI nanolayers not only served as pseudocapacitive material and pathways for electronic transport but also as a support for the anchoring of NiCo-LDH nanofilms. The electrodes reached specific capacitance levels of 1690 and 778 F g^−1^, for 1 and 15 A g^−1^, respectively, with a retention of 83.2% after 5000 cycles, being much higher in these parameters, with respect to NiCo-LDH/BC only electrodes. The 3D structure of bacterial cellulose was what allowed the flexibility of the electrodes, despite the decrease in elongation at break when adding PANI and LDH (from 11.8 to 7.2%), demonstrating a retention of the capacitance of 91.4% after 3000 cycles of flexion. In addition, these electrodes were used in asymmetric solid-state supercapacitors, with an energy density of up to 47.3 Wh kg^−1^, achieving a higher level compared to other asymmetric devices of the state-of-the-art, with purely inorganic cathodes based on sulfides, oxides or LDHs of Ni and Co [280,281,282,283,284,285]. Recent research, such as that of Hassan et al. [286], also showed the synthesis of a zinc-strontium sulfide-based supercapacitor device with PPy-doped activated carbon. In an asymmetric supercapacitor, these compounds showed that the device (ZnSrS//PANI@AC) had a maximum capacity of 148 C g^−1^ and an energy density of 32.88 Wh/kg at the power density of 800 W kg^−1^ and after the completion of 5000 cycles, ZnSrS//PANI@AC retained 90% of its initial capacity, demonstrating that this nanostructured composite can be a novel material, which could be used in energy storage devices.

Next, we will address PANI nanostructures evaluated as supercapacitor electrodes, which at most contain some modifying agent at the atomic level, a dopant, or another secondary additive [253,254,255,256,257]. Starting with one of the recently used successful approaches, a PANI hydrogel synthesized by Zhou et al. [253], by in situ chemical polymerization, using V_2_O_5_⋅nH_2_O nw as an oxidizing agent and removable template. After optimizing the preparation, an electrical conductivity of 0.12 S cm^−1^ and an elastic modulus of 3.1·10^5^ Pa were obtained. The electrodes showed a maximum capacitance of 636 F g^−1^ (1.54 F cm^−2^) at 2.0 A g^−1^, losing only 1.6% when increasing to 25 A g^−1^ and with retention of 83.3% after 10,000 charge/discharge cycles, parameters that were higher compared to PANI bulk films. 

The manufacture of flexible electrodes based on N-doped carbon derived from carbonized PANI can be seen in the work of Zhao et al. [255], who synthesized arrays of PANI nw electrochemically on carbon paper, subjecting them to a final calcination stage at three different temperatures (400, 600 and 850 °C). The NC nw with defects in their structure presented specific capacitance of up to 404 F g^−1^, with retentions of 78% and 98.1%, when the current density increased 10 times and after 5000 cycles, respectively. This meant better yields, both in comparison with the rest of the nw carbonized at the other temperatures, as well as with the nw without carbonizing [255]. In a similar work, Zhao and Xie [256] synthesized B, N-C nw of ~90 nm in diameter, forming hierarchically porous interconnected networks with a surface area of 1022.4 m^2^ g^−1^, which increased electron mobility in the p conjugated system and created Faradaic activity promoter defects. In addition to the maximum capacitance of 504 F g^−1^ (at 1 A g^−1^), its retention of 66.9% when reaching 10 A g^−1^ and 97.4% after 10,000 cycles can be highlighted [287,288,289]. 

Another type of modification that can be added to carbonaceous structures derived from PANI corresponds to the incorporation of Fe cations, as did Rantho et al. [257]. In this case, relatively agglomerated nanograins of NC derived from carbonized PANI were obtained, with cations of Fe^2+^ and Fe^3+^ present in the carbonaceous structure, in configurations of carbides, sulfides, and nitrides, according to XPS analysis. When evaluating the electrochemical behavior of the electrodes in an aqueous KOH electrolyte, it was observed that at negative potentials (−1.2 to 0.0 V vs. Ag|AgCl), the capacitive mechanism of the double electrical layer was predominant, while at positive potentials (0.0 at 0.45 V vs. Ag|AgCl), a pseudocapacitive behavior appeared, with redox activity in the Fe centers. This allowed cathodes with better capacitance levels compared to the anodes. The manufactured supercapacitors delivered a maximum energy density of 41.3 Wh kg^−1^, with retention of the capacitance of 72% after 10,000 cycles.

### 7.2. Ppy

With regard now to PPy nanostructures, they have been classified into the following categories: combination with carbonaceous materials (Table 3, entries 01–15); combination with inorganic materials (Table 3, entries 16–22); in hybrids with carbonaceous and inorganic materials (Table 3, entries 23–30) and other nanostructures (Table 3, entries 31–42). The most outstanding works are reviewed in detail below.

As in the case of PANI, there is extensive literature about PPy with materials such as rGO, GO, or CNTs. However, it will be limited to more recent reports in the area [292,293,294,295,296,297,298,299]. Within the scope of electrodes composed of PPy/rGO, there is a study by Zhu and Xu [[292]], where the optimization of the PPy/rGO nanosheets was investigated, finding that for 10% by mass of rGO, the greater surface area (124 m^2^ g^−1^; 26 m^2^ g^−1^ for PPy), with mesopores of 2–5 nm in diameter. Although more quantity of rGO increased the electrical conductivity, such improvement did not correlate with an increase in capacitance since, on the other hand, the surface area with active sites decreased. In addition, this morphological optimization could be translated into a good performance in speed capacity, retaining 85.2% of the capacitance when increasing the current density from 0.2 to 12.8 A g^−1^, explained by a remarkable facility for the ions to quickly reach the active sites of the nanocomposite. Along with the above, we can highlight the extraordinary long-term stability, well above PPy bulk electrodes, retaining 97% after 20,000 cycles, associated with the incorporation of rGO and its good PPy anchoring. 

The use of PPy on flexible substrates of textile type or in the form of threads is a good strategy for obtaining flexible supercapacitors integrated into electronic devices. Li et al. [293] demonstrated the design of two types of electrodes, one of the woven types and the other in the form of a wire. On polyester fibers modified with SnCl_2_ (M-PEF), rGO was deposited by means of a painting and drying strategy. Then, a layer of PPy was deposited on this structure by in situ chemical polymerization. The 1D fibers served as wire-type electrodes, and when crossed, a 2D fabric-type structure was obtained (these electrodes were represented as PPy/rGO/M-PEFT and PPy/rGO/M-PEFY, respectively). In addition to the storage of charge by rGO, the PPy nanolayer allowed an increase in said parameter through its pseudocapacitance and, having an optimization of the surface area, a better display of its electrochemically active sites was obtained when comparing the capacitance with respect to other electrodes. Textiles, for example, are based on CNTs/cotton [317], ZnO/activated cotton [318], MnO_2_/CC [319], PPy/CC, and polyester/AC [320]. On the other hand, the importance of the synergy between rGO and PPy was reflected when comparing the performances in capacitance, cycle stability, and speed capacity with respect to electrodes without one of the two components. In the evaluation of the symmetric supercapacitors based on PPy/rGO/M-PEFT and PPy/rGO/M-PEFY, after 1000 bending cycles, 0–180° retention in capacitance of 97.6% and ~100% were obtained.

Along the same lines, Barakzehi et al. [292] showed the manufacture of the PET/rGO/PPy nanocomposite through GO reduction steps and in situ chemical polymerization. After optimizing the material, a combination of rGO nanosheets surrounded and intercalated by PPy nanoparticles was obtained, with diameters from 43 to 260 nm. The electrical and electrochemical properties depended not only on the PPy nanoparticles but also on the rGO content. Within the range studied, the greater the amount of rGO, there was increase in capacitance and electrical conductivity (up to 0.88 S cm^−1^), reaching an area capacitance of 0.23 F cm^−2^. The contribution of PPy was also relevant in terms of electrical conductivity, increasing it by four orders of magnitude and showing a low RCT at the interface with rGO [294].

In order to avoid rapid degradation of the PPy chains, core-shell type structures can also be chosen, with G envelopes acting as conductors, stabilizers, and charge storage. Although the common thing is to unite G and PPy through p-type interactions, a work by Qi et al. [295], where through covalent bonds, G nanoflakes were attached to PPy nanotubes with internal and external diameters of 80 and 150 nm, respectively. Using G functionalized with carboxyl groups, acylation of the—NH groups of PPy took place, forming bonds of the N-C=O type [295]. The highest specific capacitance was obtained for a G:PPy mass ratio of 1:3, but the best long-term stability was achieved for a ratio of 9:1. After several optimizations, the study opted for electrodes with a G:PPy ratio of 1:3 to manufacture a flexible solid-state symmetric supercapacitor, with stable performance under strain and obtaining 80% retention of capacitance after 5000 cycles [295]. 

The appropriate choice of materials can allow the obtaining of multifunctional devices. A CP such as PPy, which is very versatile in terms of integration with different substances and morphologies, is a powerful candidate for use in these applications. For example, Park et al. [296] reported the manufacture of a dual-functionality device, acting as a solid-state supercapacitor and a pressure/temperature/strain sensor. The microporous nanocomposite was composed of polydimethylsiloxane (PDMS), PPy, and graphene foam (GF) (PDMS/PPy/GF). Focusing on the nanocomposite as a supercapacitor electrode, it is worth highlighting its porous structure and surface area and the p-p-type interactions between G and PPy, fundamental in terms of cycle stability, allowing an attenuation in its long-term degradation, which it would not be possible without their presence [296]. During the first 2000 cycles, there was an increase in capacitance, increasing the availability of active sites with the passage of the cycles. On the other hand, the storage of charge through an electric double layer in graphene was enhanced by the pseudocapacitance of PPy. However, the capacitance levels were at a medium-low level, with a low-speed capacity with increasing current density. The incorporation of PDMS was important to improve the flexibility of the material and the integration of the supercapacitor in a multifunctional device [296].

In addition to the incorporation of G nanofilms, Zhou et al. [292] have used CNTs to form a ternary nanocomposite with PPy, completely synthesized electrochemically. What we mainly wanted to test was the benefit of carboxylation in parameters such as wettability with measurement of the contact angle, specific capacitance, speed capacity, electrical resistance, and cycle stability. In practically all these parameters, a better performance was obtained compared with PPy/CNTs and PPy/GO/CNT electrodes [292]. Furthermore, the good structural stability of PPy was demonstrated when operating in the long term (95.2% retention for 5000 cycles). With similar materials, Lu et al. [297] designed electrodes composed of PPy/MWCNTs forming 3D nanostructures, in which phytic acid and CuPcTs were alternatively incorporated as dopants. Depending on the use of one or the other, respective surface areas of 39.66 and 50.17 m^2^ g^−1^ were obtained, which in turn correlate with their specific capacitances of 265 and 488 F g^−1^ (at 5 A g^−1^), respectively. However, there was a marked drop in capacitance when the current density increased from 0.67 to 5.00 A g^−1^, demonstrating a considerably lower utilization of the active sites at higher operating speeds. Compared with electrodes of only PPy/MWCNTs, the use of dopants produced better performance in general, highlighting the obtaining of lower electrical resistances [297]. 

Within this large category of electrodes based on PPy and carbonaceous materials, ternary nanocomposites of CNFs/PPy/Co_3_[Fe(CN)_2_]_2_ can be included, evaluated as supercapacitor electrodes in the work of Rawool et al. [298]. These hybrids were synthesized by in situ chemical polymerization and co-precipitation steps. Interconnected structures of CNF were obtained, serving as pathways for electronic transport, on whose surfaces nanoparticles of Co_3_[Fe(CN)_2_]_2_ and nanolayers of PPy [298] were deposited. The electrodes reached a maximum specific capacitance of 512 F g^−1^ (at 0.5 A g^−1^), being higher than that of their separate components, with cycle stability not so outstanding compared to other reports mentioned in this section (87.8% for 2000 cycles).

In another area of carbon-based structures for obtaining flexible electrodes, the use of cotton as a substrate has also proven feasible. However, it must undergo modifications to achieve good electrochemical performances in supercapacitors. Carbonization, which makes it possible to transform cotton into an electrically conductive carbonaceous material, and the incorporation of PPy as a conductive and pseudocapacitive component are approached by Sun et al. [299]. The polymer was electrodeposited on a carbonized cotton cloth substrate (CCF), forming the PPy/CCF nanocomposite with a 3D nanostructure, hierarchically meso- and micropores, of 6.5 nm and 8.1 µm, respectively. As both materials were hydrophobic, there was good adsorption of the pyrrole monomers and the corresponding polymer. When studying the performance of the material, the maximum area capacitance was 3596 F cm^−2^, with a retention of 96.5% after 4000 charge/discharge cycles. PPy/CCF was much higher relative to a CCF electrode without PPy, and its charge storage level was found to be at a better level relative to other PPy-based electrodes reported in the literature [321,322,323,324]. With the PPy/CCF nanocomposite, a flexible, solid-state symmetric supercapacitor was manufactured, with cellulose separator and gel electrolyte, exhibiting a maximum area capacitance of 500.06 mF cm^−2^, retention of 73.6% after 2000 cycles and densities maximum energy and power of 1.18 mWh cm^−3^ and 84.4 mW cm^−3^, respectively. Furthermore, when subjected to 200 bending cycles of 0–150°, the area capacitance was retained at 75.6%. Although it showed high performance in charge storage compared to other supercapacitors reported with PPy, with good flexibility, cycle stability was possibly impaired by side reactions between the gel electrolyte and the electrode [299].

In the area of PPy nanocomposites and metal oxides, in order to optimize the performance that the latter can offer [300,301,302], an investigation by Xu et al. [300], where they reported the hydrothermal synthesis in a single container of nanocoral-type structures composed of Fe_2_O_3_ with PPy coatings. At reaction temperatures of 120 and 180 °C, surface areas of 150 and 51 m^2^ g^−1^, respectively, were obtained, but in the second case, more PPy was deposited. Something fundamental emerged from this, and that is that the nanocomposite with the highest surface area was not the one with the highest capacitance, but rather the one synthesized at 180 °C, with the highest PPy content—represented by the ratio between C+N/Fe atoms at the superficial level—and wider pores. Therefore, it is a demonstration that the optimization of the surface area does not always imply a higher electrochemical performance if it is not accompanied by more availability of active sites, a balance that would have been achieved in the case of 180 °C. Regarding the charge storage mechanism, both PPy and Fe_2_O_3_ showed pseudocapacitive behavior, but the polymer also allowed better electrical and ionic conductivities [300]. When evaluating electrodes with the nanocomposite obtained at 180 °C, a maximum specific capacitance of 560 F g^−1^ was reached, with retentions of 62.5% at 40 A g^−1^ and 97.3% after 20,000 operating cycles. In the context of PPy composite electrodes, they are outstanding performances, especially in terms of long-term structural stability, showing a low degradation of PPy and Fe_2_O_3_, as well as a good synergy between these materials.

With the same class of materials, although using MnO_2_ as metal oxide, a different treatment of PPy can be found, as reported by Zhu et al. [301]. From PPy nanobars obtained by chemical means and their subsequent carbonization at 900 °C, N-doped CNTs (N-CNTs) were obtained. In the final stage, hydrothermally, MnO_2_ nanoparticles were synthesized and anchored on the surfaces of the N-CNTs. The nanocomposite with the highest specific capacitance at 0.5 A g^−1^ (392.8 F g^−1^) corresponded to 59.5% by mass of MnO_2_. Notwithstanding this, among the nanocomposites prepared, it was the one that showed the lowest surface area, pore volume, electrical conductivity, speed capacity, and cycle stability [75]. Finally, to build an asymmetric supercapacitor (N-CNTs/MnO_2_//N-CNTs), an intermediate-performance nanocomposite was chosen (366.5 F g^−1^ at 0.5 A g^−1^), achieving a device with good long-term stability (91.6% retention, at 5000 cycles), verifying the good anchorage of the MnO_2_ nanoparticles on N-CNTs [301]. 

Along the same lines of using PPy as a precursor to obtaining a carbonaceous material doped with N, Sun et al. [302] reported the synthesis of relatively ordered nw of V_2_O_5_, on nanosheets of a 3D airgel of G doped with N (N-GA), from the carbonization of PPy. In this way, 3D and 1D nanostructures were combined, exhibiting abundant micrometric-scale porosity for the airgel and nanometric channels between the nw, reaching a surface area of 416 m^2^ g^−1^. When evaluating the electrochemical performances, a maximum specific capacitance of 710.5 F g^−1^ (at 0.5 A g^−1^) was reached, with retentions of 50% at 10 A g^−1^ and a remarkable 95% after 20,000 cycles [302].

In order to improve the yields that can be achieved using ternary sulfides, which are by themselves good conductors and possess higher redox activity than their binary counterparts, in a study by Liang et al. [303], the synthesis of NiCo_2_S_4_@Ni(OH)_2_@PPy nanotubes, core-shell type, was reported. The incorporation of Ni(OH)_2_ and PPy nanolayers would have been in order to increase the surface area, offer greater active sites with pseudocapacitance, and improve the stability of NiCo_2_S_4_. Of course, PPy would also have enhanced the electrical conductivity of the electrodes, something that could benefit the speed of charge/discharge processes, especially at high current densities, and the maximization of the use of redox-active sites. The NiCo_2_S_4_@Ni(OH)_2_@PPy electrodes showed behaviors similar to those found in battery electrodes, possibly due to the intercalation pseudocapacitance of NiCo_2_S_4_ [303]. Related to NiCo_2_S_4_@Ni(OH)_2_ and NiCo_2_S_4_@PPy samples, the best performances were obtained for the combination of the three materials, reaching a remarkable maximum specific capacitance of 2838.8 F g^−1^ (9112.5 mF cm^−2^), with 96.2% retention after 3000 cycles, for one electrode [303]. 

Also, with a core-shell structure, there is a report by Wang et al. [304], in which they used CoSe_2_ selenides in the form of nanoflowers surrounded by uniform electrodeposited PPy nanolayers. Both materials contributed synergistically to charge storage, with PPy being fundamental in the electrical conductivity and stability of CoSe_2_. The manufactured electrodes were used in flexible asymmetric supercapacitors with activated carbon anodes, developing an area capacitance of 226 mF cm^−2^ (at 18 mA cm^−2^) and a maximum energy density of 2.63 mWh cm^−3^, being at a level higher than other similar asymmetric devices, with some inorganic component [325,326,327,328].

In the field of PPy, in combination with metal oxides and some carbon-based material, there is a report that stands out for obtaining a nanocomposite with a high level of specific capacitance. Wang et al. [305] manufactured electrodes composed of a graphite foam current collector modified with—OH groups, where they hydrothermally deposited nanoribbons of the bimetallic oxide MnCo_2_O_4_, on whose surfaces they synthesized vertical arrays of PPy nanofilms by oxidative chemical polymerization. Mention is made of a probable formation of coordination bonds between the PPy chains and the Mn^3+^ and Co^3+^ cations, which would have been beneficial for the fixation of the nanofilms [305]. In addition to the pseudocapacitance provided by these, they served as support for the electronic trajectories to and from MnCo_2_O_4_. For the graphite/MnCo_2_O_4_@PPy electrodes, a maximum capacitance of 2364 F g^−1^ was reached, with a speed capacity of 55.2% retention when increasing the current density 50 times. This is explained by the optimized morphology and the abundant redox activity, mainly of nanostructured PPy, and supported by MnCo_2_O_4_ [305]. Another case in which the combination of a metal oxide, PPy, and a carbonaceous material was used is a study by Zhu et al. [306], in which the synthesis of ternary nanosheets of rGO@PPy/Fe_3_O_4_ was reported, with the assistance of PS-b-PEO as a removable template, for the formation of mesopores (14–23 nm). The nanocomposite exhibited a 2D sandwich-type structure, with rGO wrapped in nanolayers of PPy, where nanoparticles of Fe_3_O_4_ with ~3 nm of average diameter were dispersed. When evaluating its electrochemical performance, a maximum capacitance of 1006 F g^−1^ (at 1 A g^−1^) was reached, with a retention of 74.5% when reaching 20 A g^−1^ and of 85% after 5000 cycles. For the optimization of the specific capacitance, the formation of the mesopores was fundamental, in particular, when these were 14 nm on average, and also the incorporation of the Fe_3_O_4_ nanoparticles, which would have contributed to pseudocapacitance [306]. 

As an alternative to the use of metal oxides, metal hydroxides have been used in ternary nanocomposites, as in the case reported by Li et al. [303], where they synthesized a 3D GO-sulfonated/Ni(OH)_2_/PPy nanostructure, by means of a hydrothermal stage and another of oxidative chemical polymerization. GO sulfonation was carried out to increase its hydrophilicity and to facilitate the insertion of ions. On the GO-sulfonated sheets, Ni(OH)_2_ nanosheets and PPy coatings were incorporated to provide pseudocapacitance to the nanocomposite [329]. The 3D nanostructures presented irregular surfaces on micrometric scale particles, with abundant concentrated mesopores in a size range of 2–10 nm. With the optimization of the number of active sites, the best charge storage was obtained for a molar ratio of 2:1 between PPy and Ni(OH)_2_/GO-sulfonated, with a maximum specific capacitance of 1632 F g^−1^, although with a not-so-outstanding cycle stability. The synthesized electrodes were used to manufacture asymmetric solid-state supercapacitors, with AC anodes, with a maximum capacitance of 224 F g^−1^ and an energy density in the range of 79.6–48.7 Wh kg^−1^, being superior to other previous reports of electrodes with metal oxides or hydroxides [186,329,330,331,332].

The incorporation of noble metals in ternary nanocomposites containing PPy and some carbonaceous material corresponds to another approach that has been shown to be valid for achieving good yields [308,309,310]. Chen et al. [308] reported the manufacture of flexible and self-healing or repairable electrodes composed of hydrogels of PAM/CNTs fibers interconnected with Au nanoparticles and PPy nanoparticle coatings. Furthermore, the same hydrogel, but free of CNTs, was used as the medium for the electrolyte and Ag nw films as current collectors. In addition to the CNTs, the PPy chains would have been the main contributors to the capacitance of the devices. The deformable supercapacitor reached an area capacitance of 885 mF cm^−2^, maximum area energy density of 123 µWh cm^−2^, long-term stability with retention of 93% after 10,000 cycles, being operational up to 800% deformation [308]. Within the field of deformable supercapacitors, this stands out from several reports. Regarding self-repair, with the assistance of a NIR laser, the capacitance was retained at 80% after 10 cut-repair cycles, reflected in CV and GCD curves. In these processes, the reconstruction of the bonds between Au (or Ag) and the S atoms of the PAM chains was fundamental [308].

Another case in this area but using nanoporous Au on PET as a substrate/flexible current collector through an electrochemical method, is the work of Purkait et al. [309], who synthesized microsupercapacitors with PPy-rGO 3D hybrids as electroactive components. Solid-state microsupercapacitors with PVA/HClO_4_ gel electrolyte developed a volumetric capacitance of up to 245.34 F cm^−3^ (at 0.6 mA cm^−3^) and a maximum energy density of 98.48 mWh cm^−3^, being higher than others reports of microsupercapacitors based on composites of CP and carbonaceous materials [333,334,335,336,337]. Likewise, good capacitance retention was achieved after 10,000 cycles (85.9%), demonstrating the structural stability of the manufactured electrodes. 

The combination of PPy with biomass-derived materials can be found in some recent reports of electrode designs [311,312,313,314]. Regarding the use of plant materials, Yang et al. [311] electrodeposited conformal layers of PPy at the nanoscale on the inner and outer surfaces. That is, the hollow fibers acted as molds and supports for the PPy chains. Using DC fibers as a substrate, they manufactured flexible solid-state supercapacitors with specific capacitances of up to 341.64 ± 2.61 F g^−1^ (785.78 ± 6.01 mF cm^−2^), being superior to electrodes without vegetable fibers, without suffering a drastic drop at higher current densities. These were fundamental because they allowed a better homogeneity in the PPy layers, compared to direct electrodeposition on the carbon fibers, and, in addition, they optimized the spaces for the rapid ionic diffusion through their channels towards the active sites of the CP [311]. The stable operation of these devices was tested for 100 bending cycles, with 90% retention in capacitance, and up to three devices were connected in series to power an LED light. 

The case of electrodes made of cellulose, where the interactions by bridges of H between these and PPy are important, highlights the work of Mo et al. [312]. To obtain spiral-shaped supercapacitors, they synthesized composites with cellulose nanofibers as reinforcements inside microfibers of rGO nanosheets covered by ~1.7 nm layers of PPy, using a wet-spinning method. The cellulose nanofibers (CNFs) would have optimized the spaces between rGO@PPy nanofibers, facilitating the access of ions to the active sites and providing better breaking stress (364.3 MPa). The good control in the amount of PPy was decisive in reaching a good balance between the availability of active sites and the resistance to ionic diffusion, obtaining the best yields for a mass ratio of 1:1 of GO:pyrrole during the synthesis [312]. This electrode made it possible to achieve a good speed capacity, with a maximum capacitance of 334 mF cm^−2^ and retention of 98.8% when increasing the current density 10 times when evaluating flexible supercapacitors with H_2_SO_4_ aqueous electrolyte. Notable was its long-term behavior, retaining ~100% capacitance after 2000 cycles [312]. Another strategy in the treatment of cellulose was applied by Zhang et al. [313], which underwent lyophilization and pyrolysis steps to form carbon aerogels (CAs). Then, through oxidative chemical polymerization in situ, they deposited PPy nanolayers on the surfaces of the CA nanofibers, forming core-shell-like structures. This electrode shows 88% retention of capacitance after 10,000 charge/discharge cycles. Symmetric supercapacitors delivered maximum capacitance and energy density of 268.5 F g^−1^ and 23.8 Wh kg^−1^, with superior performances than their separate components [313].

Kulandaivalu and Sulaiman [312] designed and evaluated structurally more complex nanocomposites with bilayer morphology as supercapacitor electrodes. For the nanocomposites denoted as r-PGMGN, an outermost layer was composed of MWCNTs/rGO and cellulose nanocrystals (NCC), while the innermost was based on rGO/PPy nanofilms. This shows greater surface area, total pore volume, and electrochemical response in CV with respect to the separate layers. With respect to previous reports, the highest performances were obtained in terms of maximum energy density (44.6 Wh kg^−1^) and long-term stability, with retention in capacitance of 90% after 10,000 cycles and notable retention of 92.5% at an increased current density of 12 times. It should be noted that cellulose nanocrystals had a greater impact in terms of mechanical reinforcement and spacing in the structures of rGO/MWCNTs, but not directly on PPy, which was supported by the rGO nanosheets [312].

As with PANI, the strategy of manufacturing electrodes with PPy hydrogels has been tested without the need to incorporate any carbonaceous or inorganic component, as illustrated by the studies described above. For example, Bo et al. [315] synthesized PANI hydrogels using oxidative chemical polymerization with the assistance of SDS as a surfactant and dopant without using crosslinking agents. Additionally, it was found that the type of oxidant used had an impact on the morphology of the hydrogel. For example, using APS nanoparticles of ~45 nm were obtained, while with FeCl_3_ and Fe(NO_3_)_3_, they were of ~75 and 100 nm, respectively [[105]]. In the dry state, the PPy hydrogel showed an electrical conductivity of up to 5.7 S cm^−1^. When evaluating an electrode with the optimized hydrogel synthesized with APS, a maximum specific capacitance of 328 F g^−1^ (at 1 A g^−1^) was obtained, retaining 90% after 3000 cycles. The good speed capability demonstrated the rapid ion access to active sites, bringing the device to a current density of up to 8 A g^−1^. On the other hand, a solid-state flexible symmetric supercapacitor gave specific capacitance and energy density values of up to 170 F g^−1^ and 4.13 Wh kg^−1^, respectively [315]. Interlinking species were a concern in the work of Guo et al. [316]. In this case, they used CuPcTs with this function to interconnect PPy chains in the nanofibers (~50 nm thick), but also, the coordination complex with its sulfonate groups acted as dopants. The nanostructure of the hydrogel, with abundant and fast pathways for electron and ionic transport, made it possible to obtain electrodes with area capacitances of 486 and 260 mF cm^−2^, for 1.25 and 12.5 mA cm^−2^, respectively [316]. Long-term stability, although tested for 100,000 cycles, suffered a remarkable 50% loss in capacitance, demonstrating degradation of polymer chains, under numerous cycles of volumetric changes with insertion/disinsertion of ions. Symmetrical supercapacitors based on these electrodes demonstrated stable performance under bending and torsion and, furthermore, for 2000 bending cycles they lost only 3% of capacitance.

Electrodes composed of PPy hydrogels with higher levels of capacitance were designed by Yang et al. [317]. In PPy chains, in 1D ordered arrangements along nanofibers, by a self-ordering mechanism in TB molecules, sulfonic acid groups are arranged in a way that favors the aligned ordering of PPy chains by electrostatic interactions and H bridges [317]. The best electrochemical performances, with the highest response in CV curves, were achieved for hydrogels symbolized as PPy-TB_4_, with the highest electrical conductivity, the smallest pore size and nanofiber thickness and not necessarily with the largest surface area (26,312 m^2^ g^−1^), within the variety of prepared hydrogels (up to 64,575 m^2^ g^−1^). The electrodes delivered a maximum capacitance of 707 f g^−1^ at 0.5 A g^−1^, with retention of ~76.4% when increasing the current density 10 times, demonstrating the good accessibility of the ions to active sites and the fast electronic conduction by increasing the speed of the processes. What was lacking in this study was to test the long-term stability of these hydrogels since it is usual to enter some degree of degradation of the materials and an increase in electrical resistance when operating during numerous charge/discharge cycles.

A report by Xin et al. [318] is part of the PPy pyrolysis strategy to obtain N-doped carbonaceous structures. Using PPy, APS, and a pore-generating species called F127, they synthesized hierarchically porous carbonaceous nanospheres (with meso- and micropores) and doped with N and S atoms (N, S-HPCNs). These nanospheres exhibited a capacitance of up to 416 F g^−1^ higher, compared to similarly doped nanostructures, but only microporous (N, S-CNs) and with a very good retention of 95.8% after 5000 cycles [318]. This demonstrated the importance of incorporating mesopores above 2 nm, facilitating a quick and less tortuous ionic diffusion, while microporosity was more relevant in terms of the availability of active sites. 

Atomically thick two-dimensional (2D) materials have been presented as one of the latest improved designs for supercapacitors due to their fascinating chemical and physical characteristics and their simplicity of property tuning, attracting the attention of several research groups in the area. For example, Lv. et al. [338] developed a strategy for a multifunctional e-textile based on PPy and cotton/spandex knitted fabric (KCSF) decorated with rose-like silver flowers. Furthermore, the solid-state supercapacitor is fabricated using PPy-coated cloth electrodes, demonstrating a high areal capacitance of 978.9 mF cm^−2^ at 1 mA cm^−2^, an energy density of 80.3 μWh cm^−2^ and a power density of 0.38 mW cm^−2^. Another report by Vandana et al. [339] shows the production of a ternary quantum dot composite electrode of tin oxide/graphene oxide/polypyrrole (SGP). The fabrication of the supercapacitor device that uses this electrode with PVA/KOH gel electrolyte as a separator reports that the maximum specific capacitance reached was 928.56 F g^−1^ at 40 mV s^−1^ and the energy density of 25, 6 Wh kg^−1^ with a high power density of 4098 W kg^−1^ after 11,000 successive cycles at a current density of 1 A g^−1^.

### 7.3. PEDOT

The nanostructures of PEDOT and other derivatives of PTh have been classified in the following categories: combination with carbonaceous materials (Table 4, entries 01–08); combination with inorganic materials (Table 4, entries 09–20); in hybrids with carbonaceous and inorganic materials (Table 4, entries 21–24); and others (Table 4, entries 25–27). The selected cases will then be reviewed in detail.

Among the publications found in this area, particularly in relation to the combination with rGO, Kumar et al. [340] showed the manufacture of flexible electrodes based on PEDOT: PSS/rGO porous nanocomposites on DC current collectors using a spray deposition technique. Unlike electrodes with only PEDOT:PSS, the incorporation of rGO produced an important microporosity, where the nanofilms would have been surrounded by PEDOT:PSS in a homogeneously dispersed form [340]. Along with this, the conductivity of the electrodes was increased, by optimizing the amount of rGO and doping with DMSO and H_3_PO_4_, reaching up to 1050 S cm^−1^. This had a positive impact on specific capacitance levels and speed capability by increasing CV sweep speeds. It is worth mentioning the cycle stability of the manufactured supercapacitors, retaining ~100% capacitance after 2000 charge/discharge cycles. Furthermore, under high deformation, with a bending radius of 0.5 mm, the capacitance was retained at ~94%, demonstrating the stability of the devices, with a good fixation of PEDOT:PSS/rGO on the DC substrates [340]. 

With less difficulty, it is possible to find recent publications in which CNTs have been used to form nanocomposites with PTh derivatives, mainly with PEDOT. Due to the possibility of manufacturing flexible and independent electrodes, what is reported by He et al. [341], demonstrated the synthesis of a sponge-like, porous 3D nanostructure based on MWCNTs and homogeneous electrodeposited PEDOT layers. They achieved a highly interconnected fiber nanostructure, acting as pathways for electronic transport and abundant porosity for ionic diffusion towards redox active sites. The incorporation of PEDOT layers adjusted to the surface of MWCNTs was significant in terms of the increase in specific capacitance, since for electrodes with only MWCNTs, up to 20 F g^−1^ was obtained, on the other hand, for the nanocomposite with an optimized amount of PEDOT, 147 F g^−1^ was reached (at 0.5 A g^−1^). Regarding cycle stability, a good performance was obtained, with a retention of 94.7% in capacitance, after 3000 operating cycles [341]. The benefits of the adequate porosity of the nanocomposites, which facilitated a rapid ionic diffusion, were reflected in the retention of the capacitance of 70% by increasing the current density 20 times. An outstanding structural characteristic was its ability to withstand mechanical compression, as a sponge does, keeping its operability intact after 1000 cycles of compression at 50% [341]. This area of materials can also include a type of electrodes reported in a publication by Li et al. [342], composed of SWCNTs, PEDOT:PSS, and copper hexacyanoferrate nanoparticles (CuHCF). The PEDOT:PSS chains served as nanolayers in both SWCNTs and nanoparticles. Additionally, the highly interconnected networks of SWCNTs/PEDOT:PSS allowed abundant pathways for electron transport and nano-scale porosity for ion diffusion. The ternary nanocomposite exhibited an electrical conductivity of up to ~1470 S cm^−1^, higher than SWCNTs and PEDOT:PSS separately (1.12 and 1450 S cm^−1^, respectively). The pseudocapacitive activity for charge storage was mainly provided by the CuHCF nanoparticles and, secondly, by the PEDOT chains. Additionally, as stated in the study, the wettability of the material would have improved with the inclusion of PEDOT:PSS, with an increase in the contact angle from 118° to 89°, which would have benefited ionic permeability, improving insertion towards the site’s assets [342]. This type of study shows that the inclusion of PEDOT:PSS is not necessarily to provide abundant active pseudocapacitance sites, even when it partially contributes. It is as a consequence of the better electrical conductivity provided by PEDOT:PSS that optimization can be achieved in the levels of capacitance and speed capacity. Notably, one of the best performance parameters was cycle stability, with a retention of 95% after 15,000 cycles [342]. To test the application of SWCNTs/PEDOT:PSS/CuHCF, asymmetric supercapacitors were manufactured, where such nanocomposites were used as cathodes and WO_3_/SWCNTs doped with Mo as anodes. These devices achieved a volumetric energy density of up to 30.08 Wh L^−1^, being tested in series circuits to power LED lights. 

The combination of carbonaceous and CP materials, such as PTh and derivatives, under the appropriate morphologies, allows for obtaining flexible energy storage devices. Zhou et al. [343] prepared a nanocomposite based on horizontally aligned CNTs (HACNTs) and a poly(3-methylthiophene) (P3MT) coating, testing it as an electrode of a flexible asymmetric supercapacitor. For the synthesis of HACNTs/P3MT, a two-stage method was performed: first, a mechanical rolling/flattening treatment of CNTs was applied, and second, a oxidative chemical vapor deposition (COVD) of P3MT was performed on HACNTs. The arrangement of HACNTs increased the density of the material, which impacted the volumetric electrochemical performance and enabled the flexibility of the electrode [343]. While the P3MT coverage (~5 nm thick) added the pseudocapacitance mechanism for charge storage, something that the single structure of HACNTs lacks. In addition, the conformal coverage of P3MT could easily adapt to the deformation of HACNTs, which was favorable in terms of mechanical stability during the deformation and charge/discharge processes, avoiding the rapid collapse of the electrode. Something also beneficial for the electrochemical performance was the distance between HACNTs/P3MT nanofibers and their orderly alignment, allowing easy ionic diffusion to redox-active sites and better electrical conductivity. In the latter, the methyl substituent in the third position of the thiophene ring was key since it increased the probability of obtaining aligned chains, favoring electrical conductivity [343]. In relation to HACNTs without P3MT and to a bucky-paper electrode based on a disordered arrangement of CNTs, the nanocomposite showed better electrochemical responses, higher levels of capacitance, and cycle stability. It reached up to 3.11 and 1.81 F cm^−2^ for current densities of 5 and 200 mA cm^−2^, respectively. In order to increase the potential operating window, a flexible solid-state asymmetric supercapacitor was manufactured, with HACNTs/P3MT and HACNTs, as positive and negative electrodes, respectively, using flexible substrates based on PDMS [343]. An area capacitance of up to 0.64 F cm^−2^ and a maximum energy density of 1.08 mWh cm^−2^ was achieved, being superior to some flexible electrodes of state of the art, based on carbonaceous composites with some inorganic component [356,357], CP [358] or MOF [359]. To test performance under strain, charge/discharge and bending cycles were applied simultaneously, where after 5000 cycles, the area capacitance only decreased by 8% [343]. 

As usual, metal oxides can be found in combination with CP in nanocomposites for supercapacitor electrodes [344,345,346,347,348]. A recent case corresponding to a study by Chetana et al. [360] shows the synthesis of CoS/MXene/PANI and CoS/MXene/PEDOT ternary compounds using supercritical fluid (SCF). These coin cells showed an improved specific capacitance in CoS/MXene/PEDOT (331.1 F g^−1^) over CoS/MXene/PANI (246 F g^−1^) at 2 A g^−1^. It was discussed that the inclusion of PEDOT in this type of cells improved the capacitance performance, with respect to another widely used polymer, such as PANI, due to its lower resistance and higher specific capacitance. Another research group led by Bi et al. [344] reported the preparation of V_2_O_5_ nanofibers covered by 5 nm thick PEDOT conformal nanolayers, synthesized by a vapor phase polymerization method, forming structures in the form of core-shell type coaxial nw (G-V_2_O_5_/PEDOT). In addition to nanostructuring the oxide, oxygen vacancy gradients were formed in the nanofibers to increase electrical conductivity. On the other hand, when depositing the PEDOT nanolayers, it was also sought to improve the conductivity and, even more, to increase the pseudocapacitance and improve the structural stability of the nanocomposites [344]. By Nyquist diagrams, it was determined that the resistance to charge transfer was 2.1 Ω, being lower in relation to nanofibers without PEDOT and to V_2_O_5_ without nanostructuring. Regarding the specific capacitance of the electrodes made up of the nanocomposites, a maximum of 614 F g^−1^ was reached, and an energy density of up to 85 Wh kg^−1^ was achieved in a symmetric supercapacitor. Additionally, excellent long-term stability was achieved, with 122% with respect to the initial capacitance, after 50,000 cycles and better levels of capacitance, with respect to its components separately [349]. This last parameter is within the highlights in the context of this review. The remarkable long-term operability demonstrated the appropriateness of the proposed design, with good protection of V_2_O_5_, as well as a stable fixation of the PEDOT chains, avoiding the pulverization of the materials during the charge/discharge cycles.

In another case, using V_2_O_5_ as a precursor material combined with PEDOT, Lee et al. [345] reported the synthesis of ammonium vanadate nanofibers ((NH_4_)_2_V_6_O_16_), interspersed with PEDOT to improve their electrical conductivity, using a sonochemical method. At the time of EDOT polymerization, with V_2_O_5_ as an oxidizing agent, (NH_4_)_2_V_6_O_16_ were simultaneously synthesized, and the PEDOT chains formed intercalated layers between the sheets of inorganic material [345]. The maximum specific capacitance achieved for a nanocomposite electrode was 202 F g^−1^, being higher than that obtained for a similar nanocomposite synthesized by a reflux method (120 F g^−1^) and nanofibers without intercalated PEDOT (106 to 97 F g^−1^). For the (NH_4_)_2_V_6_O_16_/PEDOT nanocomposites obtained by sonochemistry, an electrical conductivity of 4.1·10^−2^ S cm^−1^ (2.2·10^−3^ S cm^−1^ without PEDOT) was obtained, mesopores of average size 4.68 nm, but it did not show the highest surface area compared to nanofibers without PEDOT [345]. The loss in surface area was compensated with the active redox sites offered by PEDOT.

From dispersions of PEDOT:commercial PSS combined with a hydrated metal oxide of RuO_2_ × 1.18H_2_O, Chang et al. [346] reported flexible electrodes with a non-negligible maximum specific capacitance of 630 F g^−1^ at a high current density of 15 A g^−1^. Controlling the amounts of both materials, they obtained films composed of PEDOT:PSS nanosheets interspersed with well-dispersed nanoparticles of RuO_2_ × 1.18H_2_O (~16 nm in diameter). Such morphological characteristics of the nanocomposite, together with the PEDOT:PSS treatment with DMSO to improve electrical conductivity (306 S cm^−1^), would explain the remarkable capacitance at an unusually high current density, with abundant pseudocapacitance redox active sites. On the other hand, both PEDOT:PSS and the hydrated layers of RuO_2_ × 1.18H_2_O would have facilitated the diffusion of ions within the material due to their hydrophilic characteristics [346]. Additionally, the performances achieved in symmetric supercapacitors were remarkable, retaining 94% of the capacitance when increasing the current density 10 times, a long-term stability of 91.5% retention after 5000 cycles, and an energy density of up to 19 Wh kg^−1^.

In the area of manufacturing electrodes occupying the promising MXenos, there are also recent reports where it has been combined with PEDOT [349,350]. In order to achieve flexible fiber-shaped supercapacitors, Zhang et al. [349] designed using wet-spinning technique fibers composed of MXeno Ti_3_C_2_T_x_ nanosheets, interspersed with PEDOT:PSS chains, MXPY. Both materials offered redox active sites to store charge through pseudocapacitance, but in addition, PEDOT acted as a conductive binder to keep the Ti_3_C_2_T_x_ nanosheets stable. By optimizing the amounts of these components, a remarkable electrical conductivity of 1489 S cm^−1^ and electrodes with a volumetric capacitance of up to 614.5 F cm^−3^ (361.4 F cm^−3^ for a symmetric supercapacitor) were achieved [340]. Some key factors were the treatment of PEDOT:PSS with H_2_SO_4_ to increase the electrical conductivity (with the removal of PSS), the abundance of channels between the nanosheets for ionic diffusion, and the high order of alignment between PEDOT and Ti_3_C_2_T_x_ allowing low tortuosity trajectories for ions [349]. Manufactured, flexible solid-state supercapacitors exhibited remarkable long-term stability (~96% retention for 10,000 cycles) and low stretch cycles (96% to 98%, 200 cycles).

From an electropolymerization method to deposit PEDOT nanolayers on ternary nanofibers composed of PVA, graphene quantum dots (GQDs), and Co_3_O_4_, Syed Zainol Abidin et al. [351] presented the manufacture of electrodes with good stability (96% retention, 1000 cycles). It is one of the few reports found where GQDs have been used in supercapacitor electrodes, being significant for better electrical conductivity. The incorporated layers of PEDOT were adopted according to the surfaces of the nanofibers (44 ± 13 in diameter), with a uniform distribution. Using curves obtained by CV and GCD, it was found that the main energy storage mechanism was by means of an electrical double layer [351]. Furthermore, long-term stability, with little degradation of PEDOT, would have occurred due to the good synergistic combination with Co_3_O_4_, which would form stable and well-anchored structures. What could be missing in this study was a comparison between nanofibers with and without PEDOT, studying the differences in conductivity, capacitance, and long-term stability and thus making explicit the benefits of their incorporation. On the other hand, the study focused on the best performance obtained by the incorporation of Co_3_O_4_ in terms of the different parameters mentioned. In order to point out a couple of examples, without Co_3_O_4_, the nanofibers had greater internal electrical resistance, and their stability was only 86% after 1000 cycles of operation [351].

A strategy such as the previous report can be found in Mohd Abdah et al. [352]. They also electrodeposited uniform PEDOT nanolayers, although in this case, on PVA-GO-MnO_2_ microfibers. When manufacturing symmetric supercapacitors with PVA-GO-MnO_2_/PEDOT electrodes, maximum values of specific capacitance and energy density of 144.66 F g^−1^ and 9.60 Wh kg^−1^ were obtained, not being at such prominent levels in the context of the present revision. On the other hand, for electrodeposited PEDOT films, PVA-GO/PEDOT and PVA-MnO_2_/PEDOT microfibers, capacitances of 62.86, 94.73 and 107.22 F g^−1^ were obtained, respectively, demonstrating the best charge storage in the microfibers of PVA-GO-MnO_2_/PEDOT, reflected in the GCD curves [352]. In addition to not having empirically contrasted between materials with and without PEDOT, which would have given more fundamental clues about the optimization that PEDOT could offer, the PVA-GO-MnO_2_/PEDOT microfibers exhibited structures with more tortuous spaces, making ionic trajectories more difficult, compared to PVA-MnO_2_/PEDOT [352]. Therefore, the PVA-GO-MnO_2_/PEDOT microfibers would have required further optimization in terms of adequate spaces for ionic diffusion. 

Regarding the strategy of carbonizing CP, Yao et al. synthesized independent electrodes composed of a ternary nanocomposite of Co_9_S_8_/PEDOT: multilayer intercalated PSS-carbonized/rGO. For electrodes designed with an optimal amount of PEDOT:PSS, a specific capacitance of up to 788.9 F g^−1^ was reached, with a retention of over 100% after 10,000 cycles, demonstrating the good level of energy storage and the excellent stability of the electrodes materials before charge/discharge cycles [354]. Regarding the level of capacitance, it is below some state-of-the-art reports based on cobalt sulfides, but in relation to cycle stability, it has been shown to be superior [361,362,363,364,365]. For an asymmetric supercapacitor with Co_9_S_8_/PEDOT cathode: PSS-carbonized/rGO and AC anode, a remarkable retention of 94.2% was obtained after 10,000 cycles, delivering a maximum energy density of 19.6 Wh kg^−1^.

Due to their high porosity and redox activity, covalent-organic frameworks (COFs) have been considered candidates in energy storage applications. However, they are intrinsically electrical insulators. A strategy that enables its use is based on the incorporation of CP chains, providing electrical conductivity and pseudocapacitance. In this way, the work of Wu et al. [354] has reported the preparation of a COF based on anthraquinone units (AQ-COF), in whose nanochannels PEDOT chains were deposited, from a solid-state polymerization in situ, of 2,5-dibromo-3,4-ethylenedioxythiophene. The PEDOT@AQ-COF nanocomposite showed a conductivity of ~1.1 S cm^−1^, lower than PEDOT (1.9 S cm^−1^) but much higher than AQ-COF (~10^−10^ S cm^−1^), as did a larger surface area [354]. Within its electrochemical performance, we can highlight the maximum specific capacitance of 1663 F g-1 (at 1 A g^−1^), its speed capacity, and its cycle stability, with an increase in capacitance after 10 000 cycles, which is not usual. The way of storing charges would have been produced by double electrical layer and pseudocapacitance, through the participation of PEDOT and the redox groups of AQ-COF. In this, the transfer of electrons from the PEDOT chains to said AQ-COF redox active groups could be fundamental. In addition to the above, the morphology of nanochannels facilitated the diffusion of ions, impacting the kinetics, speed capacity, and specific capacitance of the electrode [354]. The increase in this last parameter, as the charge/discharge cycles advance, could be due to the rearrangement of the PEDOT chains within the nanochannels, increasing the number of active sites available for redox reactions that allow charge to be stored. Thus, the introduction of PEDOT in the AQ-COF nanochannels obtained a material with superior performance, both when compared with a PEDOT/AQ-COF nanocomposite (with PEDOT outside the nanochannels) and with respect to other reports of supercapacitors based on COFs [362,363,364,365,366].

In a different approach to obtaining flexible electrodes in supercapacitors, without the incorporation of carbonaceous materials or metal oxides, independent nanostructured structures are adopted, which do not require the presence of additional substrates or current collectors. In these cases, there is a risk of obtaining structures with low cycle stabilities and electrical conductivity, which is why rational designs are used and the incorporation of substances with redox activity improve the electrochemical performance. One of the first cases of PEDOT-based electrodes reported in this way corresponds to the work carried out by Ni et al. [355]. A PEDOT nw film was prepared by a micellar self-assembling chemical pathway, assisted by soft-tempering and vacuum filtration. An independent nanostructure was obtained to be used as an electrode without the need for conductive substrates. Something very relevant in this study was the incorporation of a PDA additive in the electrolyte in order to increase the specific capacitance of the device due to its active redox sites [355]. The nanostructure demonstrated an entangled nw morphology, 10.67 nm in diameter, and reached an average electrical conductivity of 1340 S cm^−1^. The achieved capacitance was quite dependent on the amount of PEDOT up to a certain point; with more of the electrochemically active material, the pathways for electron transport increase, and there are more active sites available for the electrolyte. Although the incorporation of PDA significantly increased the areal capacitance of the electrode (477.9 F cm^−2^, 5 mA cm^−2^), with respect to an electrolyte of only H_2_SO_4_ (212.6 F cm^−2^, 5 mA cm^−2^), it exhibited a lower capacity for speed and cycle stability, due to lower ionization and mobility of PDA, as well as loss of capacitance via self-discharge [355]. On the other hand, good stability was demonstrated in bending tests of 0–180°, with almost no change in capacitance after 5000 cycles. With these PEDOT nw electrodes, flexible symmetric solid-state supercapacitors were built, reaching a maximum areal capacitance of 413.5 mF cm^−2^ and energy and power densities of up to 48.3 µWh cm^−2^ and 16.8 µW cm^−2^, respectively, being at higher levels than some PEDOT-based reports included in composites [367,368,369]. With a series connection of three supercapacitors, an LED light with an operating voltage of 2.5 V [355] was powered. Therefore, not only the nanostructuring of PEDOT was decisive, but also the modification with PDA, demonstrating the importance of different modifications in the materials of a supercapacitor, beyond the mere electrode.

### 7.4. Copolymers and Derivatives of CP

In addition to the nanostructures with the CP considered so far, there is work on other types of less conventional structures to manufacture supercapacitor electrodes. In this category, there are some derivatives of PANI, copolymers, a combination of polymers, CMPs, CAPs, structures based on quinones, or on indole, among others, integrated (or not), in hybrids with another class of materials (see Table 5, for yields).

In the case of CP derived from PANI, there are recent reports of its nanostructuring combined with carbonaceous materials to improve electrical conductivity and structural strength. For example, Jena et al. [370] chemically deposited PMTA nanolayers on CNTs, obtaining nanotubes with diameters of ~25 nm, which in turn were anchored in rGO sheets for better stability. The pseudocapacitance of PMTA and the double electrical layer in rGO/CNTs made it possible to reach a specific capacitance of up to 658 F g^−1^ (at 0.5 A g^−1^), being superior to similar nanocomposites of rGO/CNTs@PANI studied. An important factor in the best yields corresponds to the influence of the electro-donor group—SCH_3_ in the *ortho* position, where the free pair of electrons in S would contribute with electronic density to the benzene rings, increasing the degree of conjugation [384]. This would improve electrical conductivity and cause stronger p-p interactions, accelerating charge transfer between PMTA and CNT [380]. Another notable aspect was the better speed capacity of CNTs@PMTA, which would have been related to easier access of ions in their mesopores, exhibiting a greater surface area (29 m^2^ g^−1^) than CNTs@PANI (22 m^2^ g^−1^). Additionally, the better p-p type interaction between PMTA and CNTs, with respect to PANI and CNTs, would have been reflected in the best long-term stability [370]. 

Another type of CP with amino groups that have been used in combination with rGO corresponds to PoPD, as reported by Yang et al. [371]. For the synthesis of the nanocomposite called rGO-a-PoPD, they first functionalized GO with *p*-phenylenediamine, then deposited PoPDQDs (2–4 nm in diameter) by in situ oxidative chemical polymerization and carried out a final reduction stage. The incorporation of PoPD increased the surface area from 186 to 520 m^2^ g^−1^, providing abundant mesopores and avoiding the self-agglomeration of rGO, which, together with its pseudocapacitive activity, allowed reaching a capacitance of up to 420 F g^−1^, superior to electrodes without their presence. The good anchoring of PoPD on functionalized rGO was reflected in the good stability after 5000 cycles, with retention of 90% with H_2_SO_4_ as an electrolyte, improving to 94.5% when using KOH [371].

To form inherently stretchable/deformable electrodes, Wang et al. [372] synthesized a film of MWCNTs chemically crosslinked with ACM, with a final stage of electropolymerization of 1,5-diaminoanthraquinone, forming nanolayers of PDAA. On the one hand, ACM provided deformability to the electrode and on the other, the MWCNTs offered multiple pathways for electronic transport and good support for PDAA nanolayers. Depending on the amount of PDAA electrodeposited on ACM/MWCNTs, they obtained different yields. From a certain threshold, although the capacitance increased due to the greater mass of PDAA with active redox sites (up to 25 F cm^−3^), the speed capacity worsened with increasing current density, which would be explained by an excess of chains PDAA blocking spaces for fast ion access. Using ACM/MWCNTs@PDAA as anodes, since PDAA can acquire *n*-type doping, and ACM/MWCNTs@PANI nanocomposites as cathodes, they manufactured asymmetric supercapacitors with a quasi-solid polymeric electrolyte, operating stably in a potential window of 2.7 V [372]. A volumetric capacitance of up to 2.2 F cm^−3^ and a very good retention of 86% was obtained by increasing the current density 30 times. When evaluating its stability after 300 stretching cycles at 50%, a drop in its capacitance was not obtained, maintaining its low resistance to charge transfer [372]. The maximum energy density achieved was 2.14 mWh cm^−3^ to 0.021 W cm^−3^, being superior to other reports of stretchable electrodes, which include some inorganic [377,378,379,380,381,382,383,384,385,386,387] or polymeric material [324,388].

Regarding the combination of traditional CP, there are some works that illustrate the variety of nanostructures that can be achieved. The strategy of improving the yields that a metal oxide can offer through the integration of nanostructured CP can be seen in the work of Liu et al. [373]: from a biomimetic approach inspired by the morphology of some herbaceous plants called Setaria viridis, they deposited PANI nw on PPy nanolayers, both structures obtained by in situ chemical polymerization and, in turn, incorporated in nanoribbons of MoO_3_. The PPy nanolayers acted as intermediaries between PANI and MoO_3_, facilitating the electronic transfer between them and reinforcing the anchoring of the PANI nw. The latter would have allowed an easy insertion/disinsertion of H^+^ ions in abundant redox active sites, as well as a rapid transport of electrons, thanks to their relatively vertical arrangement, impacting the good speed capacity of the electrodes [373]. For a MoO_3_@PPy/PANI electrode, a high value of maximum specific capacitance (1315 F g^−1^) was obtained, with retention of 100% after 3000 cycles of operation for a symmetric supercapacitor, evidencing the high performances obtained by the synergistic combination of materials in a nanostructuring at different levels. 

In another polymer combination study, although in this case of PANI with PEDOT, Yang et al. [374] prepared hydrogels by oxidative chemical route in the presence of phytic acid to be used as electrodes in flexible solid-state supercapacitors. The hydrogels consisted of 3D nanostructures with PANI nanoparticles, some adsorbed and others embedded in PEDOT sheets. They determined that the synthesis of the hydrogel was driven by the presence of phytic acid doping PANI and, on the other hand, free phytic acid removing PSS from the PEDOT:PSS complexes used as precursors [374]. Phytic acid allowed the formation of physical crosslinks between PANI and PEDOT and changed the conformation of PEDOT from a benzenoid to a quinoid structure, which enhanced p-p-type interactions between the polymer chains [374]. Compared to PEDOT hydrogel-only electrodes, those that also contained PANI demonstrated better electrochemical performances. For the supercapacitors, an area capacitance of up to 242.2 mF cm^−2^ was obtained, demonstrating its operation when deformed, with a retention of 82.5% for 5000 cycles under a constant bending of 150°. Although it would require further optimization in terms of capacitance, for example, through other doping agents or using surfactants to vary the porosity of hydrogels, this type of research offers a viable alternative as regards the feasibility of combining CP in this class of nanostructures.

Under the approach of pyrolyzing polymeric structures to obtain materials based on doped carbon, it can be found reports that have used copolymers. For example, from the carbonization of PPy-co-PANI, Wang et al. [375] synthesized hierarchically porous CNTs, with micro-/meso-/macropores. The first polymerization stage was carried out on the surface of MnO_2_ nw (80–100 nm in diameter), which acted as templates and oxidizing agents that subsequently dissolved when reduced to Mn^2+^ ions. The amount of N reached 5.31 atomic%, benefiting electrical conductivity and ionic permeability [389]. The nanostructure symbolized as HPCNT presented a high surface area (1419 m^2^ g^−1^) to store charge by the electric double layer mechanism, reaching a capacitance of 286 F g^−1^ at 0.1 A g^−1^, higher compared to electrodes of AC. In addition, it demonstrated remarkable retentions of 71% when increasing the current density 500 times and 100% after 10,000 charge/discharge cycles [375]. In this way, a long-term structurally stable material was obtained, with adequate spaces for rapid ionic diffusion, as the current density increased. Notwithstanding the above, due to the type of charge storage mechanism, its medium-low level of capacitance is not at the level of other reports considered in this review. 

In this category of electrode materials, Karuppannan et al. [376] obtained hollow N-doped carbon spheres decorated with nanolayers of F-doped carbon (F-C) on FeCo nanocrystals. For this, aniline was copolymerized with 2-fluoroaniline by oxidative chemical synthesis on SiO_2_ templates that were subsequently removed. The pyrolysis of P(ANI-co-F-ANI) allowed us to obtain the carbonaceous structures doped with N and F on FeCo. These metallic nanocrystals, with a high electron density, would have promoted the formation of carbon nanolayers (sp^2^ hybridization) doped with F, as this element is highly electronegative [376]. In addition to the electrical double-layer mechanism, the F-C nanolayers contributed with their pseudocapacitive behavior, increasing the maximum specific capacitance from 210 to 302 F g^−1^, obtaining the best performance for 2.17% of F in atoms. The electrodes showed good capacitance retentions, with 61.4% increasing 50 times the current density and 120% after 5000 charge/discharge cycles. The operation of symmetrical supercapacitors was tested using two devices connected in series to power a blue LED light [376].

The properties offered by CPs in 2D structures, forming COFs, have been proven in supercapacitor electrodes. For example, Liu et al. [377] used two phenazine-based monomers, viz. 2-TBTBP and 3-TBQP, using a CC-coupled endogenous polymerization strategy to prepare COFs-2D type polymers, termed conjugated aromatic polymers 1 and 2 (CAP-1 and CAP-2), respectively. This approximation was carried out in solid, without the application of some noble metal catalyst, obtaining intrinsic electrical conductivities greater than when prepared by wet method [377]. Although these were 2.8·10^−5^ and 6.0·10^−5^ S cm^−1^, for CAP-1 and CAP-2, respectively, they reached a more metallic regime when doped during charge/discharge cycles under the presence of an electrolyte. The CAP-1 structure showed a beaded fiber-like morphology, with a laminar organization of ~35 nm thick and a BET surface area of 704 m^2^ g^−1^. For CAP-2, a needle-shaped fiber structure was obtained, with sheets ~4 nm thick, with a BET surface area of 594 m^2^ g^−1,^ and with higher crystallinity than CAP-1. Furthermore, CAP-2 presented better capacitance levels than CAP-1, reflected in its GCD curves, with maximums of 240 and 81 F g^−1^, respectively [377]. Unlike most CP, both CAPs would store charge through a double electrical layer over pseudocapacitance due to the lower amount of redox functional groups [377]. Despite the greater surface area of CAP-1, the better performance of CAP-2 would be related to its lower electrical resistance and its more orderly organization of pores and 2D channels, which would allow easier ionic diffusion. On the other hand, in CAP-1, ion transport could be hampered by the more amorphous 3D networks. The higher N content in CAP-2 would also play a role since it would increase wettability, facilitating contact with the electrolyte. For all the above, CAP-2 was used as a cathode in an asymmetric supercapacitor (activated carbon anode), reaching a specific capacitance of 233 F g^−1^ (at 1.0 A g^−1^), retention of ~80% after 10,000 charge/discharge cycles [377]. 

Although they are intrinsically deficient electrical conductors, 2D-CMPs based on poly (1,3,5-triethynylbenzene-ferrocene) possess structures with p-type conjugation for electron delocalization and ferrocene (Fc) groups with redox activity, which when combined with some conductive carbonaceous material such as rGO, they are good electrodes for supercapacitors, as demonstrated by Khattak et al. [378]. To anchor the CMPs, a previous functionalization of the rGO nanofilms with *p*-bromobenzene was carried out. Likewise, the 2D CMP/rGO nanocomposites showed a nanosheet morphology of ~22 nm thick. By itself, the CMPs without Fc groups offered specific capacitance levels with a maximum of 72 F g^−1^. However, the incorporation of these groups and the rGO nanosheets allowed us to achieve up to 470 F g^−1^ (933 mF cm^−3^). Furthermore, when comparing the charge transfer resistances with and without rGO, 917.5 and 8.48 Ω were obtained, respectively [378]. Therefore, this type of conjugated polymeric structure seems to be quite dependent on the incorporation of some conductivity enhancer to obtain the maximum benefit from active sites that allow store charge. Outstanding long-term stability was obtained by evaluating a symmetric supercapacitor, with retention of up to 91% after 8000 operating cycles at 5 A g^−1^. Moreover, using 1,3,5-triethynylbenzene units, Choi et al. [379] synthesized CMPs from a Sonogashira coupling with 1,4-diiodobenzene in the presence of carbon monoxide. With SiO_2_ as a template, they obtained hollow spheres of 218 ± 11 nm, with microporous nanolayers of 23 ± 3 nm thick, called H-CMP-BPPB, whose carbonyl substituents obtained thanks to the presence of CO, showed redox activity, so that stored charge by pseudocapacitive activity [379]. Considering current densities of 0.5 and 20 A g^−1^, for symmetric supercapacitors of H-CMP-BPPB, respective specific capacitances of 220 and 108 F g^−1^ were obtained, while for H-CMP without BPPB (synthesized without CO), they were 130 and 53 F g^−1^. Good long-term stability was obtained after 10,000 cycles, retaining 85% the capacitance at 6 A g^−1^, with a slight increase in RCT from 5.8 to 6.3 Ω [379]. Despite the above, the incorporation of some carbonaceous material or a strategy that allows increasing the amount of carbonyl groups could improve the charge storage capacity.

Taking advantage of the redox activity of the carbonyl and amine groups in the polymer chains, Liao et al. [379] prepared a series of CMPs forming 3D networks based on derivatives of PAQs synthesized by Buchwald-Hartwig coupling between 2,6-diaminoanthraquinone and aryl bromides. Among all the CMPs obtained, namely PAQTA, PAQCB, PAQTM, PAQSF, and PAQTB, the highest specific capacitance obtained was for the first one (576 F g^−1^, at 1 A g^−1^), with respective values of 210, 208, 208 and 165 F g^−1^, for the remaining polymers, which had no correlation with the specific area obtained for these CMPs, being the lowest for PAQTA (331 m^2^ g^−1^) and the highest for PAQTB (600 m^2^ g^−1^), which also exhibited micropores in the 0.69–1.31 nm range and mesopores of 7.5–12.5 nm. The determining factor in the best performances of PAQTA was the pseudocapacitive contribution based on its redox activity, reaching 56.5% of the total capacitance, where the remaining 43.5% corresponds to the formation of an electrical double layer [380]. In contrast, for PAQCB, PAQTM, PAQSF, and PAQTB, their pseudocapacitive contributions were 19.5%, 8.0%, 12.5%, and 10.0%, respectively. From this, it follows that pseudocapacitive activity may be more relevant than surface area optimization and electrical double-layer formation in terms of obtaining a better total capacitance. Through the redox activity of the triphenylamine (TA) and AQ units, they determined a theoretical maximum of 1440 F g^−1^. Therefore, in practice, only 40% of the potential redox activity was achieved in the PAQTA electrodes [380]. For asymmetric PAQTA//AC supercapacitors, very good retention was obtained after 2000 cycles (95.5%), operating in a potential window of 1.6 V, reaching a maximum energy density of 60 Wh kg^−1^ (at 1300 W kg^−1^), which is above other asymmetric devices that include some CP [390,391,392,393] or inorganic material [394,395,396].

Using another PAQ manipulation strategy, a work by Zhang et al. [381] is chemically synthesized in situ and carbonized on MgO nanospheres, poly(*o*-phenylenediamine-*co*-*p*-benzoquinone) to form a carbon foam doped with N and O (N, O-PC). These heteroatoms would have allowed a greater wettability of the electrodes in contact with the aqueous electrolyte (H_2_SO_4_ or Li_2_SO_4_), increasing the electrical conductivity and acting as active redox zones to store charge through pseudocapacitance. The morphology of the N, O-PC foam presented a hierarchical structure with micro-, meso- and macropores, achieving a high surface area of 1215 m^2^ g^−1^, thus facilitating ionic diffusion [381]. The electrodes reached a specific capacitance of up to 410 F g^−1^, while for the symmetric supercapacitors, with electrolytes of H_2_SO_4_ and Li_2_SO_4_, maximum values of 321 and 216 F g^−1^ were achieved, respectively. The solvated Li^+^ ions occupy more volume than H_3_O^+^ and diffuse more slowly, which could have an impact on the capacitance levels in each case. For both devices, a remarkably stable operation was obtained after 15,000 charge/discharge cycles, with retention percentages of 96–98% [381].

One class of polymeric materials that have begun to be explored for supercapacitor electrodes are triazine-based covalent frameworks (CTFs), as seen in the work of Li et al. [382]: from TCNQs monomers. They synthesized a series of CTFs, through a trimerization reaction in the presence of ZnCl_2_ and a subsequent annealing step at different temperatures (400–900 °C), which caused a rearrangement in the structure internal of the polymeric material. The annealing temperature was important for the morphology obtained and the content of N and, therefore, impact the final performance. In general, with increasing temperature, the micropore sizes and surface area were larger, while the N content decreased—cyano, graphitic, and triazine [382]. The most abrupt changes were produced when passing from 600 to 700 °C, while the best levels of capacitance were obtained for annealing at 800 °C. For this structure, the relative content of graphitic N was one of the highest, which would have improved the electrical conductivity. A maximum capacitance of 383 F g^−1^ was obtained, and excellent retention of 100% after 10,000 cycles [382]. Regarding speed capacity, it could have required greater optimization, for example, with the formation of greater mesoporosity for rapid ionic diffusion. The operability of the symmetric supercapacitors, with an IL EMIMBF4 electrolyte, was verified by powering LED lights, delivering an energy density of up to 42.8 Wh kg^−1^.

## 8. Nanostructured CP as Rechargeable Batteries

In the development of electrodes for rechargeable batteries, there is still a considerable predominance of inorganic components, which have shown good performances in energy storage capacity, long-term stability, and lower self-discharge [30,397,398,399]. However, currently, there is a whole variety of organic molecules and, to a lesser extent, electrochemically active polymers, which appear as an alternative still under development, achieving specific capacities over 200 mAh g^−1^, with levels similar to commonly used metal oxides [16,400].

The specific capacity and energy density that traditional CPs such as PANI, PPy, PEDOT, or other derivatives of PTh can develop is not at the level of inorganic materials commonly used in batteries of different ions [16,400,401,402,403,404]. This is explained by the lower amount of redox active sites to store ions, even though it can be optimized with nanostructuring. However, the use of these polymers as nanolayers, conductive binders, or interconnected networks has been shown to be beneficial for the electrical conductivity and structural stability of active materials. Regarding Li^+^ batteries, which continue to be the most reported in the literature, it has been usual to use PANI, PPy, or derivatives of PTh, in carbonized or not, as nanolayers incorporated in anodes composed of Si, metal sulfides, and mono- or bimetallic oxides. In other batteries such as Na^+^, K^+^, Zn^2+,^ or Li-S, it is more common to find polymeric nanostructures in cathodes, either in combination with carbonaceous, inorganic materials or alone. In the development of Li-S or metal-S type batteries, which are theoretically of superior energy storage [405,406], nanolayers of conductive polymers have emerged as stabilizing, conducting, and protective elements of S-containing active materials, increasing their performance.

Other polymeric structures, composed of amino or carbonyl substituents, various types of COFs, CMPs, or CAPs, have been used both in cathodes and anodes of batteries of various ions. Among these, it is possible to find 2D or 3D polymeric nanostructures with redox activity capable of storing Li^+^, Na^+^, K^+,^ or Zn^2+^ ions, without the need for the incorporation of inorganic materials. Beyond the use of the most auxiliary functions with which PANI, PPy, or derivatives of PTh have been used, in these cases, they are polymeric nanostructures in which their active redox sites are used. These different configurations with which polymeric nanostructures have been used are developed below. For more details regarding the performances of selected battery electrodes, composed of PANI, PPy, derivatives of PTh, and other nanostructures, see Table 6, Table 7, Table 8 and Table 9, respectively.

### 8.1. PANI

By using nitrogen-doped carbon (NC) coatings, better efficiencies can be achieved on Li^+^ ion battery anodes. In this sense, by pyrolysis of PANI obtained by chemical means, NC nanolayers have been obtained on LiTi(PO_4_)_3_, as reported by He et al. [407]. The nanocomposites represented as LCP were prepared with different amounts of PANI, and their yields were contrasted with a similar nanocomposite, but whose coverage was obtained by the carbonization of sucrose (LCS). The optimized version of the LCP nanocomposite showed a well-dispersed nanoparticle morphology of 50–250 nm in diameter, with less self-aggregation than LCP. The amorphous N-C coatings were of an appropriate thickness (~5 nm), allowing good electrical conductivity and easy penetration of Li^+^ ions. This factor is important since an excess of N-C, derived from an excess of PANI, is counterproductive to the electrochemical performance and can cause the obstruction of the Li^+^ insertion/disinsertion processes. The N content has also been fundamental since it added defects to the carbonaceous structure, with more channels that favored the diffusion of Li^+^ within the electrode [418]. The morphology of LCP-10 had a direct impact on the good reversibility of the charge/discharge processes, allowing an initial discharge capacity of up to 122.4 mAh g^−1^, maintaining levels higher than LCS at higher current densities. In addition, it showed a coulombic efficiency of ~100% and stability with a retention of 82.1% after 1000 cycles, being superior to [407]. 

The combination of carbonized PANI with carbonaceous structures derived from biomass has also been shown to be a functional approximation in LIBs anodes, as shown by Wang et al. [408], who used sugarcane bagasse to obtain biocarbon through pyrolysis and activation processes, with a porous 3D structure, with optimized surface area. Then, it was subjected to a nitrogen doping process, using PANI in the form of nanoneedles/nanoparticles as precursor material, synthesized by in situ chemical polymerization, ending with a pyrolysis-activation step. The nitrogen-doped nanocomposite (NSBDC) was evaluated as a supercapacitor and LIBs electrode. Compared to electrodes composed of biocarbon without PANI (SBDC) and pyrolyzed and similarly structured PANI (NPDC), those of NSBDC showed the best levels of capacitance at low and high current densities (0.1–5.0 A g^−1^) and a reversible capacity of 357 mAh g^−1^ after 200 cycles, with a coulombic efficiency of 96% [408]. This would have had a close relationship with the optimization of morphology and N content. For NSBDC, the surface area was 1939.9 m^2^ g^−1^, while for SBDC and NPDC, they were 1540.9 and 1525.4 m^2^ g^−1^, respectively [408]. In addition, NSBDC obtained a total pore volume of 1.30 cm^3^ g^−1^, with an average diameter of 3.2 nm, greater with respect to carbonized PANI (1.12 cm^3^ g^−1^ and 1.12 nm), although for the latter, the N content was higher in atomic percentage (2.3% vs. 2.1% for NSBDC). Therefore, a combination of the N content and the optimized porosity of NSBDC facilitated the entry of Li^+^ ions towards abundant sites for charge storage at the electrode/electrolyte interface [408].

Another approach for the manufacture of anodes based on carbonized PANI, where hybrid composites are incorporated, can be found in the work of Sheng et al. [409], in which it was combined with interconnected rGO fibers, covered by MnO nanoparticles. The PANI-derived NC structure consisted of 1–2 nm nanolayers deposited on the MnO nanoparticles. In addition to the electrical conductivity that the NC nanolayers could provide, they would have been fundamental in the size of the MnO nanoparticles and in the optimization of the surface area. Without its presence, the MnO nanoparticles underwent self-aggregation, increasing in size to even 500 nm, decreasing the surface area from 115 to 56 m^2^ g^−1^. Due to the N content and the optimized porosity of the nanocomposite, a higher level of specific capacity was obtained with a greater number of active sites, better long-term stability, lower internal and charge transfer resistance, and better ionic diffusion, compared to its components separately [409]. This was reflected in parameters such as the initial discharge capacity (1802 mAh g^−1^) and the reversible capacity of 904 mAh g^−1^ after 500 cycles, being higher than 508 mAh g^−1^ for the nanocomposite without carbonized NIBP.

Likewise, using the combination of MnO and NC, Zhu et al. [410] synthesized multicore nanocomposites@layer, which are described as walnut-shaped nanocapsules. From Mn_3_O_4_ nanoparticles wrapped in PANI, obtained hydrothermally, a second carbonization stage was carried out to obtain the MnO@NC nanocapsules. Similar to the previous case, the role of NC derived from PANI served to improve properties such as electrical conductivity, long-term stability during operating cycles, wettability, and ionic diffusion, in addition to avoiding self-aggregation of MnO nanoparticles [410]. The electrodes presented reversible capacities of 762 mAh g^−1^ (at 100 mA g^−1^) and a capacity of 624 mAh g^−1^ after 1000 cycles at 1000 mA g^−1^, with 100% coulombic efficiency, demonstrating a good level of even long-term energy storage. Using these electrodes as anodes and LiFePO_4_ as cathodes, flexible soft-packed batteries were manufactured, developing a reversible capacity of 60 mAh g^−1^ after 200 cycles, managing to power 34 LED lights simultaneously with a single device [410].

In order to improve the stability and conductivity in Si anodes for LIBs, the incorporation of carbonaceous materials or PANI in the form of nanolayers has proven to be a good strategy, as reflected by Zhou et al. [411]: from a hydrothermal treatment and carbonization of a melamine foam as template and CNTs, they synthesized a kind of CNT foam, on whose surfaces they deposited Si nanoparticles. In turn, on the 3D structure of CNTs/Si, they chemically deposited PANI nanolayers of ~5 nm thick, forming a foam-like nanocomposite (PANI-Si @CNTs). Hierarchically porous morphologies were observed in these, favoring ionic diffusion, where in addition CNTs and PANI acted as pathways for rapid electronic transport. Additionally, the PANI nanolayers attenuated the volumetric changes suffered by the Si nanoparticles during the Li^+^ insertion/disinsertion cycles, prevented their rapid degradation, and generated a stable SEI, which prevented direct contact between Si and the electrolyte [411]. Compared to pure Si and Si@CNTs electrodes, the incorporation of PANI enabled better electrochemical performances, achieving a reversible specific capacity of 919 mAh g^−1^ within the first 10 cycles and even 727 mAh g^−1^ after 100 duty cycles at 100 mA g^−1^.

A common strategy in Li-S battery cathodes is the incorporation of a PANI nanostructured shell to increase electrical conductivity and electrolyte permeability and prevent the dissolution of lithium polysulfides. However, depending on the materials used, it is not the only way to improve the performance of an electrode and it can coexist synergistically with other optimization strategies. In this area, Yan et al. [414] reported a 3D structure based on ethylenediamine (EDA) modified carbon nanotubes (CNTs)/S, covered with a PANI deposit. In addition to the advantages granted by the PANI nanolayers, the modification with EDA to anchor the polysulfides also helped to prevent their dissolution, and the structure of 3D networks of CNTs/S was a suitable way to give mechanical and electrochemical stability, supporting the processes volumetric expansion and increasing the kinetics of the charge/discharge processes, given the better ionic diffusion [414]. The best performances of PANI@CNTs-EDA/S were contrasted in terms of the results of cycle stability, speed capacity, and EIS spectra with respect to samples of nanoparticles of S without coverage and composites of PANI@CNTs/S without EDA [414]. On the other hand, just the presence of PANI nanolayers as a structural support to prevent the dissolution of polysulfides was not enough, reinforcing the fundamental idea of incorporating EDA molecules. In this, it is important to consider the weak binding energies between the nonpolar carbon structure and the polysulfide clusters [414]. 

The combination of PANI/CNT has also been used as stabilizing and conducting nanolayers by Wu et al. [415] on hierarchically porous spheres for the storage of S. However, in this case, PANI was used on two other occasions, the first being pyrolyzed to synthesize mesoporous carbon spheres, and second, to form microporous layers over-enveloping the spheres, obtaining a hierarchical structure. This microporous layer allowed to increase in the total volume of pores from 1.23 to 1.42 cm^3^ g^−1^, facilitating the insertion of Li^+^ ions. Both this last envelope, as that of PANI/CNT, formed a synergistic combination, which gave better long-term stability, compared to cathodes with spheres of only one coverage, delivering a capacity of 454.5 mAh g^−1^ after 500 cycles (a 2 A g^−1^) [415]. The good speed capacity, optimized with the abundant channels and the electrical conductivity of the cathodes, was reflected in obtaining specific capacities of 1371.2 and 723.6 at current densities of 0.2 and 2 A g^−1^.

Recently there have been some studies with the use of NIBP in battery electrodes of other ions (e.g., Na^+^, K^+^, Zn^2+^, etc.), although less numerous in relation to reports with Li^+^ ions. Zhang et al. [415] showed the synthesis of 600 nm PB cubes, covered by PANI nanolayers of ~20 nm, through aqueous precipitation and in situ polymerization steps, with PVP assistance to fix the polymer. The PB@PANI nanocomposites showed a core-shell type morphology and offered better electrochemical performances as cathodes for SIBs, in terms of specific capacity, electrical resistance, speed capacity, and cycle stability, compared to PB cubes without PANI and PB@PANI without PVP [415]. PANI nanolayers increased electrical conductivity and ionic diffusion while protecting PB, preventing its rapid degradation in the face of volumetric changes. Of importance was the setting of the operating window of voltage or cut-off voltage, between 2.0 and 3.6 V (vs. Na^+^/Na), since if it was reached at 4.2 V, the electrochemical activity of NIBP increased, undergoing volumetric changes that ended by fragment its structure, decreasing the stability of cycles. In the long term, there was stable maintenance of the specific capacity, obtaining 89.4 mAh g^−1^ to 2 A g^−1^ after 1000 cycles [417]. Another work on the use of PANI, but under the scope of anodes for SIBs, is that of Zhao et al. [418], who worked on the design of NiS_2_@NC@NC nanocomposites, in the shape of double-layer rods, from the precursor’s Ni(DMG)_2_ and PANI, subjected to sulfidation and pyrolysis steps. The incorporation of carbonized PANI was not only for the purpose of improving properties such as electrical conductivity, Na^+^ diffusion, and mechanical stability but also an increase in the total pore volume and in the surface area [418]. Regarding this last parameter, in NiS_2_@NC without carbonized NIBP, 9.07 m^2^ g^−1^ was obtained, while NiS_2_@NC@NC reached 25.7 m^2^ g^−1^. Likewise, the incorporation of the carbonized PANI layer improved specific capacity levels and high-speed and long-term stabilities [418]. For example, NiS_2_@NC@NC reached a remarkable capacity of 580.8 mAh g^−1^ at 0.1 A g^−1^ after 100 cycles, being much higher than NiS_2_@NC, with only 86.8 mAh g^−1^.

In the field of cathodes for K^+^ ion batteries that incorporate PANI in their structure, Gao et al. [419], by oxidative chemical polymerization, synthesized PANI nanofibers of ~80 nm in diameter, integrating them with a KPF_6_/PMMA polymer gel electrolyte. This is one of the few recent works that has directly dealt with nanostructured PANI as an active material, taking advantage of its *p*-type doping qualities. A reversible specific capacity of up to 138 mAh g^−1^ at 10 mA g^−1^, with retention of 98% after 100 cycles (at 50 mA g^−1^), was reached in the polymeric gel electrolyte. In the study, the comparison between the use of a liquid organic electrolyte and the polymeric gel was important, being more stable at high current densities and, in the long term, cells based on the latter [419]. Regarding some reports with inorganic cathodes used in KIBs, PANI’s nanofibers are above the level of some of those in terms of specific capacity, average voltage, and energy density [465,466,467,468,469,470,471,472].

In the same PANI cathode manufacturing line, although in this case for ZIBs, Liu et al. [420] have presented the electrosynthesis of this CP in the form of nanopillars, being used in flexible aqueous devices. For good adhesion of the PANI nanostructure, cellulose nanofibers were used, both to a current graphite collector and to a filter paper separator. Based on the mass of PANI, specific capacities of 203.5 and 118.7 mAh g^−1^ were obtained, for current densities of 0.5 and 16 A g^−1^, respectively. In addition, it maintained a retention of almost 100% after 1000 cycles. Considering only the PANI cathodes and for a complete cell (Zn anodes), energy densities of up to 233.4 and 175.1 mWh g^−1^ were reached, which would be at the level of some LIBs of organic electrolytes [420]. Although the stable performance was demonstrated under bending and torsion tests, it was not contrasted with PANI without nanostructuring, or the non-use of cellulose, to verify the relevance of such modifications. 

In the field of multifunctional devices, Kim et al. [421] showed the design of an aqueous ZIB-type cell integrated with a usable photosensor. Electrospinning, laser micromachining, and 3D printing techniques were combined in manufacturing. The battery consisted of an activated carbon fiber (CF) cathode, covered with high porosity PANI synthesized via in situ polymerization, a glass fiber porous separator, and a metallic Zn anode. Using GCD curves, a good speed capacity was demonstrated due to the optimal electrical conductivity of PANI and CF and the rapid ionic diffusion into the highly porous electrode [421]. The conformability, versatility, and functionality of this type of battery were tested by designing H-shaped, ring, and cap-shaped structures. In addition, a battery pack was built, connected in parallel and in series, being tested in a photosensor and in LED lights within a multifunctional device [421]. Notably, the batteries operate within a wide range of current densities (1–600 °C).

Similar to PANI, some approaches to obtaining nitrogen-doped carbonaceous materials for use in LIB electrodes are based on the pyrolysis of PPy [422,423,424,426,427,428,430]. Luo et al. [422] evaluated a nitrogen-doped (NC) carbon-encapsulated Sb nanocomposite as a storage anode for lithium and sodium ions. The nanocomposite (Sb@N-C) was manufactured by in situ carbonization and reduction in Sb_2_S_3_@PPy nanorods, previously obtained by the hydrothermal route and in situ chemical polymerization. In the core-shell type structure of the Sb_2_S_3_@PPy precursor, homogeneous polymer coatings were obtained, up to 15 nm thick. The incorporation and pyrolysis of PPy made it possible to obtain, mainly, pyrrolic (61%) and pyridine (27%) type N doping, which induce defects and active sites that allow good electrochemical performance [422]. Using metallic Li as a cathode and Sb@NC as an anode, the retention of capacity at 92.7% after 300 cycles and 100% coulombic efficiency can be highlighted, which would be explained by the good maintenance of the 1D structure of the nanorods and the coverage of NC derived from PPy. 

Under a similar strategy, Sun et al. [423] have reported the manufacture of MoS_2_ nw with hierarchical structures composed of intercalated nanofilms and covered by nanolayers of NC derived from PPy, through a method of interfacial synthesis and pyrolysis. The hierarchical structure of the nw, constituted by numerous exposed nanofilms with channels for the access of Li^+^ ions, together with the better electrical conductivity and mechanical stability offered by the NC nanolayers, were key factors for their performance as anodes of LIBs [423]. It should be mentioned that the NC nanolayers were well anchored to the MoS_2_ nw through CO-Mo covalent bonds, maintaining the shape of these nanostructures and avoiding material degradation under the anode operating cycles, with good retention of specific capacity. Reversible capacities of 600 and 453 mAh g^−1^ were achieved, at current densities of 5 and 10 A g^−1^, respectively, with 86.7% retention after 500 cycles. Compared to pure MoS_2_, nested MoS_2_/NC nw demonstrated better charge storage, even at high current densities, greater long-term stability, lower RCT, and better ionic diffusion, demonstrating the benefits involved in incorporating NC nanolayers [423]. Although these are not active materials in the storage of Li^+^ ions, they do act as auxiliary components that also have a significant impact on performance. 

### 8.2. PPy

Using the same materials but in a different morphology is the work of Wang et al. [424], where they synthesized MoS_2_@NCnanospheres, ~100 nm in diameter, to be used as anodes in LIBs. In this case, a method based on polyoxometalate, phosphomolybdic acid (PMO), was used as a source of Mo and initiator of the oxidative chemical polymerization of pyrrole monomers. The nanocomposites thus obtained were subjected to sulfurization and carbonization processes, obtaining MoS_2_ nanosheets encapsulated within NC coatings. Its high degree of graphitization, together with N-doping, was important for electrical conductivity [424]. The manufactured anodes demonstrated an initial discharge capacity of 1344 mAh g^−1^ (75% coulombic efficiency), reversible capacity after 100 cycles of 1119 mAh g^−1^ (at 100 mA g^−1^), being at higher levels relative to MoS_2_ (~300 mAh g^−1^). Other optimized parameters were its capacity at higher current densities, the ionic diffusion coefficient, and the resistance to charge transfer, with values of 4.14·10^−13^ cm^2^ s^−1^ and 57 Ω, respectively (8.21·10^−14^ cm^2^ s^−1^ and 191 Ω, for only MoS_2_). In this, the formation of nanospheres with better porosity and MoS_2_ nanosheets in their interior was fundamental, where the inclusion of NC was important for its stability, conductivity, and ionic permeability [424]. Without resorting to the calcination of PPy in the form of nanolayers on MoS_2_, good yields can also be obtained in LIBs anodes, according to that reported by Xie et al. [425]. Combining hydrothermal methods and in situ chemical polymerization, they synthesized nested MoS_2_@PPy microspheres with diameters in the 200–300 nm range. Within the microspheres, MoS_2_ exhibited a nanofilm morphology, and PPy formed uniformly distributed porous nanolayers, preventing the dissolution of MoS_2_, increasing the electrical conductivity, and allowing the insertion of Li^+^ ions, optimizations that were reflected in some parameters evaluated. The initial charge/discharge capacities for the MoS_2_ and MoS_2_@PPy microspheres were 1309/738 and 1427/1127 mAh g^−1^ (at 200 mA g^−1^), with better speed capacity for the second. In addition, in the long term they exhibited respective capacities of 305 and 1012 mAh g^−1^, after 200 cycles [425]. 

The use of macroporous hollow nanostructures in battery electrodes is an approach that has motivated Xing et al. [426] for the manufacture of hedgehog-shaped hollow TiO_2_-anatase spheres (HUTS) covered with PPy-derived NC. With steps of dissolution and recrystallization of TiO_2_ spheres, hydrothermally, macroporous HUTS spheres (~500 nm in diameter) were obtained, composed of TiO_2_-anatase nanorods, in which PPy nanolayers (10–20 nm thick), via in situ chemical polymerization, subjecting to a final calcination stage at 700 °C. TiO_2_-anatase nanocrystals have the {001} family of crystallographic planes, particularly electroactive, which allow rapid insertion/disinsertion processes of Li^+^ cations. However, they have low electrical conductivity and can be sprayed during charge/discharge processes [426]. This has been remedied with the NC nanolayers, which even increased the total volume of the pores, facilitating the access of Li^+^ cations to the active sites of TiO_2_-anatase. The above optimizations were correlated with the increase in capacitance of HUTS@NC relative to HUTS [426]. For example, the reversible capacities after 200 cycles were 164.2 and 91.2 mAh g^−1^ (at 5 °C) for HUTS@NC and HUTS, respectively. Moreover, in terms of speed capacity, as current density increased, the drops in specific capacity were lower in HUTS@NC. The ionic diffusion coefficient for Li^+^ was four times higher for HUTS@NC, and the charge transfer resistance was less than half with respect to HUTS [427]. This highlights not only the importance of porosity and nanostructuring, but also the stability and electrical conductivity that the NC nanolayers added.

The use of calcined PPy nanocoatings has also been tested in LIBs anodes composed of a MOF-Co, obtaining very good electrochemical and structural performances. Xiao et al. [427] deposited PPy chemically on MOF-Co particles synthesized in GO nanosheets to then carry out a calcination step at 350 °C in the presence of air, producing nanocomposites (GCP350). In this last process, the presence of PPy allowed the pulverization of the encapsulated MOF-Co particles, generating nanocrystals of ~5 nm. On the other hand, without PPy, the MOF-Co particles were oxidized, transforming into Co_3_O_4_, with less energy storage capacity. The GCP350 anodes developed specific capacities of 1301 and 596 mAh g^−1^, at respective current densities of 0.1 and 20 A g^−1^, with retentions of 92.9% and 98.6% after 2000 cycles at 5 A g^−1^ and 10 A g^−1^, respectively [427]. The MOF-Co nanocrystals allowed a structure with a greater surface area with more active sites, in which the prior incorporation of PPy was important. Regarding structural stability and good electrical conductivity, the presence of the interconnected nanolayers of NC derived from PPy and the leaves of G was fundamental.

To increase the stability and electrical conductivity in active materials of Li-S battery cathodes, the strategy of incorporating nanostructured PPy has become common [422,431,432,433,434,435,473]. Song et al. [431] deposited PPy nanolayers on CoS, 10 to 40 nm thick, by means of a vapor phase chemical polymerization strategy, obtaining cubes of CoS@PPy/S. The synthesis of CoS with hollow structures was carried out from a Co-based MOF called ZIF-67, which would have allowed an abundant number of active sites and a good resistance to volumetric deformation during the operating cycles. However, the incorporation of nanolayers improved cycle stability and performance at high current densities, preventing the dissolution of the polysulfides formed in the cathode while providing adequate porosity for the insertion of ions [431]. For example, when evaluating in the long term, the incorporation of PPy nanolayers allowed increasing in the retention in specific capacity from 44.3% to 74.3% after 500 operating cycles. Even upon increasing the current speed from 0.2 C to 4 C, the capacity dropped from ~1000 to 450 mAh g^−1^. Instead, without PPy, the drop was up to 234 mAh g^−1^. Moreover, Jiang et al. [432] synthesized cathodes based on Zr MOFs (PCN-224) with interconnected pores, subjected to sulfidation and covered with PPy nanolayers (45–50 nm thick). In addition to protecting the polysulfides stored in the cathode and avoiding the loss of performance, the incorporation of PPy increased the electrical conductivity from 5.7·10^−5^ to 6.7 S m^−1^. Notwithstanding the foregoing, the type of MOF used was important, obtaining better performance for PCN-224, with more surface area and interconnected pores, compared to MIL-53 and MIL-101 [432]. To mention one parameter, the PPy-S cathodes in PCN-224 showed a capacity of 780 mAh g^−1^ after 400 cycles at 5.0 °C. On the other hand, PPy-S in MIL-53 did not exceed 600 mAh g^−1^. Therefore, it was the synergistic combination of PPy and PCN-224 which made it possible to obtain a cathode with long-term stable operation and under high current speeds, with a more auxiliary role by the polymer.

Regarding the combination of PPy with metal oxides, Li et al. [433] synthesized and studied the electrochemical yields of cathodes made up of hollow spheres of MnO_2_, covered by PPy nanolayers, inside which they housed S nanoparticles. In these nanocomposites, the polar structure of MnO_2_ and the PPy covers would have alleviated the volumetric changes inside the S nanoparticles and prevented the rapid dissolution of the polysulfides formed. Furthermore, the PPy nanolayers, in turn, composed of nanoparticles, provided sufficient pathways for electron transport during charge/discharge cycles [433]. The cathodes presented a specific capacity of up to 1488.1 mAh g^−1^ at 0.1 °C and even 736.7 mAh g^−1^ at 1 C. In relation to their cycle stability, they suffered a decay of up to 55% of the initial capacity after 200 cycles at 0.15 °C, being higher than what was achieved without the PPy nanolayers. Even longer, after 500 cycles at 0.5 °C, they retained ~70% of the initial capacity, not observing lithium polysulfides on the surface of the spheres [433]. With a similar structure, Ansari et al. [434] synthesized submicron spheres of S, doubly encapsulated with MnO_2_ and PPy. During the encapsulation stage with PPy, a FeCl_3_/methyl orange template was used and subsequently removed. This made possible a homogeneous growth of the PPy nanolayer through oxidative chemical polymerization. The S@MnO_2_@PPy (or SPPyMnO_2_) cathodes did not need binders, allowing a coulombic efficiency of up to 98%, operating over 500 cycles. Comparing the specific capacities after 75 cycles at a C/5 speed, the cathodes of pure S, S@PPy, and S@MnO_2_@PPy gave values of 238, 936, and 1054 mAh g^−1^, respectively [434]. In addition, in relation to the theoretical capacity value (1672 mAh g^−1^), the cathodes of pure S and S@MnO_2_@PPy, reached 81.7% and 47%, and when evaluating the self-discharge after 168 h of inactivity, capacity only decreased by 9% for the latter (34% for pure S) [434]. 

The combination of PPy with ferrous materials has also been used in composite cathodes with core-shell morphologies. Lu et al. [435] manufactured flexible electrodes with good electrochemical performances based on encapsulated spheres with activated carbon fiber centers and Fe_3_O_4_ coatings with pyrolyzed PPy. They demonstrated the good synergistic combination between the layers of NC derived from PPy and Fe_3_O_4_ to retain the polysulfides by chemical adsorption, avoiding the loss of active material, which was reflected in the long-term stability. Comparing cathodes of type S@AC and S@NC@AC, with capacity retentions of 26% (200 cycles) and 48% (450 cycles), respectively, those of S@Fe_3_O_4_-NC@AC showed retention of 70% after 1000 cycles, with 100% coulombic efficiency. Likewise, it delivered better speed capacity compared to the other cathodes, maintaining levels between 1316 and 531 mAh g^−1^ for 0.1 and 4 °C, respectively [435]. On the other hand, Liu et al. [473] used Fe_3_O_4_ nanospheres as removable templates and added Fe^3+^ cations as oxidants to synthesize hollow PPy nanospheres hosting S (PHNS@S). The layers of these nanospheres, with thicknesses of ~6 nm, showed a rough and porous morphology, allowing a good diffusion of Li^+^. In addition to electrical conductivity, the PPy nanospheres functioned as protective traps for the lithium polysulfides formed during discharge, preventing their loss and achieving specific capacities of 1074.2 (initial) and 781.2 mAh g^−1^ (after 100 cycles) at 0.5 °C.

Regarding the use of PPy in SIBs [422,436,437], one can return to the article by Luo et al. [422], where they synthesized the nanocomposite symbolized as Sb@N-C. When evaluating its storage capacity for Na^+^ ions, retention of ~69.6% was obtained after 200 cycles, being less than the case of lithium storage, finding an explanation in the decomposition of the electrolyte, formation of an SEI film (solid electrolyte interface) and the irreversible reaction between Na^+^ ions and residual functional groups present in NC [422]. Despite this, even at 3000 cycles, a capacity of up to 345.6 mAh g^−1^ was obtained, being at an equal or higher level, with respect to other anodes reported for SIBs composed of Sb/carbonaceous materials [474,475,476,477,478,479,480].

For use in SIB anodes, cobalt sulfides (Co_1−x_S) have been studied as active materials, but their performance can be improved by adding PPy-derived NC coatings. Dong et al. [437] prepared Co_1−x_S spheres (1–2 µm) by solvothermal route, to which they carried out three subsequent alternative treatments: calcination at 350 °C; deposition of PPy nanolayers and calcination at 350 °C before and after adding PPy. In the second case, a set of ultrafine Co_1−x_S nanoparticles embedded in the porous NC nanolayers (Co_1−x_S/C) was obtained. On the other hand, in the third case, the morphology was of nanoparticles, totally covered by NC (Co_1−x_S@C). When evaluating Co_1−x_S/C as an anode in SIB-type cells, the best electrochemical performances were achieved compared to Co_1−x_S@C and only Co_1−x_S, with a reversible specific capacity of up to 513.5 mAh g^−1^ (at 100 mA g^−1^), and even 420 mAh g^−1^ after 120 cycles of operation at 500 mA g^−1^. On the one hand, the porous NC nanolayers, forming conductive hollow spheres, offered good structural support to the Co_1−x_S nanoparticles, and, on the other, the nanostructuring optimized the amount of Co_1−x_S active sites in contact with the electrolyte to store sodium ions [436]. 

Although they are not very abundant, there are recent records of the use of PPy in cathodes of ZIBs. Wang et al. [438] manufactured a flexible, wire-shaped, shape memory property, Zn-ion rechargeable battery using flexible Nitinol (NT) and stainless steel (SS) substrates. The active materials were MnO_2_ nanocrystals and a PPy nanolayer, both electrochemically synthesized, and a gelatin-borax gel phase polymer was used as the electrolyte. The MnO_2_/PPy@SS nanocomposite was used as the cathode, and Zn nanoplates as the anode. Compared to an electrode without PPy nanolayers, the one based on MnO_2_/PPy@SS exhibited better cycle stability, with retention of 74.2% after 850 cycles, faster charge/discharge processes due to better electrical conductivity, and better interfacial adhesion to the substrate [438], which demonstrates the mechanical and electrical benefits of using CP coatings such as PPy, although it does not directly contribute to the specific capacity of the cathode. Good electrochemical performance under mechanical deformation of the flexible battery was demonstrated, with specific capacity retentions up to 90° of bending and for even 500 bending cycles of 0–45°, reaching 79% and 88%, respectively. In addition to this functionality under deformation, the recovery of shape in 6 s stands out, which is not necessarily achieved with other types of flexible devices. Shape memory behavior was demonstrated, returning to its initial state after submerging in water at 45 °C and with retention of capacitance after five bending cycles [438]. 

Another novel case corresponds to that of Zhu et al. [440] that electrochemically layer by layer manufactured MnO_x_/PPy cathodes used in photoluminescent Zn^2+^ microbatteries, interdigitated on flat PET substrates. The MnO_x_/PPy cathodes presented morphologies described as nanoclusters with abundant porosity, while the photoluminescent property was acquired by adding CdTe-QDs to the gelatin and borax-based electrolyte. Thus, in the area of microbatteries, the one in this study achieved a higher storage level than other reports [481,482,483,484], with an energy density of up to 21 mWh cm^−3^. In addition, the operation of an arrangement of these microbatteries was demonstrated, acting dually as a power supplier and color filter (green, yellow, and red).

### 8.3. PTh and PEDOT

Because PTh is a polymer that can be *n*-doped and has a reversible redox behavior, it can be considered a valid material for use in lithium battery anodes. However, the number of reports of PTh in batteries is scarce due to the poor performance in terms of the number of active redox sites, cycle stability, and speed capacity. A rational design based on PTh and derivatives can allow better electrochemical performances, as shown by a study by Zhang et al. [485], who carried out the chemical design of microporous CP such as poly (3,3’-bithiophene) (P33DT) and their evaluation as Li^+^ ion battery anodes. Unlike the linear chains of PTh, P33DT presented an amorphous 3D structure with highly interconnected chains and abundant microporosity, forming nanoparticles and nanosheets. When comparing the BET surface area between PTh and P33DT, 13 and 696 m^2^ g^−1^ were obtained, respectively, confirming the optimization achieved for the interconnected polymer and its consequent advantage as an active material for the Li^+^ storage anode. This could be confirmed by cyclic voltammetry, obtaining a greater response for P33DT, given the existence of more active redox sites to store Li^+^ ions. Furthermore, specific capacities of 1403 (first cycle) and 367 mAh g^−1^ (at 5000 mA g^−1^) were obtained for P33DT and of 745 (first cycle) and 141 mAh g^−1^ (at 3000 mA g^−1^) for PTh, as well as better cycle stability for P33DT [485]. Particularly, with P33DT, specific capacities were obtained superior to other reported CMPs anodes [486,487] and to the level of some carbonaceous and inorganic types in LIBs [488]. These parameters reflected the better diffusion and storage capacity of Li^+^ and greater structural stability of P33DT, supported by its higher porosity, number of active sites, electrical conductivity, and lower level of LUMO [489]. 

In the same study, the influence of other microporous polymers, based on thiophene units, was evaluated. Depending on the central units used, namely thiophene, benzene, and pyrene, the corresponding polymers were obtained (PTTT, PTTB, and PTTPy). The stiffer the central unit (thiophene < benzene < pyrene), the less the amount of thiophene in the polymer chains, and the greater the surface area. When evaluating the specific capacity, the electrochemical performance followed the order PTTT > PTTB > PTTPy, that is, in direct relation to the amount of thiophene and inversely to the surface area [485]. Regarding cycle stability, this was in the order PTTPy > PTTB > PTTT, which is explained by the greater porosity and surface area, greater rigidity of the central unit, and greater distance between doped units of bitiophene [485]. However, considering cycle stability and specific capacity, P33DT proved to be the best candidate as an anode for a lithium battery. All of this accounts for the influence of the different structural and molecular factors on mechanical and electrochemical performance and that exceeding one aspect may compromise a negative impact on another.

To alleviate the degradation and performance of Si anodes used in LIBs, there are strategies based on the use of PEDOT as a protective and electrically conductive layer. As studies by Zeng et al. [440], additional modifications of this polymer have made it possible to obtain polymeric binders-coatings, which have exhibited a conductive duality of both electrons and Li^+^ ions. By means of entanglement, chemical reduction, and electrostatic self-assembly, they managed to combine the ionic conductors PEO and PEI with the polymeric complex PEDOT:PSS. A binder polymer with a higher ionic diffusion coefficient (4.0·10^−8^ cm^2^ s^−1^) and electrical conductivity (271 S cm^−1^) was obtained, with respect to the widely used CMC (2.8·10^−8^ cm^2^ s^−1^ and 3 S cm^−1^, respectively) [440]. The Si particles, with an average diameter of 180 nm, were covered by a nanolayer of the polymeric binder, forming the nanocomposite c-PEO-PEDOT:PSS/PEI/Si. When evaluating its performance, better diffusion of Li^+^ ions and lower charge transfer resistance were achieved, with respect to other samples of PEDOT:PSS/Si and c-PEO-PEDOT:PSS/Si, demonstrating the beneficial synergy that occurs by the combination of the three polymeric systems used. The c-PEO-PEDOT:PSS/PEI/Si electrode demonstrated a good specific capacity, in which the polymeric binder system did not contribute and, compared to the rest of the aforementioned samples, delivered superior cycle stability (2027 mAh g^−1^, after 500 cycles). This demonstrated the best mechanical robustness of the c-PEO-PEDOT:PSS/PEI cover-binder due to electrostatic interactions and chemical bonds between the different polymers [440]. Although this type of multifunctional polymeric binder system does not directly contribute to charge storage, it can improve auxiliary aspects related to stability and kinetics in battery anodes. Thus, the versatility with which PEDOT can be used and enhanced, in terms of its combination with other polymers and the role it can provide, has been demonstrated.

In the same field of the synthesis of conductive binders, Wang et al. [441] showed the preparation of a highly conductive, ductile, and deformable binder, denoted CG, based on PEDOT:PSS chains cross-linked with D-sorbitol and AAV, surrounding Si nanoparticles in LiBs anodes. From the point of view of synthesis, regarding the GC components, it was remarkable the possibility of mixing them homogeneously in water with the Si nanoparticles without the need for the use of other toxic solvents. The volumetric and longitudinal deformations of CG reached 250% and 400%, respectively, without a significant loss of conductivity nor exhibiting structural fracture [441]. The Si nanoparticles as active materials achieved good anchoring to each other and to the current collectors, thanks to the incorporation of CG, accommodating in a good way to volumetric changes in the charge/discharge cycles, avoiding rapid degradation. On the other hand, auto aggregation between nanoparticles was prevented, which made it possible to maintain a high surface area, positively impacting the number of active sites to store charge, coulombic efficiency, and cycle stability [441]. The good adhesion of the CG layers to the nanoparticles would have occurred through covalent bonds between the polymer chains with SiO_2_. In addition to its stability against deformation, the 3D lattice of the CG chains would allow a fast and easy transport of electrons through multiple pathways. In the electrical conductivity exhibited by CG, up to 7 S cm^−1^, the addition of D-sorbitol was important, which acted as a crosslinker and dopant, increasing this parameter by four orders of magnitude [441]. To achieve the advantages described, only 10% by mass of CG was enough, the remaining percentage being for the nanoparticles, which was of importance, considering that only the latter are electrochemically active to store Li^+^. Overall, when comparing anodes with PVDA, CMC, and only PEDOT:PSS binders, those with CG demonstrate better mechanical and electrochemical properties, with an initial capacity of 3788 mAh g^−1^ and reversible capacity of 1500 mAh g^−1^, at 840 mA g^−1^ after 700 cycles [441].

A more complex type of anode in terms of components and 3D network morphology is found in studies by Kwon et al. [442], using a commercial water-soluble class of carboxylated polythiophene (PPBT) to maximize electron transport within the electrodes. Within a 3D network of FWCNTs, monodisperse spheres of Fe_3_O_4_ were incorporated as Li^+^ storage active materials, covered by PEG, and combined with CB nanoparticles. In this mix of materials, the PPBT chains served as a bridge for the electronic transport between the networks of FWCNTs, and the Fe_3_O_4_@PEG spheres, something fundamental for the performance of the anodes in the charge/discharge processes, reducing their resistance. Internal [442]. The PPBT chains maintained a good connection between FWCNTs and Fe_3_O_4_@PEG, on the one hand, through their conjugated central structure, developing interactions of the p-p type with the FWCNTs and on the other, with their carboxylate substituents, forming H bridges, with hydroxyl end groups on the surfaces of the Fe_3_O_4_@PEG spheres. The foregoing would also have had an impact on the structural stability of the anodes, fixing the various components in a good way, and avoiding a rapid degradation of the active materials. Compared with anodes of Fe_3_O_4_ spheres/CB/PVDF nanoparticles and Fe_3_O_4_-PEG spheres/CB/PPBT nanoparticles, the nanocomposite named FWNT networks showed better levels of specific capacity, speed capacity, long-term stability term, and lower electrical resistance [442] demonstrating the merits of using a polymer derived from PTh with the appropriate substituents to obtain a particular functionality, in this case, PPBT as an electrical conductor and interconnector. The use of a 3D network of FWCNTs of abundant porosity and well-fixed to the CP chains also stands out.

Another derivative of PTh most used in areas such as photovoltaics is P3HT, which was used as a conductive binder/adhesive, as reported by Lai et al. [443]. In this case, they manufactured anodes for LIBs composed of NCA, covered by P3HT nanolayers in combination with CNT, offering multiple pathways for rapid electronic conduction, protecting the dissolution of NCA in the electrolyte against charge/discharge cycles and with porosity for intercalation/deintercalation of Li^+^ cations. In this regard, good electronic and ionic conductivities would have been possible due to the electrochemical doping of P3HT in the potential range in which NCA is electrochemically active. Compared with anodes based on NCA/PVDF, better levels of specific capacity were obtained at different current densities and also better long-term stability [443]. For example, when using P3HT-CNT, it was possible to reach a capacity of 83 mAh g^−1^ at 32 °C, whereas, for NCA/PVDF, it was almost 0 mAh g^−1^. In relation to inorganic coatings reported for NCA, such as Co_3_(PO_4_)_2_ [489], LiAlF_4_ [490], AlPO_4_ [491], and FePO_4_ [492], among others, that of P3HT-CNT has allowed relatively equal or higher yields in terms of cycle stability. Using materials of the same class but in the design of LIBs cathodes, Chae et al. [444] showed the synthesis of conducting P3HT nanolayers on Ni-enriched NCM particles (NCM811), which is known as an artificial cathode-electrolyte interface, by means of a spin-coating method. In this way, it was tried to stabilize the active materials of the cathode, avoiding rapid loss during the operating cycles. This was contrasted against pure NCM811 cathodes when evaluating the retention of specific capacity after 50 cycles, obtaining percentages of >91.3% and 84.2% in the respective cases with and without P3HT [444]. Additionally, the nanolayer would have prevented further decomposition of the electrolyte, which could have occurred due to the reaction of EC and EMC with Ni^4+^ cations on the surface of NCM811. The best performances in terms of specific capacity and long-term stability, even at high temperatures (60 °C), were obtained for P3HT layers of lower molecular weight within the range used (36–90 kDa), demonstrating the highest crystallinity, hardness, and lower electrical resistance [444].

Beyond the use of PTh derivatives in LIBs, primarily as conductive binding coatings, Lee et al. [445] reported the design of cathodes for SIBs composed of M-NVP and thin layers of PEDOT. The chemical polymerization of EDOT occurred simultaneously with a process of deintercalation/intercalation of Li^+^ ions on the surface of Li_3-x_V_2_(PO_4_)_3_ particles. The core-shell nanocomposites from M-NVP@PEDOT exhibited particle morphology with diameters of ~200 nm, and the CP nanolayers exhibited uniform thicknesses of ~5 nm. In general, compared to pristine M-NVP cathodes, M-NVP@PEDOT-based cathodes showed specific capacities up to four times higher, operating for 500 cycles [445]. Even at a current rate of 10 C, M-NVP@PEDOT and pristine M-NVP delivered respective specific capacities of 109.8 and 12.7 mAh g^−1^, demonstrating the superior speed capabilities of the former. In this way, the benefits granted by PEDOT are corroborated, with porosities that made possible the diffusion of Na^+^ ions, avoiding the dissolution of M-NVP and improving electrical conductivity.

Among the alternatives as cathodes in Zn^2+^ ion batteries are materials such as a, b, g, d-MnO_2_, V_2_O_5,_ or Prussian blue analogs, which undergo degradation and dissolution. For this reason, CP and PEDOT are used as protective, binder, and electrically conductive layers in this type of cathode. For example, Xu et al. [446]] synthesized VS_2_-derived V_2_O_5_ nanosheets on carbon fiber (CC) cloth, which were then covered with a ~5 nm thick PEDOT electrodeposit. The nanocomposite represented as V_2_O_5_@PEDOT/CC presented a core-shell type morphology, with interconnected nanosheets of V_2_O_5_@PEDOT anchored on the CC fibers, which allowed obtain of flexible cathodes. With the V_2_O_5_@PEDOT/CC cathode, a metallic Zn anode, and Zn electrolyte (CF_3_SO_3_)_2_, a battery was built that reached an initial specific charge/discharge capacity of 448.2/343.7 mAh g^−1^, with a good capacity of speed [446]. Furthermore, after 1000 cycles, its reversible capacity stabilized at a value of 223.6 mAh g^−1^ (at 5 A g^−1^), with a coulombic efficiency of ~100%. All these parameters were superior with respect to other types of cathodes based on V_2_O_5_ and V_2_O_5_/CC powder without PEDOT [446]. The reasons behind the performance are explained by factors such as the structure of the nanosheets and the porosity of PEDOT, which allowed an abundant and easy insertion/disinsertion of Zn^2+^ ions; the good stability and support provided by PEDOT, preventing the collapse of V_2_O_5_ in charge/discharge processes, with their corresponding volumetric variations and the better electrical conductivity provided by PEDOT, which had an impact on the kinetics of the cathode. In addition, maximum energy and power densities of 243.3 Wh kg^−1^ and 18,000 W kg^−1^, respectively, were reached, being at a higher level in relation to other purely inorganic cathodes reported for ZIBs, such as H_2_V_3_O_8_ [493], d-MnO_2_ [494], a-Mn_2_O_3_ [495] and LiV_3_O_8_ [496]. 

With the same role for PEDOT but with a ZnMn_2_O_4_ cathode with oxygen vacancies (OD-ZMO), Zhang et al. [447] showed the optimization of a cathode used in a flexible solid-state ZIB. The preparation involved an annealing step in obtaining OD-ZMO, and then PEDOT nanolayers with thicknesses of 5–9 nm were electrodeposited, forming the OD-ZMO@PEDOT core-shell nanocomposite on a conductive carbon fiber substrate. When evaluating a cell composed of metallic Zn anode, cathode, and electrolyte, OD-ZMO@PEDOT and 1 M ZnSO_4_, respectively, a maximum capacity of 221 mAh g^−1^ was reached, with high reversibility and lower electrical resistance, in comparison with cathodes without PEDOT coverage or without oxygen vacancy treatment [447]. Indeed, these two factors have led to better returns. Both PEDOT and the oxygen vacancies would have allowed a greater and faster insertion/disinsertion of Zn^2+^ and H^+^ ions and a lower resistance to charge transfer. The maximum specific capacity achieved was found at higher levels with respect to other cathodes based on inorganic materials, reported for ZIBs, such as NiHCF [497], Na_0.6_V_6_O_15_ [498], ZnMn_2_O_4_ [499], Co_3_O_4_ [500], to mention some. Regarding the stability of OD-ZMO@PEDOT, a retention of up to 93.8% in capacity was obtained after 300 charge/discharge cycles, which could be due to the support of the PEDOT coverage, which would not only prevent the collapse of OD-ZMO during the insertion/disinsertion of Zn^2+^ but also, it would provide elasticity to changes in volume in this process [447]. 

Additionally, when evaluating flexible solid-state batteries (gel electrolyte based on PVA), their performance was not impaired under torsion and bending conditions, and maximum energy and power densities of 273.4 Wh kg^−1^ and 20.1 Wh cm^−3^ were obtained, respectively [447]. Although PEDOT does not directly contribute to the energy storage capacity in this type of cathode, it is essential for cycle stability and better kinetics.

Xie et al. [450] tested for the first time on battery cathodes the conjugated polymers PPTO and PEPTO, with four carbonyl groups per PTO monomer, were chemically synthesized as nanoparticles. The complete utilization of the redox active centers in the PTO monomers is attributed to the high stability of HOMO and the aromaticity achieved by reduction, forming tetravalent anions. For PPTO and PEPTO, respective initial discharge capacities of 180 and 185 mAh g^−1^, and respective reversible capacities of up to 234 and 244 mAh g^−1^, were obtained at 20 mA g^−1^. Their differences were more noticeable at higher current densities, where PEPTO presented a much higher speed capacity compared to PPTO. Likewise, with PEPTO, better long-term stability, energy density, and lower resistance to charge transfer were achieved (210 Ω vs. 391 Ω for PPTO). Related to these better yields would be the presence of triple C-C bonds between PTO units within the PEPTO chains [450]. As examined in DFT studies, this increased the planarity of the PEPTO polymer chains, facilitating p-p type conjugation and improving electrical conductivity. Furthermore, by examining trimers, the bandgap energies for PPTO and PEPTO were theoretically calculated to be 3.03 and 2.65 eV. A lower bandgap energy facilitates the transfer of electrons and Li^+^ cations in the polymer chains, which translates into a better speed capacity to operate at high current densities and lower charge transfer resistances [450]. These relationships between the inherent electronic properties of polymer chains and their performance as organic electrodes in batteries, together with the assistance of theoretical studies using DFT, may offer an interesting and meaningful perspective in the search and prediction of better CP-based nanostructures.

Otteny et al. [451] have worked on the basis of PVMPT, presenting a modification of this by crosslinking (X-PVMPT) in order to avoid rearrangement and dissolution processes, which are detrimental to the use of the theoretical capacity of charge storage (112 mAh g^−1^). Through images obtained by SEM, an arrangement of X-PVMPT nanoparticles was observed, with a certain degree of agglomeration. When tested as an active cathode material, X-PVMPT showed a specific capacity at the theoretical level, good speed capacity, although lower than PVMPT, and excellent cycle stability (95% retention for 1000 cycles), operating at a stable potential of 3.5 V (vs. Li/Li^+^). In addition, it was tested in LIBs cells composed of a 70% by mass X-PVMPT cathode and Li_4_Ti_5_O_12_ (LTO) anodes, reaching a specific capacity of 87 mAh g^−1^, with retention of 96% after 100 cycles [451]. In general, the performance of X-PVMPT was superior to PVMPT due to the modification based on crosslinking, which avoided the interactions of the p-p type between the phenothiazine groups, which tends to decrease the capacity to store charge due to lower availability of redox-active sites [451]. 

### 8.4. Copolymers and Derivatives of CP

Generally, by adding binders or additives that offer stability to the active materials of the electrodes, the internal electrical resistance increases. In order to overcome the performance limitations imposed by such substances, Liu et al. [452] synthesized conductive PFA additives by in situ polymerization of furfuryl alcohol (FA) monomers, forming nanometric layers in electrodes composed of olivine-LiFePO_4_ (LFP) particles. An important advantage of these PFA additives was the optimization of three functions, namely, improving electrical conductivity, assisting in the diffusion of Li^+^ ions, and giving mechanical integrity to the active materials of the electrode. The long aligned ordered, and conjugated PFA chains allowed an electrical conductivity of 3.72·10^−5^ S cm^−1^ in the LFP-PFA electrodes, in contrast to those based on LFP-PVDF, where no electrical conductivity was detected [452]. The better conductivity of Li^+^ in LFP-PFA with respect to LFP-PVDF was corroborated by EIS, obtaining a higher ionic diffusion coefficient and, on the other hand, through DFT calculations, a lower diffusion energy barrier was determined, where the O atoms of the furfuryl units [452] would have been fundamental. Regarding charge storage, the LFP-PFA and LFP-PVDF electrodes presented reversible specific capacities of 160.3 and 75.7 mAh g^−1^, respectively, at 0.1 C. In addition, LFP-PFA showed better energy and power densities than LFP—PVDF [452]. With these electrodes, complete batteries were manufactured, with graphite anodes, in a soft-pack format with areas of 20 × 4 cm^2^. This class of additives could be tested with different combinations of materials to improve the performance of electrodes in energy storage devices.

Regarding the study of LIBs that works stably below 0 °C, there have been reports based on the improvement of the electrodes due to the use of protective coatings [501,502], ionic doping [503,504,505], or the use of additives in electrolytes [506,507]. Sun et al. [464] reported the design of core-shell nanocomposites, composed of a protective and conductive layer of polyphenylene (~10 nm thick) on the surface of ~3 µm particles of LiNi_0.6_Co_0.2_Mn_0.2_O_2_ (LNCM-3). The synthesis of LCNM-3@polyphenylene was carried out by spontaneous chemical polymerization, starting from LCNM-3 and C_6_H_5_N_2_^+^BF_4_^−^. When evaluating the material as a cathode in a LIB, better performance was demonstrated, even −20 °C, with respect to LCNM-3 without coverage with CP [453]. LCNM-3@polyphenylene exhibited an electrical conductivity of 1939 S cm^−1^, discharge capacity up to ~148 mAh g^−1^ (at 0.1 °C), rate-down capacity to ~105 mAh g^−1^ (at 1 °C), stability with retention of 90% and 100% coulombic efficiency, after 1150 charge/discharge cycles. In these yields, the incorporation of polyphenylene was relevant, allowing high electrical and ionic conductivities, with a lower RCT compared to LCNM-3, facilitating rapid Li^+^ intercalation/de-intercalation processes and preventing the collapse of LCNM-3, enabling good structural stability cathode [453]. 

In the field of anodes for LIBs, the use of a PBIM-based CMP synthesized by oxidative chemical polymerization in situ has been demonstrated, according to the work of Wei et al. [454]. In addition to the nanofilm structure, the polymeric structure demonstrated abundant microporosity inherent in CMPs, with sizes centered at 0.57 and 1.27 nm. This made it possible to obtain a material with a wide surface area of 711 m^2^ g^−1^, with accessible active sites for Li^+^ ions. Cargo storage would have been based on two factors. On the one hand, the conjugated chains of PBIM and the Li^+^ ions interacted by means of non-covalent forces p-cation, verified by means of the radial distribution function obtained by MD and, on the other hand, the O and N heteroatoms of the respective carbonyl groups and indole, acted as active redox sites [454]. Through DFT calculations, it was determined that the free energy of the reaction for the insertion of Li^+^ ions in PBIM units was lower than for unsubstituted aromatics. The electrodes demonstrated an initial discharge capacity of 1567 mAh g^−1^ and a reversible capacity of up to 1172 mAh g^−1^ at 50 mA g^−1^. Furthermore, the capacity was 510 mAh g^−1^ after 1000 charge/discharge cycles, with an increase in the level of capacity during the first 100 cycles and stabilization until the end of the trial [454]. This type of indole-based material has not been widely studied in battery electrodes and is still an area that may have further development.

Another CP of novel use in LIBs anodes is a poly(isoindigo) (P(iso)) derivative with *n*-doping, synthesized by Mery et al. [455] by a cross-coupling reaction. This polymer was used as a conductive binder/adhesive on Si@C core-shell nanoparticles, forming ~5 nm thick nanolayers. The C and P(iso) layers would have experienced p-p-type interactions, enabling good electrical contact between the materials, as well as good mechanical stability. In this way, unlike the more traditional binders, such as PVDF, CMC, or PAA, that of P(iso) would have allowed a better electrical conductivity, something that has already been proven in previous reports [508,509,510]. When evaluating the Si@CP(iso) anodes, a decrease and subsequent increase in capacity were noticeable during the first 100 operating cycles, which could be explained by the internal restructuring that the polymeric layers would undergo during the Li^+^ ion insertion/disinsertion processes. Then, a stable level in reversible specific capacity was achieved, even with a value of ~1400 mAh g^−1^, after 500 cycles at 0.5 °C. Compared with anodes with CM/CB binders, the compounds by P(iso) demonstrated greater stability at 250 cycles [455]. An investigation such as this opens the possibility of testing other CPs with type n doping, used more frequently in the design of OFETs or OPVs. 

In their use as cathodes of Li-S type batteries, the remarkable surface area and pore volume offered by the COFs of conjugated polymeric structures have shown good performances in the storage of S, as shown by Meng et al. [458]. Through a solvothermal method at 150 °C, the condensation of PyTTA and TA was carried out to form the 3D nanostructure of COF-Py with micropores of ~2.1 nm in diameter. The cathodes designed from COF-Py were loaded with up to 70% by mass of S, being able to develop reversible specific capacities of up to 963.4 mAh g^−1^ after 100 cycles at 1.0 °C and 877.2 mAh g^−1^ after 200 cycles at 2.0 °C. When evaluating in the longer term, a capacity of 481.2 mAh g^−1^ was obtained, corresponding to 73.8% of the initial value, after 550 cycles at 5.0 °C. These COF-Py/S cathodes were compared with one of commercial microporous carbon, which exhibited a reversible capacity of only 265 mAh g^−1^ after 200 cycles at 5.0 °C [458]. The good charge storage reflected in the reversible capacity levels, even at high current speeds, is mainly attributable to the COF-Py conjugated polymer chains, facilitating electronic transport and the ordered micropores, which offered little tortuous trajectories for fast diffusion of Li^+^ ions. 

In the same area is the work carried out by Jiang et al. [469], obtaining similar specific capacity levels from cathodes composed of mesoporous COFs with highly reactive vinyl groups, sulfurized at 200 °C with a reverse vulcanization process. The synthesis of this material, symbolized as S-COF-V was carried out from the condensation of TAPB and DVA, while the vulcanization occurred by the reaction between the vinyl groups and elemental S, forming C-S bonds. The nanofiber morphology of S-COF-V (~100 nm diameter) with abundant nanochannels would have facilitated both ionic diffusion and the incorporation of up to 67% by mass of S [469]. An initial reversible specific capacity of up to 1324 mAh g^−1^ and capacities of 431 and 416 mAh g^−1^ were achieved at a current density of 6 °C and for 1000 cycles of operation at 1 °C, respectively [469].

A high specific capacity in emerging systems such as K-S batteries has been achieved using sulfurized PAN nanoparticles (SPAN) as active materials of the cathodes, synthesized by a solid-state method, as reported by Hwang et al. [469]. Although the cathodes of KS systems would suffer greater volumetric changes during the charge/discharge processes, the SPAN nanoparticle system, in combination with PAA as a binder, allowed better performances in speed capacity and cycle stability compared to the use of PVDF adhesive. During the charge/discharge cycles, the formation/breaking of C-S and S-S bonds in the conjugate structure of SPAN was proposed. The definition of the operating voltage range for the cathodes was significant, obtaining higher specific capacities in the short term, although worse cycle stabilities, in the interval 0.1–3.0 V (initial 1050 mAh g^−1^ at 0.5 °C), with respect to that of 1.0–3.0 V (vs. K^+^/K). In this last potential window, after 500 cycles, a reversible specific capacity of 230 mAh g^−1^ (at 0.5 °C) was obtained, a level that, for the case of 0.1–3.0 V (vs. K^+^/K), is obtained before 300 cycles [469]. Notwithstanding the above, there was no evaluation comparing SPAN with SPAN nanoparticles, but a worse availability of active sites and specific capacity could be expected for the first case.

One of the few reports on the use of COFs within electrodes for SIBs is that of Patra et al. [461]. By a chemical method at 120 °C, they synthesized a triazine-based COF, composed of TFPB and TAPT units, linked by C=N bonds. This polymeric structure of interconnected conjugated chains formed stacked frames and exhibited micropores of ~2.3 nm in diameter, highly ordered, which would have made possible the easy insertion/disinsertion of Na^+^ cations. Theoretically, it was estimated that the maximum capacity of the TFPB-TAPT COF was 233 mAh g^−1^, corresponding to the insertion of a Na^+^ cation for each C=N fraction within the polymer chains [461]. This COF was evaluated as an anode for SIB-type devices, delivering an average reversible capacity of up to 245 mAh g^−1^ at 30 mA g^−1^ and a capacity of up to 125 mAh g^−1^ after 500 cycles, being one of the first reports of this type regarding TFPB-TAPT [461]. Equally novel as an active material for SIB anodes is the use of cyclized PAN nanofibers (cPAN), forming a type of poly(N-heteroacene). According to a study by Gu et al. [462], these nanostructures were synthesized through a method based on electrospinning and thermal stabilization steps. By means of DFT calculations in the study of the structure of polyacene (PAc) and poly(N-heteroacene) bands, lower energies of LUMO (−4.65 eV, vs. −3.02 eV of PAc) and of band gap (0.79 eV) were estimated, vs. 1.06 eV of PAc) for the latter [473]. Therefore, in addition to p-type conjugation, the incorporation of N atoms in the cPAN polymer chains would allow better affinity and electronic transport, maintaining good levels of N-type doping to store Na^+^ cations. When comparing anodes based on PAc and cPAN, reversible capacities of 200 and 527 mAh g^−1^ were seen after 20 cycles of operation at 50 mA g^−1^. Even at a high current density of 5 A g^−1^ and for 3500 cycles, the cPAN anodes retained 99.4% of the initial capacity [462]. On the other hand, cPAN anodes in bulk showed a capacity of 322.6 mAh g^−1^ at 50 mA g^−1^. In this way, not only the importance of N atoms in polymer chains was demonstrated, but also the benefits granted by nanostructuring, increasing the surface area with active sites to store Na^+^ cations.

A rather particular study by Wu et al. [463] is part of the design of cathodes to be used in rechargeable batteries of the Na-Se type. Through the oxidative chemical polymerization of selenophen and subsequent carbonization of PSe, a carbonaceous structure with nanofilm morphology was obtained, in which more Se was incorporated, interacting through physisorption and forming covalent bonds of the Se-C type. With this combination of Se trapped by physical and chemical mechanisms, a reversible and stable capacity of up to 590 mAh g^−1^ (at 100 mA g^−1^) was achieved for more than 200 cycles of operation [463]. For this, an activation stage was essential, where it was operated for some cycles in a voltage window of 0.01–3.0 V (vs. Na^+^/Na) since at a potential close to 0.01 V, the C-Se covalent bonds are broken. This allowed the stored Se, both chemically and physically, to form sodium polyslenides (Na_2_Se_x_) during charge/discharge cycles. The breaking of such bonds would have formed defective areas in the carbonaceous structure, which served to capture the Na_2_Se_x_, preventing their dissolution and loss [463]. Evaluating the long-term cathodes, in potential windows 0.5–3.0 V (vs. Na^+^/Na) (after their activation), during 1000 cycles at 1000 mA g^−1^, a reversible specific capacity of 382 mAh g^−1^ was obtained, corresponding to 91% retention, demonstrating very good stability.

Conjugated microporous polymers (CMPs), due to their widespread p conjugation, physicochemical stability, abundant porosity, and high surface area have emerged as promising materials in energy storage applications, although they have not yet been widely explored. In order to obtain good performance in supercapacitors or battery electrodes, it is important to study and optimize the modifications that allow a better performance of such polymeric structures. Zhang et al. [464] presented a systematic approach to modify the electronic structure of some CMPs, demonstrating their influence on the electrochemical performance by being used as anodes in K^+^ ion batteries (KIBs). Through a chemical route, with a Pd catalyst, derivatives of brominated benzene (Bz) and benzothiadiazole (Bt) were synthesized, in combination with blocks of biphenyl (Ph), spirobifluorene (SF) and pyrene (Py). One of the main results was that the electrochemical yields of the CMPs were closely dependent on the distribution of the lowest energy unoccupied molecular orbital (LUMO), which could be controlled synthetically [464]. The delocalization of the LUMO distribution would benefit a high degree of electronic delocalization along the polymer chains, which in turn increases the redox activity during the charge/discharge processes. Additionally, it was determined that the lower the LUMO energy level and the narrower the band gap, the greater the conductivity and electronic affinity of the CMPs [464]. In accordance with the above considerations, for the polymer based on pyrene-*co*-benzothiadiazole (PyBT) participating as a K^+^ ion storage anode, a reversible capacity of up to 428 mAh g^−1^ (at 30 mA g^−1^) and a up to 272 mAh g^−1^ retention after 500 charge/discharge cycles (at 50 mA g^−1^). With respect to other combinations of the monomers used, the nanoparticles of PyBT with micropore structure, which allowed a good insertion/disinsertion of ions, exhibited the best electrical conductivity with the narrowest band gap [464]. 

## 9. Nanostructured CP Used in Fuel Cells

With a view to being used in cathodes of fuel cells or in Zn-air batteries, electrocatalysts with ORR activity have been developed, from CP, mainly carbonized. In this way, carbonaceous nanostructures of the NC or SC type are achieved, where codoping with O, S, Fe, Mn, Co, Ni, or Pt atoms is also common, or the combination with metal oxides or CNTs. Thus, electrocatalysts of the Fe-N-C, Mn-N-C, Co-N-C, or S-N-C type appear, which are increasingly common to replace others based purely on metals such as Pd, Pt, Ni, and their alloys. N, S, or various metals atoms can modify the electronic distribution in carbonaceous materials, forming active centers capable of breaking O-O type bonds [511,512,513,514]. Among the parameters that have tried to be optimized are the obtaining of more positive start and half-wave potentials, higher oxidation peak current densities, lower Tafel slopes, reaction mechanisms that involve the transfer of four electrons per O_2_ molecule, and high retention of current as a function of time. The standard against which these types of catalysts are compared corresponds to those based on commercial Pt/C.

Another class of engineered electrocatalysts possesses electrocatalytic activity for the oxidation of H_2_O_2_, methanol, ethanol, or 1-propanol. Consequently, they may have potential use in peroxide, DEFC, or DMFC-type fuel cells. These types of devices remain highly dependent on electrodes with metal centers [515,516,517]. For this, the approach of subjecting polymeric nanostructures to pyrolysis, or participating as nanonets/nanolayers, to increase electrical conductivity, long-term stability, and performance in catalytic activity, in metallic or inorganic electrocatalysts is equally feasible. In these cases, they do not provide electrocatalytic active sites but indirectly enhance this activity by improving conductivity. Generally, the parameters to be optimized are similar to those related to ORR, but an attempt is made to achieve less positive start and half-wave potentials. The publications about this type of electrocatalyst and those with ORR activity are less numerous than for supercapacitors and rechargeable batteries. However, there are interesting cases which have been selected to analyze and discuss below.

### 9.1. PANI

The use of PANI in electrocatalysts with ORR-type activity is mainly because it can undergo carbonization, obtaining nitrogen-doped carbons (N-C or NC). In these carbonaceous materials, the catalytic activity of pyridine N and the formation of metal-nitrogen (M-N) centers are important. In general, different studies indicate that it is not enough only with NC-type materials derived from PANI, even though they are nanostructured, but that the combination with carbonaceous materials, transition metals, metal oxides, or metal-organic frameworks is also beneficial.

For factors such as electrical conductivity, structural stability, porosity, and surface area, Liu et al. [518] prepared porous carbon nanosheets co-doped with N and Co, derived from carbonized PANI and interspersed with CNTs. The aniline monomers were polymerized in the presence of acidified CNT, the surfactant CTAB and the CoCl_2_ salt, forming long chains of PANI connected to CNTs. After pyrolysis, CNTs-Co/NC was obtained, showing morphology of highly interconnected porous networks, facilitating ionic diffusion, and having numerous active sites, with CNTs avoiding the self-aggregation of the nano-sheets. The optimal nanostructure was achieved for 200 mg of CNTs (200-CNTs-Co/NC), with a surface area of 1072 m^2^ g^−1^ and pores of average diameter ~10 nm [518]. The 200-CNTs-Co/NC electrocatalyst demonstrated its ORR activity in 0.1 M KOH electrolyte, with starting and half-wave potentials of 0.96 and 0.84 V (vs. RHE), respectively. Additionally, a Tafel slope of ~69 mV decade^−1^ was calculated, being lower than that of commercial Pt/C (71 mV dec^−1^). In the long term, after 10,500 s of operation, the nanostructure only lost 1.0% of current, while for Pt/C, it was 5.0%. When evaluated as a cathode in a Zn-air battery, it developed a maximum power density of ~83 mW cm^−2^, operating for 15 h [518]. Notwithstanding the good electrocatalytic activity of Co, N-C, the incorporation of CNTs made a great difference in being at a level equal to or greater than commercial Pt/C. Recent research, such as that of Shakeel et al. [518], showed a hybrid nanocomposite synthesized by in situ polymerization of PANI, MWCNTs, and platinum NPs decorated on magnetite (Fe_3_O_4_) nanosphere with cherry-like morphology (Pt@Fe_3_O_4_/MWCNT/PANI). This study based on an enzymatic glucose biofuel cell demonstrated a maximum current density of 7.3 mA cm^−2^ with a high open circuit voltage of 0.6 V, concluding the advantages of the synergistic effect of cherry-like morphology because improving the immobilization of GOx by enhancing the electrode’s active surface area.

Under the strategy of mixing PANI and a metal oxide, Lin and Qiao et al. [519] prepared an electrocatalyst based on coral-shaped composites of porous carbon nanotubes doped with N and decorated with Co_3_O_4_ nanoparticles. These hybrids (Co_3_O_4_/N-CP-X) were prepared with in situ polymerization stages with PANI self-assembly, Co_3_O_4_ hydrothermal deposition, and pyrolysis at temperature X (X = 800, 900, or 1000 °C). The best yields were obtained for pyrolysis at 900 °C (Co_3_O_4_/N-CP-900), where the electrocatalyst worked stably in an alkaline medium, with higher electrochemical responses in CV curves. In addition, it developed start and half-wave potentials of 0.97 and 0.90 V (vs. RHE), respectively, being at the commercial Pt/C level (0.99 and 0.89 V). Regarding the current limit density, for Co_3_O_4_/N-CP-900, it was 5.50 mA cm^−2^, while for Pt/C, it was 5.15 mA cm^−2^. These yields were also higher than other hybrids prepared at a lower temperature, which was correlated with the fact that Co_3_O_4_/N-CP-900 showed the highest surface area, reaching 738.3 m^2^ g^−1^. In addition, it showed the best balance in the quaternary and pyridine graphitic N contents, the latter being relevant in the electro-donor behavior, weakening the O-O bond [519]. 

As an alternative to electrocatalysts with Fe-N or Co-N centers, the incorporation of Mn-N appears in carbonaceous structures derived from PANI, as shown by computational and experimental studies by Liu et al. [520]. In DFT calculations, considering a monolayer of water molecules as a solvent, it was determined that the active sites of MnN_4_ could catalyze the ORR by a 4-electron transfer mechanism for a limit potential of 0.53 V. Among the elemental reactions, the highest activation energy was for OOH dissociation in MnN_4_ (0.38 eV), being below those for FeN_4_ and Pt(111) centers (0.42 and 0.43 eV, respectively), predicting the feasibility of the activity of ORR type. On the other hand, using reaction energies calculated by DFT by means of micro-kinetic modeling, a half-wave potential of only 60 and 80 mV was predicted, below Pt(111) and FeN_4_, respectively [520]. To validate experimentally, they prepared PANI hydrogels in the presence of MnCl_2_ and, with subsequent pyrolysis in two stages (at 950 and 900 °C), they obtained Mn-N-C electrocatalysts. When evaluating the ORR activity in an acid medium (H_2_SO_4_ 0.5 M), the Mn-N-C type electrocatalyst showed a half-wave potential of 0.78 V (vs. RHE), only slightly below Fe-N-C (0.80 V). However, in terms of cyclic stability, for Mn-N-C, there was only a 20 mV drop at 10,000 cycles, while Fe-N-C had a loss of 80 mV at 5000 cycles [520]. The important factors for the performance in the ORR activity were the content of Mn and the temperature of the first pyrolysis, 950 °C the optimum.

The integration of bimetallic centers in PANI-derived NC-type structures has also been tested in electrocatalysts for ORR. Deng et al. [521] showed the obtaining of nanocomposites from PANI, CNTs, dicyanamide (DCN), and metal centers of Ni and Co (Ni_1_Co_3_/CN-3) for the ORR in the neutral electrolyte KNO_3_. For the synthesis, a method was used that included in situ chemical polymerization, ball-milling, and pyrolysis, depositing the electrocatalyst on a carbon paper substrate. Regarding Pt/C and other electrocatalysts composed of the partiality of the substances, the Ni_1_Co_3_/CN-3 nanocomposite showed the highest activity towards ORR, with a more positive LSV curve, reaching the highest diffusion-limited current density (4.84 mA cm^−2^) and the lowest Tafel slope (98 mV dec^−1^). When designing a Zn-air battery with a Ni_1_Co_3_/CN-3 cathode and a metallic Zn anode, a maximum power of 38.5 mW cm^−2^ was reached (for 110.2 mA cm^−2^). In addition, three cells were connected in series, powering a 3V LED light for up to 30 days. The best performance of Ni_1_Co_3_/CN-3 was directly related to its morphology and composition. The electrocatalyst consisted of a series of nanoparticles immobilized on the surfaces of the CNTs, with a high surface area, good electrical conductivity, and with active centers for ORR, based on N-pyridine, N-graphitic, Ni-N, and Co-N, contributed by the precursors PANI, DCN and the salts of Ni and Co [521]. This electrocatalyst showed good operation under a neutral electrolyte, avoiding basic KOH or NaOH electrolytes in an aqueous solution, which can be harmful in Zn-air batteries by causing unwanted reactions, degrading the Zn anodes and the cathodes of carbonaceous materials. 

The synergistic combination of materials, including N-C derived from PANI, can result in multifunctional electrocatalytic activity, as shown by Khalid et al. [522]. Through the pyrolysis of PANI complexed with a metal-organic framework based on cobalt (Co) (ZIF-67), a material with good electrocatalytic activity was obtained for the oxidation and oxygen evolution reactions (ORR and OER), and the reaction hydrogen evolution (HER). For the potential use of PANI/ZIF-67 in fuel cells and for Zn-air rechargeable batteries, OER and ORR-type activities are relevant. The electrocatalyst was constituted by a nanolayer of N-C surrounding cobalt nanoparticles (CoNPs) and Co_3_O_4_, in a range of 10–50 nm in diameter. Nanocomposites were prepared with different percentages by mass of PANI, 15% being the optimum, obtaining the highest amount of pyridine N. Due to this and the combination with Co and Co_3_O_4_, when evaluating the ORR-type activity in an alkaline medium, a half-wave potential of 0.75 V (vs. RHE) was reached, close to 0.78 V of a commercial Pt/C catalyst. Furthermore, it showed better stability under a fixed potential of −0.5 V for 5000 s and was not affected by the passage of methanol, compared to Pt/C [522]. Regarding the OER-type activity, results analogous to ORR were obtained, reaching a peak current density of 10 mA cm^−2^ at a potential of 1.56 V, close to 1.58 V of a RuO_2_/C catalyst. When evaluating the performance of the 15% -PANI/ZIF-67 catalyst as a cathode in a Zn-air type battery, a peak current density of ~70 mA cm^−2^ and a peak power density of ~45 mW cm^−2^, comparable to the Pt/C level (~70 mA cm^−2^ and 47 mW cm^−2^, respectively). Although it was not tested in a fuel cell, the above performances allow us to infer its potential use for such devices. 

Regarding the synthesis of electrocatalysts for OER for use in zinc-air batteries, Zhao et al. [523] have used PANI as an auxiliary component to improve the electrocatalytic performances of a nanocomposite that, in addition, includes an oxide of FeCo and MWCNTs (PANI-FeCo/MWCNT). Using an in situ chemical method, they synthesized PANI-FeCo nanoflowers deposited on the networks of MWCNTs, forming a 3D nanohybrid with multiple channels and active sites to enhance OER activity. These nanocomposites operated under alkaline conditions, with 0.1 M KOH electrolyte, presenting a low overpotential (440 mV vs. RHE) at a current density of 10 mA cm^−2^ and a Tafel slope of 55 mV dec^−1^, with better yields. When compared to nanocomposites prepared with monometallic oxides [523], the inclusion of PANI not only allowed a good anchoring of FeCo oxide to MWCNTs, but it would also have contributed to the permeability of the electrolyte and managed to reduce the values of overpotential and Tafel slope from 551 mV (vs. RHE) and 93 mV dec^−1^, respectively. When PANI-FeCo/MWCNT was evaluated as an electrode in a Zn-air battery, it developed a maximum power of 287.4 mW cm^−2^ at 591.3 mA cm^−2^, with a life cycle of over 100 h, these parameters being higher than those of conventional Pt/IrO_2_ electrocatalysts (100.7 mW cm^−2^, ~55 h) [523]. 

### 9.2. PPy

Among the wide variety of materials for obtaining N-doped nanostructured porous carbons, there are significant reports regarding the use of PPy as a precursor subjected to pyrolysis, with electrocatalytic activity for the ORR-type activity. As with PANI, it is usual to combine this nanostructured pyrolyzed polymer with carbonaceous materials or metal oxides. Despite the importance of the nitrogen centers, the incorporation of transition metals is equally relevant, which notably improves the catalytic activity of ORR through the metal-nitrogen-carbon type centers (M-N-C). An example within this scope corresponds to obtaining a catalyst composed of carbon nanofibers doped with Fe and N through a pyrolysis strategy assisted with a protective layer of SiO_2_, as reported by Hu et al. [524]. In the nanocomposite thus obtained (*p*-Fe-N-CNFs), with respect to unprotected C@PPy nanofibers (up-Fe-N-CNFs), the use of SiO_2_ restricted the loss of Fe, it served as a gas trap volatile during pyrolysis and allowed to obtain a higher porosity structure with more N, reaching 6.23% in atomic percentage (1.49% without the use of SiO_2_). In addition, the formation of active centers of the Fex-N type was optimized, avoiding the aggregation of Fe in nanoparticles and obtaining a higher content of pyridine N [524]. These improvements had an impact on the catalytic performance of ORR in an acid medium, being between up-Fe-N-CNFs and Pt C. A peak potential of 0.64 V (vs. RHE) was reached, higher than up-Fe-N-CNFs, but lower than a Pt/C type catalyst. It showed better kinetics with respect to up-Fe-N-CNFs (72.1 mV dec^−1^), reflected in the low overpotential Tafel slope of 64.6 mV dec^−1^. Regarding stability, for a fixed potential of 0.45 V after 10,000 s, the activity of up-Fe-N-CNFs decreased by 4%, whereas Pt/C suffered a loss of ~60 [524]. Thus, although PPy proves to be a good precursor in obtaining N-doped carbon, other modifications allow for increasing the ORR-type catalytic activity, achieving an electrocatalyst suitable for testing in PEMFC.

Electrocatalysts of the Fe-N-C type based on pyrolyzed PPy can be synthesized with Fe_3_O_4_ templates that, in turn, provide the Fe metal centers, as Huang et al. has shown. [525]. They used porous Fe_3_O_4_ microspheres, on whose internal and external surfaces the pyrrole monomers were dispersed. By adding HCl, Fe^3+^ cations were released from Fe_3_O_4_, which activated the in situ polymerization of pyrrole, dissolving the microspheres in turn. With subsequent pyrolysis, hollow double-layer carbon microspheres were obtained and co-doped with Fe and N (Fe-N-DSC). When studying the ORR activity in an alkaline medium, the electrocatalyst showed start and half-wave potentials of 0.061 and -0.131 V (vs. Ag|AgCl), being higher than commercial Pt/C (0.017 and −0.144 V, respectively). In an acid medium, the initial and half-wave potentials of Fe-N-DSC were 0.608 and 0.456 V (vs. Ag|AgCl), also on Pt/C (0.601 and 0.368 V). When evaluating the stability of the current density at 30,000 s of Fe-N-DSC, a retention of 94.3% was obtained, being above the level of Pt/C (70.7%) [525]. The structure of carbonaceous double-layer hollow microspheres doped with Fe and N, exhibiting a high surface area with available active sites, allowed ionic diffusion with smooth trajectories, benefiting ORR activity. Although not tested in fuel cells or Zn-air batteries, they are promising candidates for use as cathodes in these devices. Recent research, such as that of Mashkani et al.[526], showed the synthesis of Fe-ZIF-PPy composite. Moreover, in an anion-exchange direct membrane ethanol fuel cell, two cathodes are made with the optimized electrocatalyst: MEA-1 and commercial 10 wt% Pt/C as MEA-2. The power density of 20 and 9 mW cm^−2^ were obtained at 0.6 V in an air-breathing, respectively, demonstrating that obtaining this nanocomposite with PPY improves properties such as specific surface area, porosity, conductivity, and high density of active sites.

In a different strategy to improve the ORR-type catalyst activity, there is the incorporation of species such as Boron (B) together with transition metals such as Fe. Thus, Yuan et al. [527] showed the manufacture of a porous carbon decorated with centers of B and Fe-Nx species, atomically dispersed, and used as an electrocatalyst for ORR in an alkaline medium. In order to obtain the catalyst, a PPy hydrogel was synthesized by oxidative chemical polymerization, without the use of templates, in the presence of boric acid and FeCl_3_, which was subsequently subjected to pyrolysis. The FeBNC nanocomposite presented a coral-like shape, with nanofibers of 100–200 nm in diameter, highly interconnected, and with graphitic domains. The role of boric acid was important in terms of the optimization of the surface area achieved and the confinement of Fe in coordination centers with N, avoiding its aggregation in nanoparticles, allowing a high number of active sites and easy access to these [527]. Depending on the temperature, for the samples with the presence of B (FeBNC), BET surface areas were obtained in the range of 495–796 m^2^ g^−1^, while for those with FeNC, they were obtained in a range of 338–489 m^2^ g^−1^. The Fe-N-C centers were obtained with an atomic percentage of Fe of 1% and of N in a range of 3.69–7.78%. When evaluating the ORR-type catalyst activity, it was determined that the highest peak potential, in cathodic scanning, was obtained for FeBNC synthesized at 800 °C, reaching 0.872 V (vs. RHE), compared with 0.813 V for FeNC. Regarding the polarization curves, the start and half-wave potentials for FeBNC were 0.968 and 0.838 V, respectively, surpassing the other samples and being close to the commercial Pt/C level (1.012 and 0.851 V, respectively). In addition, the material exhibited greater durability and better tolerance to the passage (crossover) of methanol. In achieving better performance, aspects such as the presence of B and N atoms can be highlighted, which cause an uneven charge distribution in the carbon matrix, enhancing O_2_ adsorption and improving charge transfer; the porous structure with high surface area and more accessible active sites; and the homogeneous presence of Fe_x_-N active sites, with good ORR catalytic activity. 

Without the presence of metallic centers, Shehnaz et al. [528] synthesized PPy nanochains by in situ chemical polymerization under the presence of CTAB and SDS surfactants and with final stages of activation by KOH and pyrolysis at 500–800 °C. The nanochains denoted as PPy-NCs-KOH-T (with T = 500, 600, 700, or 800 °C) were evaluated as supercapacitor electrodes, although in this part, only their electrocatalytic activity for ORR is of interest. For the formation of nanochains, the high concentration of surfactants was essential because, at lower concentrations, only disaggregated spheres were obtained [528]. In addition, the activation by KOH allowed increasing in the porosity and the surface area of the electrocatalyst, which ultimately optimized the number of active sites and ionic diffusion. The best performance nanocomposite was achieved at 800 °C (PPy-NCs-KOH-800), with the most positive starting potential, with respect to the rest of the electrocatalysts prepared at lower temperatures. By means of the Koutecky-Levich graph, a transfer path of ~4 electrons was determined [528].

### 9.3. PEDOT

From the annealing or carbonization of PEDOT, it is possible to obtain carbons doped with S, in which some metal center can be included to enhance the ORR activity. Under this approach, Bhange et al. [529]—as an alternative to Fe-N-C type electrocatalysts—synthesized one of the Fe-S-C types by means of chemical polymerization steps with FeCl_3_ and annealing at temperatures of 800–1000 °C. In this way, highly graphitized carbon nanofilms were obtained, with electrocatalytic centers of the Fe-S_x_ type, uniformly distributed. The best yields were obtained for those prepared at 900 °C (P12–900), with a surface area of 1684 m^2^ g^−1^ and with pore sizes in the range 1–4 nm. Under acidic conditions, with a 0.5 M H_2_SO_4_ electrolyte, its ORR activity was evaluated, obtaining a starting potential of 0.92 V (vs. RHE) and a Tafel slope of 61 mV dec^−1^ (1.0 V and 68 mV decade^−1^ for Pt/C). Due to this, it was tested as a cathode in a PEMFC-type cell, developing a maximum power of 345 mW cm^−2^ (at 0.32 V), being higher than a Fe-NC cathode prepared at 900 °C (306 mW cm^−2^). On the other hand, in the ORR activity in an alkaline medium of 0.1 M KOH, the starting potential was 1.02 V (vs. RHE), even closer to commercial Pt/C (1.04 V). Moreover, in an alkaline medium, it was tested as a cathode in a Zn-air battery, delivering a maximum power of 320 mW cm^−2^, which is at the level of that evaluated for Pt/C [529].

Another way of using PEDOT for cathodes with ORR activity, without resorting to annealing or charring, has been demonstrated by Kaviani et al. [530], who used an oCVD method with liquid oxidants of SbCl_5_ and VOCl_3_, to deposit ~40 nm thick nanolayers of PEDOT on carbon cloth fibers. The most important aspect of this study was the relationship observed between higher electrical conductivity and ORR activity, in turn, dependent on factors at the synthesis and morphological level. Using SbCl_5_ and at 145 °C, the best electrical conductivity was achieved (2100 S cm^−1^), and, when doping with HCl, it even increased to 2590 S cm^−1^, achieving a starting potential of 0.76 V (vs. RHE) for ORR activity in alkaline medium. On the other hand, for PEDOT nanolayers deposited at 145 °C with VOCl_3_ oxidant, a starting potential of 0.73 V (vs. RHE) was obtained. Thus, the ORR activity of the PEDOT nanolayers was shown to be dependent on the electrical conductivity, which, in turn, depended on the use of dopant, type of oxidant, the thickness of the deposit (inverse relationship), and temperature of the oCVD process [530].

In the case of the manufacture of electrocatalysts for the oxidation of alcohols, with potential use in fuel cells, the nanostructured PEDOT has proven to be a feasible material capable of enhancing the electro-oxidative activity of metal centers. An example is the work of Wang et al. [531], who synthesized PEDOT nanonets by oxidative chemical polymerization with HAuCl_4_, decorated with 3.23 nm Au nanoparticles, on flexible DC substrates. The Au nanoparticles were responsible for the electro-oxidative activity of ethanol and 2-propanol in an alkaline medium since only PEDOT/CC electrodes did not show such behavior. PEDOT’s nanonets acted as pathways for electronic transport and increased the surface area where the number of Au nanoparticles was homogeneously distributed, providing more active sites for the electro-oxidation of alcohols. In this way, the yields were higher for electrodes that, in addition to Au, also included PEDOT. For the oxidation of ethanol in Au-PEDOT/CC and Au/CC, starting potentials of −0.3 V (vs. SCE) and −0.2 V, respectively, were obtained, while their corresponding peak currents were 3480 and 68.18 µA. Regarding the oxidation of 1-propanol, the peak currents of Au-PEDOT/CC and Au/CC were 819.9 and 141.4 µA, respectively [531]. Therefore, although PEDOT did not provide active sites in its own chains for the electro-oxidation of alcohols, it was an auxiliary component that enhanced the electrocatalytic activity due to its electrical conductivity, nanostructuring, and good contact with the Au nanoparticles.

Zhang et al. [532] reported a core-shell type nanocomposite based on Au nanoparticles (AuNPs) and PEDOT:PSS, with a view to its use in direct ethanol alkaline fuel cells. The nanocomposite was obtained by means of a synthetic approach assisted by direct current microplasma, in an aqueous solution, at room temperature. A HAuCl_4_ precursor and PEDOT:PSS aqueous dispersions were used for the nanoparticles. Depending on the conditions, it was possible to synthesize Au nanoparticles with average diameters from 4.1 to 50 nm and AuNP/PEDOT:PSS nanocomposites, with nanolayers of the polymer encapsulating the Au center. The incorporation of PEDOT:PSS was mainly due to electrical conductivity and mechanical stability purposes, which impacted the performance of the electrocatalyst. Three samples were studied as catalysts for the ethanol oxidation reaction: one based on solid Pd and the other two from AuNP/PEDOT:PSS on Pd, with average diameters of 4.5 and 35 nm for AuNP. It should be noted that Pd is the metal with the best electrocatalytic activity in ethanol oxidation [532]. When evaluating the responses in CV, the highest peak current densities, both in cathodic and anodic scanning, were for the nanocomposite with a smaller diameter of AuNP with PEDOT:PSS, much higher than that obtained for pure Pd in bulk. The better catalytic activity is not only due to the smaller size of AuNPs but also to the better effectiveness in electron transfer and ease of ionic diffusion, which allowed the porous network of PEDOT:PSS nanolayers. 

The electro-oxidation of methanol has also been demonstrated in nanocomposites such as those described above, with potential use in DMFC-type cells. Rajathi et al. [533], using only electrochemical methods, deposited Cu and Pt nanoparticles on PEDOT amorphous networks with high porosity for electrocatalytic activity. The lowest starting potential, 0.40 V (vs. RHE), for 1 M CH_3_OH, was obtained for 5.48·10^−6^ g cm^−2^ of Pt and 5.94·10^−6^ g cm^−2^ of Cu, demonstrating a trend of higher electrocatalytic activity to higher Pt content. Furthermore, an anodic peak current density of 3738.38 µA cm^−2^ was achieved, much higher than that achieved for commercial Pt/C (981 µA cm^−2^) [533]. However, it is still a metal-dependent electrocatalyst such as Pt since PEDOT has not shown itself to have electrocatalytic activity for the oxidation of methanol. What PEDOT’s porous networks would allow is to increase the surface area with metallic, active sites, with good electrical conductivity and the possibility of better distributing the used metals. 

In a more isolated form, there is the report by Miglbauer et al. [534], who used PEDOT:PSS nanofibers as an electrocatalyst for the reduction in H_2_O_2_ to H_2_O, using it as a cathode in a one-compartment peroxide fuel cell, being the first report of this type. PEDOT in the neutral state showed the best reductive activity towards H_2_O_2_ compared to when it was in *p*-doping conditions. Under acidic conditions, the cathodes showed an open circuit potential of 0.56 V, a current density of up to 2.0 mA cm^−2^, and a maximum power of 0.31 mW cm^−2^. Compared to other publications in this field, the PEDOT:PSS nanofiber cathodes made it possible to achieve a higher power density than a Fe-containing macrocyclic compound (10 µW cm^−2^) [535], an Ag/Ag-Pb alloy (0.075 mW cm^−2^) [536], but below Fe_4_[Fe(CN)_6_]_3_ cathodes (1.55 mW cm^−2^) [537] or pyrazine/Fe[Me(CN)_4_] (with Me = Pt or Pd) (4.2 mW cm^−2^) [538]. Therefore, further optimization would still be lacking, whether morphological, improving nanostructuring, adding metal centers in low proportions, or combining with Fe[Me(CN)_4_] type complexes, to increase the performance of cathodes made by PEDOT.

### 9.4. Copolymers and Derivatives of CP

On the other hand, using a copolymer based on PPy-*co*-PTh and subjecting it to pyrolysis, Li et al. [539] manufactured a porous carbon electrocatalyst co-doped with S, N and with isolated Fe atoms (Fe-ISA/SNC). This electrocatalyst presented a porous nanostructure in the shape of fibrous spheres and active centers of FeN_4_S_2_. The control in the amount of S and N was carried out through the modification in the amounts of thiophene and pyrrole monomers at the time of the chemical synthesis of PPy-co-PTh. The ORR-type reactivity was verified to increase with S doping, demonstrating the best performance at a 1:1 ratio of N:S, for example, in terms of half-wave potential. The start and half-wave potentials for the optimized Fe-ISA/SNC electrocatalyst were 68 and 55 mV (vs. RHE), respectively, being above a commercial Pt/C catalyst. Furthermore, almost no change in the long-term polarization curve was obtained after 15,000 cycles. Through DFT calculations and XAFS analysis, it was shown that the relatively low electronegativity of S increased the electron density in the N atoms, positively impacting the rate-limiting stage—release of OH ions—during the ORR process [539]. In another work, Zhang et al. [540] used a mixture of PPy and PTh synthesized with FeCl_3_, subjecting it to pyrolysis at 800 °C, to form porous carbons doped with N, S, and Fe, forming centers of Fe_3_O_4_ and Fe_1−x_S. The synergistic combination of these centers contributed to better ORR performance. A starting potential of 0.983 V (vs. RHE) was achieved, 17 mV higher than Pt/C. The electrocatalytic activity was mainly due to Fe_3_O_4_/N sites, further enhanced by Fe_1−x_S.

An electrocatalyst of the Fe/N/C type with a hierarchically porous structure was obtained from the PoPD precursor, by means of a semi-closed carbonization approach assisted by a eutectic salt of ZnCl_2_/KCl, according to the work of Li et al. [541]. This molten salt acted as a protector against the loss of carbonized structure and N through evaporation in a temperature range of 390–923 °C, and modulated the hierarchically porous structure and the degree of graphitization of Fe/N/C. Its macropores presented average diameters above 200 nm, while the micro- and mesoporosity were abundantly distributed in the 0.3–5 nm range. For the returns in the ORR activity, the following were important: the degree of graphitization, facilitating the transport of electrons; an optimized surface area with micro- and mesopores with active sites for electrocatalysis and the 3D lattice with macroporosity and abundant channels, for a fast and little tortuous ionic diffusion [541]. Consequently, the Fe/N/C electrocatalyst showed good ORR activity and high tolerance to the presence of methanol, both in acidic and alkaline media, with half-wave potentials of 0.803 and 0.918 V (vs. RHE), respectively. When tested as a cathode in a fuel cell with a Pt/C anode in an acid medium, an open circuit potential of 0.965 V (vs. RHE) and a maximum power density of 576 mW cm^−2^ were obtained. Likewise, it was evaluated as a cathode in a Zn-air type battery with a Zn anode, where the Fe/N/C electrocatalyst developed a maximum power density of 206 mW cm^−2^, and the battery reached a capacity of up to 668 mAh gZn^−1^, with higher yields than the use of Pt/C cathodes (150 mW cm^−2^ and 602 mAh gZn^−1^) [541].

Based on the pyrolysis of the same CP, chemically synthesized PoPD, Liu et al. [542] manufactured bifunctional electrocatalysts with HER and ORR activity, characterized by having a porous carbonaceous nanostructure and co-doped with Co and N (Co, N-C). In the template-assisted synthesis of SiO_2_, the precursors of melamine and PoPD contributed to the content of N, while CoCl_2_ added the content of Co. The use of SiO_2_ and carbonization at 900 °C allowed to obtain mesopores with an average diameter of ~22 nm and a surface area of 413.1 m^2^ g^−1^, facilitating ionic diffusion and optimizing the number of active sites. In this case, it is worth highlighting the ORR-type activity in an alkaline medium, where the Co, N-C electrocatalysts synthesized at 900 °C (Co, N-C900) had better performances in relation to those obtained by carbonization at lower temperatures. Not only were the previous morphological optimizations significant, but also the higher content of sites with graphite N and Co-N, fundamental for ORR. The electrocatalysts type Co, N-C900, showed initial and half-wave potentials of 0.97 and 0.85 V (vs. RHE), with a current limit density of 5.82 mA cm^−2^ (at 0.4 V), being parameters at the level of Pt/C (0.97 V, 0.88 V, and 5.68 mA cm^−2^, respectively) [542]. In addition, it showed a lower sensitivity to the passage of methanol in relation to Pt/C. 

Under the same approach of using a SiO_2_ template, but adding a ZnCl_2_ salt, Wu et al. [543] prepared a kind of combined template in the form of nanospheres to synthesize a hierarchically porous 3D Fe/N/C electrocatalyst, using the PoPD polymer as a precursor. During the final pyrolysis process, ZnCl_2_ was the first component to boil, leaving an abundant amount of micro- and mesopores, while SiO_2_ macropores were obtained.

In this way, the Fe/N/C electrocatalyst synthesized with the combined ZnCl_2_-SiO_2_ template presented the highest surface area with active sites (1538.4 m^2^ g^−1^) and the highest half-wave potential (0.785 V vs. RHE), compared to Fe/N/C synthesized with these components separately [543]. In both acid and alkaline media, this Fe/N/C electrocatalyst demonstrated ORR-type activity and when evaluated as a cathode in a PEMFC-type cell, a maximum power density of 480 mW cm^−2^ was reached. Thus, it demonstrated the advantages that can be achieved from porosity on a different scale, improving the yields in the electrocatalytic activity.

A nanostructuring of greater morphological variety in this type of polymeric material can also be achieved with the assistance of two types of removable templates, as shown by Tian et al. [544]. Through a co-assembly strategy, with the assistance of the PS-b-PEO copolymer and the inorganic material 2D LDH, they synthesized PmPD by chemical polymerization. From the morphology of LDH nanoflowers and nanoflowers, through pyrolysis at 800 °C, they obtained N-doped porous carbon nanoflowers and nanoflowers (NMHCSs and NMCFs, respectively). The NMHCS-type electrocatalyst showed a nanostructure with mesopores of ~14 nm in average diameter, a surface area of 256 m^2^ g^−1,^ and an N content of ~4% by mass. When evaluating its ORR-type activity in an alkaline medium, a half-wave potential of 0.77 V (vs. RHE) and a current limit density of 5.0 mA cm^−2^ were obtained. On the other hand, for NMCF, a higher surface area (266 m^2^ g^−1^) and better ORR activity were obtained, showing LSV curves closer to Pt/C, with half-wave potential, limited current density and transferred electrons, of 0.80 V (vs. RHE), 5.5 mA cm^−2^ and 3.9, respectively [544]. These NMCF yields would be explained by the type of nanoflower design, which would prevent the self-aggregation of nanoflowers, causing less blockage of pores and channels, something that would occur in NMHCS. 

As proof of concept, recent reports have appeared in the field of CMPs with ORR-type electrocatalytic activity, as reported by Roy et al. [545], who synthesized two of these polymeric structures, using Sonogashira-Hagihara coupling. The semiconductor CMPs that showed ORR activity were based on TPA-BP-1 and TPA-TPE-2, where the TPA units are characterized by being electro-donors, while BP-1 and TPE-2 are electro-acceptors. In addition to their micropores with available active sites, the dual acceptor-donor characteristic in the CMPs units would have favored electron delocalization, impacting the better catalytic activity for ORR [545]. The starting potential and the current limit density for CMP-TPA-BP1 were 0.80 V (vs. RHE) and ~6 mA cm^−2^, while for CMP-TPA-TPE-2, they were 0.82 V (vs. RHE) and ~8 mA cm^−2^, respectively. This correlated with the bandgap energies calculated for CMP-TPA-BP-1 and CMP-TPA-TPE-2, with values of 2.19 and 1.69 eV, respectively. Additionally, through DFT calculations, binding energies between O_2_ and N sites were obtained, in which for CMP-TPA-TPE-2, its value was higher by 1.02 eV, with respect to CMP-TPA-BP-1 [545]. Although the various parameters were more favorable for CMP-TPA-TPE-2, both CMPs showed good ORR activity to be potentially used as cathodes in fuel cells or in Zn-air batteries.

In the case of ORR electrocatalysts made from COFs, Xu et al. [546], with the assistance of phytic acid as a template, pyrolyzed 2D COFs of TAPT-DHTA at 1000 °C to form N and S-doped graphitized carbon nanofilms. Abundant distribution of micropores in the range 0.7–1.6 nm, with a surface area of 495 m^2^ g^−1^, superior pyrolyzed COFs without phytic acid. This template prevented the COF-TAPT-DHTA nanosheets from collapsing and, by decomposing during pyrolysis, added P atoms to the carbonaceous nanostructure. In this way, nanosheets with abundant edges doped with N and P were obtained, which acted as active sites for ORR. Despite the above, even higher yields, surface area (1160 m^2^ g^−1^), and porosity in a range of 0.5–6 nm were achieved by subjecting to a second pyrolysis at 900 °C in the presence of NH_3_ (PA@TAPT-DHTA-COF_1000NH3_). In an alkaline medium, this electrocatalyst showed more positive start and half-wave potentials than commercial Pt/C, at 30 and 50 mV, respectively, in accordance with its LSV curves: a higher limit current density, being 1.2 mA cm^−2^ higher than Pt/C and a Tafel slope of 110 mV dec^−1^ (121 mV dec^−1^ for Pt/C). When comparing its stability during 5.5 h, it showed a drop of only 5% of the initial current density, whereas, for Pt/C, 20% was obtained [544]. In addition, in relation to other doped carbonaceous electrocatalysts, it has shown an equal or higher level in terms of the parameters already mentioned [547,548,549,550,551].

## 10. Conclusions

In summary, a state-of-the-art has been captured here that demonstrates the advancement of polymeric nanostructures, showing the variety of optimal designs with potential use in energy storage devices, to then discuss and analyze in detail recently tested nanostructures in these. Thus, an abundant variety of nanostructures designed and obtained through different methods has been reported, which have been reported for potential use in energy storage devices, electrocatalysts, sensors, and remediators. It has been shown that there are a wide variety of polymeric nanostructures that have been achieved to optimize the desired performances of the applications in question, both in pure form and by forming composites.

It has been verified that different electrode designs for energy storage devices, which include conventional nanostructured CP or other polymeric nanostructures, account for the achievement of yields at the level of reports with inorganic components, or even sometimes higher. Inorganic materials to make energy storage devices have shown that the formation of hybrids that include polymeric nanostructures and some other carbonaceous component or a secondary additive can improve parameters such as electrical conductivity, capacitance or specific capacity, long-term stability, and capacity of velocity.

In general, it has been shown that in very limited publications, performance parameters are reported with figures of merit, such as standard deviation, reproducibility, or confidence limit, which allow the validation of the reported results. Anyway, it has been possible to deliver a projection of possible polymeric nanostructures with conductive and electrochemical activity, to be used in electrodes of energy storage devices. In the field of flexible electrodes, the formation of hydrogels has shown recent advances that are promising and that allow projecting a broader use of this approach in other types of CP, optimizing the amount and type of dopants/interconnectors. It has also been proposed to explore new designs in traditional CP derivatives, copolymers, quinone-based polymers, CMPs, COFs, and their mixtures with other classes of materials, taking advantage of the possibilities offered by electrochemical, oCVD, or self-assembly methods. Finally, it is projected that the use of computational methods that combine DFT, machine learning, data mining, and artificial intelligence to optimize and economize the design process of energy storage devices based on nanostructured CP would be beneficial.

## Figures and Tables

**Table 1:** Nanostructured PANI, in combination with carbonaceous materials, was evaluated as electrodes for supercapacitors, with their performance parameters.

#	Electrodes (Anode//Cathode)	Electrolyte; Potential Windows	Capacitance or Capacity	Current Density	Power Density	Energy Density	Cycling Rate (%)	Ref
01	Nanofibers-PANI/ rGO	KOH 6 M; −1.0 a 0.0 V (vs. SCE)	445.7 F g^−1^, 0.5 A g^−1^	~68%, 20 A g^−1^	-	-	-	[208]
02	Nanofibers-PANI/ rGO//NiO/rGO	KOH 6 M; 1.6 V	270.1 F g^−1^, 1 A g^−1^	54.2%, 20 A g^−1^	13,794 W kg^−1^	81 Wh kg^−1^	~100%, 100,000 cycles, 5 A g^−1^	[208]
03	FrGO/ Nanofibers-PANI	H_2_SO_4_ 1 M; 0.4 V	692 F g^−1^/629.7 mF cm^−2^, 1 A g^−1^	53.5%, 40 A g^−1^	-	-	83.3%, 1000 cycles, 10 A g^−1^	[209]
04	FrGO/ Nanofibers-PANI // FrGO/ Nanofibers-PANI	Gel H_2_SO_4_/PVA; 0.4 V	324.4 F g^−1^, 1 A g^−1^	-	300 W kg^−1^	16.3 Wh kg^−1^	-	[209]
05	CF@rGO/ Nanofibers-PANI // CF@rGO/ Nanofibers-PANI	H_2_SO_4_ 1 M; 0.8 V	868.5 F g^−1^ a 1 A g^−1^	~95.4%, 5 A g^−1^	-	-	94.1%, 2000 cycles, 10 A g^−1^	[210]
06	SS/rGO/ Nanofibers-PANI	H_2_SO_4_ 1 M; 0 a 0.8 V (vs. SCE)	603.7 F g^−1^ a 1.1 A g^−1^/4969.7 mF cm^−2^ (8.9 mA cm^−2^)	80%, 53.6 mA cm^2^	-	-	-	[211]
07	SS/rGO/ Nanofibers-PANI//SS/rGO/ Nanofibers-PANI	Gel H_2_SO_4_/PVA; 0.8 V	1506.6 mF cm^−2^, 6 mA cm^−2^	54%, 30 mA cm^−2^	-	-	92%, 5000 cycles, 50 mA cm^−2^	[211]
08	Gel rGO@PANI 3D porous	H_2_SO_4_ 1 M; 0.0 a 0.8 V (vs. SCE)	824 F g^−1^, 2.22 A g^−1^/5830 mF cm^−2^ a 15.72 mA cm^−2^	98%, 53.33 A g^−1^	-	-	73%, 5000 cycles	[212]
09	Hydrogels PANI/rGO	H_2_SO_4_ 1 M: 0.8 V	~300 F g^−1^, 0.42 A g^−1^	~53%, 4.20 A g^−1^	-	-	93%, 5000 cycles, 4.2 A g^−1^	[213]
10	Hydrogels PANI/rGO// Hydrogels PANI/rGO	Gel H_2_SO_4_/PVA; 1.0 V	112 F g^−1^, 0.08 A g^−1^	~54%, 1.26 A g^−1^	30.77 mW cm^−3^	8.80 mWh cm^−3^ (max)	86%, 17,000 cycles, 1.26 A g^−1^	[213]
11	Hydrogels NG/PANI	H_2_SO_4_ 2 M; −0.2–0.6 V (vs. Ag/AgCl)	514.3 F g^−1^, 1 A g^−1^	~80%, 20 A g^−1^	-	-	87.1%, 1000 cycles, 10 A g^−1^	[214]
12	Hydrogels NG/PANI//Hydrogels NG/PANI	Gel H_2_SO_4_/PVA; 1.0 V	584.7 mF cm^−2^, 1 mA cm^−2^	62.9%, 20 mA cm^−2^	500–9999 µW cm^−2^	81.28–51.08 µWh cm^−2^	-	[214]
13	Gels GO/PANI//Gels GO/PANI	Gel H_2_SO_4_/PVA; 0.8 V	~44 mF cm^−2^, 0.1 mA cm^−2^	~34%, 2.5 mA cm^−2^	-	-	-	[215]
14	Nano-arrangements of PANI/GO-H_2_SO_4_	H_2_SO_4_ 1 M; −0.2–0.8 V (vs. SCE)	727 F g^−1^, 1 A g^−1^	~60%, 20 A g^−1^	-	-	-	[216]
15	Nano-arrangements of PANI/GO-H_2_SO_4_//Nano-rods of PANI/GO-H_2_SO_4_	H_2_SO_4_ 1 M; 0.8 V	447 F g^−1^, 0.5 A g^−1^	~54%, 20 A g^−1^	0.4–15.3 kW kg^−1^	40–19.8 Wh kg^−1^	95.7%, a 5000 cycles, 100 mV s^−1^	[216]
16	rGO/ nanoparticles- PANI//rGO/ nanoparticles- PANI	Gel H_2_SO_4_/PVA; −0.2–0.8 V (vs. SCE)	850 mF cm^−2^/424.4 F g^−1^, 0.5 A g^−1^	~35%, 4 A g^−1^	-	-	~80%, 2000 cycles, a 7 mA cm^−2^	[217]
17	G/nanorods- PANI/nanofibers- PANI	H_2_SO_4_ 1 M; 0.8 V	578 F g^−1^, 1 A g^−1^	72.7%, 10 A g^−1^	-	-	-	[218]
18	Nano-sheets-G//G/nano-rods- PANI/nanofibers PANI	H_2_SO_4_ 1 M; 1.0 V	135 F g^−1^, 1 A g^−1^	73%, 10 A g^−1^	1–10 kW kg^−1^	18.8–13.6 Wh kg^−1^	93%, 10,000 cycles, 2 A g^−1^	[218]
19	Nanorods- PANI/G	H_3_PO_4_ 1 M; 0.8 V	729 mF cm^−2^, 0.1 mA cm^−2^	~73%, 1 mA cm^−2^	-	-	-	[219]
20	Nanorods- PANI/G//Nanorods- PANI/G	Gel H_3_PO_4_/PVA; 0.8 V	230 mF cm^−2^, 0.1 mA cm^−2^	~71%, 1 mA cm^−2^	-	-	86.9%, 8000 cycles, 0.8 mA cm^−2^	[219]
21	Nanorods- PANI/G//Nanorods- PANI/G	Gel EMITFSI/PVDF-HFP; 3.0 V	119 mF cm^−2^, 0.6 mA cm^−2^	~40%, 10 mA cm^−2^	0.9–15 mW cm^−2^	37.2–14.1 µWh cm^−2^	-	[219]
22	Nanofibers-PVA/G/nanowires-PANI/G//Nanofiber-PVA/G/nanowires-PANI/G	H_2_SO_4_ 1 M; 0.8 V (vs. anode)	90.0 F g^−1^/310 mF cm^−2^, 3 mA	~73.2%, 30 A g^−1^	~0.8–5.8 qaµW cm^−2^	~26–19 µWh cm^−2^	75.6%, 88,000 cycles	[220]
23	G@PANI// G@PANI	EMIMBF_4_	176 F g^−1^, 0.5 A g^−1^	80.7%, 10 A g^−1^	451–10,000 W kg^−1^	38–75 Wh kg^−1^	80.3%, 10,000 cycles, 10 A g^−1^	[221]
24	G@PANI// G@PANI	Polymer gel EMIMBF_4_/PVDF-HEP	180 F g^−1^, 1 A g^−1^	86%, 10 A g^−1^	-	-	85%, 10,000 cycles, 10 A g^−1^	[221]
25	Fibers of cotton/G/ Nanowires-PANI	H_2_SO_4_ 1 M; −0.2–0.8 V (vs. SCE)	246 mF cm^−2^, 5 mV s^−1^	~24%, 100 mV s^−1^	-	-	98%, 3800 cycles, 1 A g^−1^	[222]
26	Fibers of cotton/G/ Nanowires-PANI // Fibers of cotton/G/ Nanowires-PANI	Gel H_3_PO_4_/PVA; −0.2–0.8 V (vs. SCE)	70.1 mF cm^−2^, 5 mV s^−1^ (0.22 A g^−1^)	~36%, 0.30 A g^−1^	840.9 µW cm^−2^	9.74 µWh cm^−2^ (max)	-	[222]
27	3D PC-G/PANI	H_2_SO_4_ 1 M; 0.8 V	1198 F g^−1^, 2 A g^−1^	83.5%, 30 A g^−1^	-	-	-	[223]
28	3D PC-G/PANI//3D PC-G/PANI	H_2_SO_4_ 1 M; 1.0 V	220 F g^−1^, 2 A g^−1^	72%, 20 A g^−1^	15 kW kg^−1^ max.	61 Wh kg^−1^ max.	94%, 10,000 cycles, 5 A g^−1^	[223]
29	Nano-arrangements of PANI/G	H_2_SO_4_ 1 M; −0.2–0.8 V (vs. SCE)	912 F g^−1^, 1 A g^−1^	86.4%, 20 A g^−1^	-	-	89.5%, 10,000 cycles, 10 A g^−1^	[224]
30	Nano-arrangements of PANI/G	Gel H_2_SO_4_/PVA; 0.8 V	120 F g^−1^, 1 A g^−1^	-	850–3200 W kg^−1^	13.5–30 Wh kg^−1^	90%, 5000 cycles, 10 A g^−1^	[224]
31	Fibers-CNTs/3D porous-CNTs@ nano-granos-PANI// Fibers-CNTs/3D porous-CNTs@ nanoparticles-PANI	Gel H_3_PO_4_/PVA; 0.8 V	67.31 mF cm^−2^, 0.5 mA cm^−2^	~89.2%, 4.0 mA cm^−2^	-	-	90%, 5000 cycles, 1 mA cm^−2^	[225]
32	CNF@CNT@PANI	H_2_SO_4_ 1 M; 0.8 V	1119 F g^−1^, 1 A g^−1^	~71%, 5 A g^−1^	-	-	~98%, 2000 cycles, 10 A g^−1^	[226]
33	SWCNTs/PANI	H_2_SO_4_/PVA; 1.0 V	15.8 mF cm^−2^, 0.044 mA cm^−2^	39.3%, 0.44 mA cm^−2^	-	-	~82%, 2400 cycles, 0.13 mA cm^−2^	[227]
34	Nano CNT/G@PANI	Gel H_3_PO_4_/PVA; 0.8 V	182.6 F g^−1^, 0.2 A g^−1^	13%, 8 A g^−1^	-	-	77.3%, 5000 cycles, 1 A g^−1^, 800%	[228]
35	G/nanorods -PANI/CNTs	H_2_SO_4_ 1 M; 0.7 V	638 F g^−1^, 0.5 A g^−1^	88.2%, 10.0 A g^−1^	-	-	93%, 2000 cycles, 0.5 A g^−1^	[229]
36	fibers-CF@CNF@ Nano-hilos-PANI	Gel H_2_SO_4_/PVA; 0,8 V	234 mF cm^−2^, 0.1 mA cm^−2^/159.67 F g^−1^, 5 mV s^−1^	~68.4%, 1 mA cm^−2^	0.2–2 W m^−2^	3.6–5.2 µWh cm^−2^	90%, 8000 cycles	[230]
37	3D Nanorods PANI/C mesoporous	H_2_SO_4_ 1 M; −0.2–0.8 V (vs. SCE)	715 F g^−1^, 1 A g^−1^	60%, 10 A g^−1^	250–5000 W kg^−1^	24.64–13.79 Wh kg^−1^	93.6%, 10,000 cycles, 100 mV s^−1^	[231]

**Table 2:** Nanostructured PANI in various configurations, evaluated for supercapacitor electrodes, with their performance parameters.

#	Electrodes (Anode//Cathode)	Electrolyte; Potential Windows	Capacitance or Capacity	Current Density	Power Density	Energy Density	Cycling Rate (%)	Ref
01	3D coaxial Fe_3_O_4_/PANI	H_2_SO_4_ 1.0 M; 0–0.8 V (vs. SCE)	620 F g^−1^, 1.0 A g^−1^	42.3%, 10 A g^−1^	~400–1300 W kg^−1^	52.4–22.1 Wh kg^−1^	85%, 2000 cycles, 2.0 A g^−1^	[232]
02	Hydrogels 3D α-Fe_2_O_3_/PANI	Na_2_SO_4_ 1.0 M; −1.0–0.0 V (SCE)	473.6 F g^−1^/236.8 mF cm^−2^, 1 A g^−1^	~52%, 250 mV s^−1^	-	-	98.2%, 5000 cycles, 100 mV s^−1^	[233]
03	Hydrogels 3D α-Fe_2_O_3_/PANI//de PANI Gels	LiCl/PVA Gels; 1.8 V	202.7 C g^−1^, 1 A g^−1^	82.6%, 10 A g^−1^	67.1 mW cm^−3^	0.31 mWh cm^−3^ máx,	80.3%, 5000 cycles, 10 A g^−1^	[233]
04	PANI 3D-porous@ Nanosheet- MnO_2_	Na_2_SO_4_ 1.0 M; 0.0–0.8 V (SCE)	467.9 F g^−1^/117.7 F cm^−3^/3883.5 mF cm^−2^, 1 A g^−1^	48.7%, 150 mV s^−1^	-	-	98.5%, 10,000 cycles, 50 mV s^−1^	[234]
05	Nanofibers of MnFe_2_O_4_/PANI	H_2_SO_4_ 1.0 M; −0.2–1.0 V (vs. SCE)	329 F g^−1^, 1 A g^−1^	~83.3%, 10 A g^−1^	-	-	-	[235]
06	Nanofibers of MnFe_2_O_4_/PANI//Nanofibers of MnFe_2_O_4_/PANI	H_2_SO_4_ 1.0 M; 1.2 V	269 F g^−1^, 0.5 A g^−1^	~77%, 10 A g^−1^	298.6–5419.0 W kg^−1^	13.3–8.4 Wh kg^−1^	97%, 10,000 cycles, 5 A g^−1^	[235]
07	Microspheres MoS_2_/PANI	H_2_SO_4_ 1.0 M; −0.2–0.6 V (vs. Ag/AgCl)	360 F g^−1^, 0.8 A g ^−1^	~72%, 20 A g^−1^	32–23.1 W kg^−1^	8320–320 Wh kg^−1^	85.4%, 8000 cycles, 10 A g^−1^	[236]
08	Microspheres MoS_2_/PANI//Microspheres MoS_2_/PANI	H_2_SO_4_ 1.0 M; −0.2–0.6 V (vs. Ag/AgCl)	231 F g^−1^, 0.2 A g^−1^	~10.8%, 2 A g^−1^	-	-	80.4%, 5000 cycles, 1 A g^−1^	[236]
09	2D Nanosheet MXeno Ti_3_C_2_T_x_/ PANI	H_2_SO_4_ 3 M; −0.7–0.2 V (vs. Ag/AgCl)	~412 F g^−1^/1353 F cm^−3^, 1 A g^−1^	~64%, 50 A g^−1^	-	-	98.3%, 10,000 cycles, 20 mV s^−1^	[237]
10	2D Nanosheet MXeno Ti_3_C_2_T_x_/ PANI// 2D Nanosheet MXeno Ti_3_C_2_T_x_/ PANI	H_2_SO_4_ 3 M; −0.7–0.2 V (vs. Ag/AgCl)	130 F g^−1^/~575 F cm^−3^, 2 mV s^−1^	~38.5%, 10 V s^−1^	~575 W L^−1^	79.8 Wh L^−1^	-	[237]
11	Nanocomposite of rGO/Cu_2_O-CuO/ PANI // Nanocomposite of rGO/Cu_2_O-CuO/ PANI	H_2_SO_4_ 0.4 M; 1.2 V	684.93 F g^−1^, 0.25 A g^−1^	~26%, 10 A g^−1^	1315,76 W kg^−1^	136.98 Wh kg^−1^ máx,	84.3%, 5000 cycles, 700 mV s^−1^	[238]
12	Nanofiber-PANI/ MnO_2_-rGO/ nanofibers-PANI // Nano-fibers-PANI/ MnO_2_-rGO/ nanofibers-PANI	Gels KOH/PVA; 1.0 V	148 F g^−1^, 1 A g^−1^	73.6%, 5 A g^−1^	0.521 kW kg^−1^	20.5 Wh kg^−1^ máx,	~78%, 5000 cycles, 5 A g^−1^	[239]
13	Nanofibers-PANI/ MnO_2_-rGO/ nanofibers-PANI//MoO_3_/GF	Gels KOH/PVA; 1.6 V	146 F g^−1^, 1 A g^−1^	~73.3%, 5 A g^−1^	0.838–4.368 kW kg^−1^	51.91–38.15 Wh kg^−1^	82%, 5000 cycles, 5 A g^−1^	[239]
14	Layer by layer of G/Fe_3_O_4_/PANI:PSS	0.1 M HCl; −0.1–0.6 V (vs. Ag/AgCl)	768.6 F g^−1^, 1 A g^−1^	~22.3%, 5 A g^−1^	350–1750 W kg^−1^	52.3–11.7 Wh kg^−1^	84%, 1600 cycles, 3 A g^−1^	[240]
15	Nanocomposite LaMnO_3_/rGO/PANI	KOH 3 M; 0.0–0.5 V (vs. Ag/AgCl)	802 F g^−1^, 1 A g^−1^	55%, 15 A g^−1^	-	-	-	[241]
16	rGO//Nanocomposite LaMnO_3_/rGO/PANI	Gels KOH/PVA; 1.8 V	111 F g^−1^, 2.5 A g^−1^	50%, 20 A g^−1^	2.25–18 kW kg^−1^	50–25 Wh kg^−1^	116%, 100,000 cycles, 20 A g^−1^	[241]
17	3D CC/NiCo_2_O_4_@ NiMoO_4_/ nanorods- PANI // 3D CC/NiCo_2_O_4_@ NiMoO_4_/ nanorods- PANI	H_3_PO_4_/PVA Gels; 0.1–0.8 V (vs. SCE)	1322.2 F g^−1^/2380 mF cm^−2^, 1.0 mA cm^−2^	63.4%, 10.0 mA cm^−2^	443.2 W kg^−1^	90 Wh kg^−1^ max,	92.36%, 5000 cycles, 1.0 mA cm^−2^	[242]
18	Nanowires-Ag@C@nanowires-PANI	H_2_SO_4_ 1 M; −0.3–0.7 V (vs. SCE)	785 C g^−1^, 0.5 A g^−1^	47.1%, 10 A g^−1^	-	-	94.1%, 3000 ciclos, a 1 A g^−1^	[243]
19	Ti@G@ Nanowires-PANI	H_2_SO_4_ 0.5 M; 0.0–0.8 V (vs. Ag/AgCl)	623.1 F g^−1^, 1 A g^−1^	~86%, 40 A g^−1^	-	-	-	[244]
20	Ti@G@ Nanowires-PANI // Ti@G@ Nanowires-PANI	H_2_SO_4_ 0.5 M; 0.8 V	320.8 F g^−1^, 1 A g^−1^	~90%, 20 A g^−1^	383–4170 W kg^−1^	26.14–6.95 Wh kg^−1^	86%, 10,000 cycles, 4 A g^−1^	[244]
21	Nanosheet MoS_2_/PANI/rGO	H_2_SO_4_ 1 M; −0.2–0.7 V (vs. Ag/AgCl)	520.0 F g^−1^, 1 A g^−1^	~44.9%, 30 A g^−1^	-	-	81.9%, 40,000 cycles, 10 A g^−1^	[245]
22	Nanosheet MoS_2_/PANI/rGO // Nanosheet MoS_2_/PANI/rGO	H_2_SO_4_ 1 M; 0.9 V	111.1 F g^−1^, 0.5 A g^−1^	~60%, 4.0 A g^−1^	-	-	84.2%, 20,000 cycles, 2 A g^−1^	[245]
23	CC/MoS_2_@ nanosheet-PANI	H_2_SO_4_ 1 M; −0.2–0.6 V (vs. Ag/AgCl)	972 F g^−1^, 1 A g^−1^	75%, 20 A g^−1^	-	-	87%, 5000 cycles, 10 A g^−1^	[246]
24	CC/MoS_2_@ nanosheet-PANI // CC/MoS_2_@ nanosheet-PANI	H_2_SO_4_/PVA Gels	193.3 F g^−1^, 1 A g^−1^	75.9%, 20 A g^−1^	14–0.42 kW kg^−1^	8.56–17.18Wh kg^−1^	81%, 2000 cycles, 10 A g^−1^	[246]
25	Hydrogels of Nanofibers of cellulose@PANI-PVA	H_2_SO_4_ 1.0 M; 0.0–0.4 V (vs. SCE)	226.1 F g^−1^, 0.2 A g^−1^	~80.7%, 2.0 A g^−1^	-	-	74%, 5000 cycles, 5.0 A g^−1^	[247]
26	Hydrogels 3D PANI/phytic acid	H_2_SO_4_ 1.0 M; 0.0–0.8 V (vs. SCE)	311.3 F g^−1^/561.7 mF cm^−2^, 1 mA cm^−2^	67.5%, 10 mA cm^−2^	-	-	-	[248]
27	Hydrogels 3D PANI/phytic acid// Hidrogels 3D PANI/phytic acid	H_2_SO_4_/PVA Gels; 0.8 V	77.6 F g^−1^, 0.5 A g^−1^/135.9 mF cm^−2^, 0.5 mA cm^−2^	72%, 5 A g^−1^	0.4–4 mW cm^−2^	40.0–33.7 µWh cm^−2^	76%, 10,000 cycles, 2 mA cm^−2^	[248]
28	Microspheres of NC/ Nanowires-PANI	H_2_SO_4_ 1.0 M; 0.0–0.8 V (vs. Ag/AgCl)	500 F g^−1^, 1 A g^−1^	77%, 20 A g^−1^	-	-	91.8%, 5000 cycles, 5 A g^−1^	[249]
29	Microspheres of NC/ Nanowires-PANI // Microspheres of NC/ Nanowires-PANI	H_2_SO_4_ 1.0 M; 1.1 V	100 F g^−1^, 1 A g^−1^	75.6%, 7 A g^−1^	-	-	95.4%, 5000 cycles, 5 A g^−1^	[249]
30	PLA/CNTs/PANI porous	H_2_SO_4_ 1.0 M; −0.2–0.8 V (vs. SCE)	510.3 F g^−1^, 1.0 A g^−1^	80.9%, 10.0 A g^−1^	-	-	111.5%, 2000 cycles, 100 mV s^−1^	[250]
31	Macroporous carbon 3D of kenaf/rGO/PANI	H_2_SO_4_ 1.0 M; −0–2- 0.8 V (vs. SCE)	1224 F g^−1^, 0.3 A g^−1^	~51%, 5.0 A g^−1^	0.218–74.15 kW kg^−1^	144.4–1.86 Wh kg^−1^	87%, 5000 cycles, 1.0 A g^−1^	[251]
32	Red 3D Cellulose bacterial/PANI/ NiCo-LDH	KOH 2 M; 0–0.45 V (vs. SCE)	1690 F g^−1^, 1 A g^−1^	~46%, 15 A g^−1^	-	-	83.2%, 5000 cycles, 10 mV s^−1^	[252]
33	NC(cellulose) bacterial/CC//Red 3D Celullose bacterial/PANI/ NiCo-LDH	KOH/PVA Gels; 1.6 V	133.1 F g^−1^, 1 A g^−1^	~60%, 10 A g^−1^	828.9–8263.3 W kg^−1^	47.3–28.4 Wh kg^−1^	91.4%, 3000 cycles, 5 A g^−1^	[252]
34	Hydrogels of PANI	H_2_SO_4_ 1.0 M	636 F g^−1^, 2.0 A g^−1^/1.54 F cm^−2^, 5.0 mA cm^−2^	98.4%, 25.0 A g^−1^	-	-	83.3%, 10,000 cycles, 35 A g^−1^	[253]
35	PANI//PR-Br	Polymer gels SPAn-PMMA (5%)	1.27 mF cm^−2^/123 F g^−1^	-	58.8 kW kg^−1^	13.5 Wh kg^−1^	-	[254]
36	Nanowires-NC/CP	H_2_SO_4_ 1.0 M; −0.2–0.8 V (vs. SCE)	404.0 F g^−1^, 1.0 A g^−1^	78%, 0–10 A g^−1^	-	-	98.1%, 5000 cycles, 1.0 A g^−1^	[255]
37	Nanowires-BNC/CP	1.0 M H_2_SO_4_; −0.2–0.8 (vs. SCE)	504.0 F g^−1^, 1.0 A g^−1^	36.3%, 100.0 A g^−1^	0.125–12.5 kW kg^−1^	17.5–6.35 Wh kg^−1^	97.4%, 10,000 cycles, 1.0 A g^−1^	[256]
38	Nanowires-BNC/CP//Nanowires-BNC/CP	H_2_SO_4_/PVA Gels; 1.6 V	255.7 F g^−1^, 1.0 A g^−1^	62.1%, 10.0 A g^−1^	0.2–2.0 kW kg^−1^	22,7–14,1 Wh kg^−1^	90.9%, 5000 cycles, 5,0 A g^−1^	[256]
39	Fe/PANI carbonaceous (cathode)	KOH 6 M; 0,0 a 0.45 V (vs. Ag/AgCl)	1130.8 F g^−1^, 5 mV s^−1^	~18% a 100 mV s^−1^	-	-	78%, 5000 cycles, 5 A g^−1^	[257]
40	Fe/PANI carbonaceous (anode)	KOH 6 M; −1.2–0.0 V (vs. Ag/AgCl)	486.5 F g^−1^, 5 mV s^−1^	~62%, 100 mV s^−1^	-	-	78%, 5000 cycles, 5 A g^−1^	[257]
41	Fe/PANI carbonaceous//Fe/PANI carbonaceous	KOH 6 M; 1.65 V	-	-	0.232–469 kW kg^−1^	41.3 Wh kg^−1^ máx,	72%, 10,000 cycles, 5 A g^−1^	[257]

**Table 3:** Nanostructured PPy in various configurations, evaluated for supercapacitor electrodes, with their performance parameters.

#	Electrodes (Anode//Cathode)	Electrolyte; Potential Windows	Capacitance or Capacity	Current Density	Power Density	Energy Density	Cycling Rate (%)	Ref
01	PPy Nanosheet/rGO//PPy Nanosheet/rGO	KCl 3 M; 0.9 V	290.1 F g^−1^, 0.2 A g^−1^	~85.2%, 12.8 A g^−1^	-	-	97.5%, 20,000 cycles, 2 A g^−1^	[290]
02	PPy/rGO/M-PEFT	Na_2_SO_4_ 1.0 M; −0.4–0.6 V (vs. SCE)	1117 mF cm^−2^/ 329.5 F g^−1^/ 46.6 F g^−1^, 1 mA cm^−2^	80.6%, 50 mA cm^−2^	-	-	~100%, 10,000 cycles, 20 mA cm^−2^	[291]
03	PPy/rGO/M-PEFT//PPy/rGO/M-PEFT	H_2_SO_4_/PVA Gels; 0.0–1.0 V (vs. SCE)	474 mF cm^−2^, 1 mA cm^−2^	73.8%, 50 mA cm^−2^	0.5–25 mW cm^−2^	0.0658–0.0486 mWh cm^−2^	~100%, 10,000 cycles, 20 mA cm^−2^	[291]
04	PPy/rGO/M-PEFY	Na_2_SO_4_ 1.0 M; −0.4–0.6 V (vs. SCE)	175.7 mF cm^−1^/699.6 mF cm^−2^/239.6 F g^−1^/35.0 F cm^−3^, 1 mA cm^−2^	106.7 mF cm^−1^, 424.6 mF cm^−2^, 145.5 F g^−1^, 21.2 F cm^−3^, 13.33 mA cm^−2^	-	-	-	[291]
05	PPy/rGO/M-PEFY//PPy/rGO/M-PEFY	Gel H_2_SO_4_/PVA; 0.0–1.0 V (vs. SCE)	85.3 mF cm^−1^/339.7 mF cm^−2^/116.4 F g^−1^/17.0 F cm^−3^, 1 mA cm^−2^	-	26.5 mW cm^−2^	0.0472 mWh cm^−2^	100%, 10,000 cycles, 6.67 mA cm^−2^	[291]
06	PET/rGO/PPy//PET/rGO/PPy	H_2_SO_4_/PVA Gels; 1.0 V	230 mF cm^−2^ a 1 mV s^−1^/5.5 F cm^−3^, 1.6 mA cm^−3^	-	2 mW cm^−2^	~12 µWh cm^−2^	76%, 6000 cycles, 2 mV s^−1^	[292]
07	Nanotube-PPy@ Nanosheet-G	KCl 3 M; −0.2–0.7 V (vs. SCE)	530 F g^−1^, 1 A g^−1^	~77.3%, 10 A g^−1^	-	-	93%, 1000 cycles, 10 A g^−1^	[293]
08	Nanotube-PPy@ Nanosheet-G // Nanotube-PPy@ Nanosheet-G	LiCl/PVA Gels; 0.8 V	161 mF cm^−2^, 0.18 mA cm^−2^	~79.5%, 1.9 mA cm^−2^	720 µW cm^−2^	11.4 µWh cm^−2^	80%, 5000 cycles, 1.8 mA cm^−2^	[293]
09	PPy/GF	Na_2_SO_4_ 0.5 M; 0.0–0.8 V (vs. Ag/AgCl)	1169 mF cm^−2^, 130 F g^−1^, 5 mA cm^−2^	-	-	-	-	[294]
10	PPy/GF//PPy/GF	ACN-PC-PMMA-LiClO_4_; 1.4 V	89.6 mF cm^−2^, 0.6 mA cm^−2^	16.7%, 3.3 mA cm^−2^	0.39–2.3 mW cm^−2^	24–3.2 µWh cm^−2^	75%, 10,000 cycles, 5 mA cm^−2^	[294]
11	PPy Nanocomposite/G/CNTs//PPy Nanocomposite/G/CNTs	KCl 1 M; 0.8 V	196.7 mF cm^−2^, 0.5 mA cm^−2^	71.3%, 10 mA cm^−2^	8.1 mW cm^−2^	10.9 µWh cm^−2^	95.2%, 5000 cycles, 3 mA cm^−2^	[292]
12	Nanostructures 3D: CuPcTs-PPy/ MWCNTs	H_2_SO_4_ 3 M; 0.0–0.8 V (vs. Ag/AgCl)	488 F g^−1^, 5 A g^−1^	~15%, 20 A g^−1^	-	-	80%, 3000 cycles, 5 A g^−1^	[295]
13	CNF/PPy/ Co_3_[Fe(CN)_6_]_2_	Na_2_SO_4_ 1 M; −0.1–1.1 V (vs. Ag/AgCl)	512 F g^−1^, 0.5 A g^−1^	60.7%, 2.5 A g^−1^	638.8–2894.8 W kg^−1^	102.5–62.3 Wh kg^−1^	87.8%, 2000 cycles, 0.5 A g^−1^	[296]
14	PPy/CCF	Na_2_SO_4_ 1.0 M; 1.0 V	3596 mF cm^−2^, 2 mA cm^−2^	78.98%, 10 mA cm^−2^	-	-	96.5%, 4000 cycles, 0.1 V s^−1^	[297]
15	PPy/CCF//PPy/CCF	LiCl/PVA Gel; 1.2 V	500,06 mF cm^−2^, 5.9 F cm^−3^, 2 mA cm^−2^	~3.4 F cm^−3^, 10 mA cm^−2^	18–84.4 mW cm^−2^	0.68–1.18 mWh cm^−2^	73.6%, 2000 cycles, 0.1 V s^−1^	[12]
16	Nanostructure of Fe_2_O_3_@PPy	Na_2_SO_4_ 1 M; 0.8 V	560 F g^−1^, 5 A g^−1^	~62.5%, 40 A g^−1^	-	-	97.3%, 20,000 cycles, 40 A g^−1^	[298]
17	N-CNTs -PPy/MnO_2_	Li_2_SO_4_ 0.5 M; 0.0–1.0 V (vs. SCE)	366.5 F g^−1^, 0.5 A g^−1^	67%, 25 A g^−1^	500–10,000 W kg^−1^	12.5–6.6 Wh kg^−1^	91.2%, 6000 cycles, 5 A g^−1^	[299]
18	N-CNTs//N-CNTs PPy/MnO_2_	Li_2_SO_4_ 0.5 M; −0.8–1.0 V (vs. SCE)	48.6 F g^−1^/560 mF cm^−2^, 1 mA cm^−2^	73.7%, 40 mA cm^−2^	224 W kg^−1^	20.9 Wh kg^−1^	91.6%, 5000 cycles, 5 mA cm^−2^	[299]
19	V_2_O_5_/NG-gel Nanowires-PPy	LiCl 8 M; 1.0 V	710.5 F g^−1^, 0.5 A g^−1^	~50%, 10 A g^−1^	250–5000 W kg^−1^	98.6–50 Wh kg^−1^	95%, 20,000 cycles, 10 A g^−1^	[300]
20	NiCo_2_S_4_@Ni(OH)_2_@PPy Nanotube	KOH 2 M; 0.0–0.4 V (vs. SCE)	2838.8 F g^−1^/9112.5 mF cm^−2^, 5 mA cm^−2^	~43%, 60 mA cm^−2^	-	-	96.2%, 3000 cycles, 60 mA cm^−2^	[300]
21	AC//NiCo_2_S_4_@Ni(OH)_2_@PPy Nanotube	KOH 2 M; 1.6 V	3246.8 mF cm^−2^, 5 mA cm^−2^	~35%, 60 mA cm^−2^	34.67 W kg^−1^	120.127 Wh kg^−1^	98.87%, 30,000 cycles, 60 mA cm^−2^	[301]
22	CF//CoSe_2_@PPy Nanofibers	Gel LiCl/PVA; 1.6 V	226 mF cm^−2^, 18 mA cm^−2^	-	0.42–14 mW cm^−2^	2.63–0.08 mWh cm^−2^	80.1%, 15,000 cycles, 106 mA cm^−2^	[302]
23	Graphite/ Nanobelt- MnCo_2_O_4_@ Nanosheet-PPy	KOH 6 M; −0.1–0.4 V (vs. SCE)	2364 F g^−1^, 1 A g^−1^	55.2%, 50 A g^−1^	-	-	85.3%, 1000 cycles, 30 A g^−1^	[303]
24	a-MEGO//Graphite/ Nanobelt- MnCo_2_O_4_@ Nanosheet-PPy	KOH 6 M; 1.6 V	288.8 F g^−1^, 0.5 A g^−1^	-	16.1 kW kg^−1^	25.7 Wh kg^−1^	85.5%, 10,000 cycles, 5 A g^−1^	[303]
25	rGO@PPy/Fe_3_O_4_ Nanosheet	KOH 6 M; 0.0–0.36 V (vs. SCE)	1006 F g^−1^, 1 A g^−1^	~74.5%, 20 A g^−1^	-	-	85%, 5000 cycles, 10 A g^−1^	[304]
26	3D nanostructured GO-sulfonated/ Ni(OH)_2_/PPy	KOH 6 M; 0.0–0.4 V (vs. SCE)	1632 F g^−1^, 1 A g^−1^	-	-	-	86%, 1000 cycles, 10 A g^−1^	[305]
27	AC//3D nanostructured GO-sulfonated/ Ni(OH)_2_/PPy	Gel KOH/PVA; 1.6 V	224 F g^−1^, 1 A g^−1^	~61.2%, 10 A g^−1^	0.8–7.97 kW kg^−1^	79.6–48.7 Wh kg^−1^	60%, 5000 cycles, 10 A g^−1^	[305]
28	Nanoparticles Hydrogel -Au/CNT/PAM@PPy// Nanoparticles Hydrogel -Au/CNT/PAM@PPy	Hydrogel of Na_2_SO_4_/ Nanoparticles- Au/PAM	885 mF cm^−2^, 1 mA cm^−2^	~66%, 9 mA cm^−2^	0.5–4.9 mW cm^−2^	123–81 µWh cm^−2^	93%, 10,000 cycles, 10 mA cm^−2^	[306]
29	PPy/3D-rGO/Au nanoporous // PPy/3D-rGO/Au nanoporous	HClO_4_/PVA; 1.7 V	29.21 mF cm^−2^, 0.07 mA cm^−2^/386.94 F g^−1^, 0.92 A g^−1^	48%, 2.8 mA cm^−2^	2.36 mW cm^−2^	11.72 µWh cm^−2^/98.48 mWh cm^−3^	85.9%, 10,000 cycles, 5 mA cm^−2^	[307]
30	GO-exfoliated/nanoparticles-PPy-Ag	H_2_SO_4_ 1 M; 0.8 V	287.5 F g^−1^, 1 A g^−1^	87.4%, 10 A g^−1^	399–3994 W kg^−1^	25.5–22.3 Wh kg^−1^	93%, 5000 cycles, 2 A g^−1^	[308]
31	CC/CG hollow fibers/PPy//CC/CG hollow fibers/PPy	Gel H_2_SO_4_/PVA; 1 V	341.64 F g^−1^;785.78 mF cm^−2^, 4 mA cm^−2^	>85%, 10 mA cm^−2^	100 mW cm^−3^	0.374 mWh cm^−3^	90%, 100 cycles	[309]
32	Cellulose Nanofibers/rGO@PPy microfibers//Cellulose Nanofibers/rGO@PPy microfibers	H_2_SO_4_ 1 M; 0.8 V	334 mF cm^−2^, 0.1 mA cm^−2^	~98.8%, 1 mA cm^−2^	20 µW cm^−2^	7.4 µWh cm^−2^	100%, 2000 cycles, 1 mA cm^−2^	[310]
33	cellulose nanofibers/rGO@PPy microfibers//cellulose Nanofibers/rGO@PPy microfibers	Gel H_3_PO_4_/PVA; 0.8 V	218 mF cm^−2^; 218 mF cm^−3^, 0.1 mA cm^−2^	-	-	4.8 µWh cm^−2^	95.2%, 3000 cycles, 1 mA cm^−2^	[310]
34	CA@PPy// CA@PPy	H_2_SO_4_ 1 M; −0.2–0.6 V (vs. Ag/AgCl)	268.5 F g^−1^, 0.5 A g^−1^	82.4%, 10 A g^−1^	450.4–8018.2 W kg^−1^	23.8–19.6 Wh kg^−1^	88%, 10,000 cycles, 10 A g^−1^	[311]
35	Bilayer: rGO/PPy|MWCNTs/rGO/cellulose nanocrystal	KCl 1.0 M; 1.0 V	~330 F g^−1^, 0.5 A g^−1^	92.5%, 6 A g^−1^	2889.9 W kg^−1^	44.6 Wh kg^−1^	90%, 10,000 cycles, 200 mV s^−1^	[312]
36	PPy nanofibers hydrogel	H_2_SO_4_ 1 M; 0.0–0.8 V (vs. Ag/AgCl)	328 F g^−1^, 1 A g^−1^	75%, 8 A g^−1^	-	-	90%, 3000 cycles, 2 A g^−1^	[312]
37	PPy Hydrogel//PPy Hydrogel	Gel H_2_SO_4_/PVA; 0.8 V	~170 F g^−1^, 1 A g^−1^	~59%, 8 A g^−1^	0.27–2.31 kW kg^−1^	4.13–2.24 Wh kg^−1^	90%, 3000 cycles, 1 A g^−1^	[313]
38	PPy nanofibers hydrogel	H_2_SO_4_ 1 M; 0.0–0.8 V (vs. Ag/AgCl)	324 F g^−1^/486 mF cm^−2^, 1.25 mA cm^−2^	~53.5%, 12.5 mA cm^−2^	-	-	-	[314]
39	PPy nanofibers hydrogel// PPy nanofibers hydrogel	Gel H_2_SO_4_/PVA; 0.8 V	168 F g^−1^/~505 mF cm^−2^, 2.0 mA cm^−2^	~50%, 20.0 mA cm^−2^	0.2–8 mW cm^−2^	44.9–22.2 µWh cm^−2^	53%, 10,000 cycles	[314]
40	PPy nanofibers hydrogel -TB	H_2_SO_4_ 1 M; −0.2–0.8 V (vs. Ag/AgCl)	707 F g^−1^, 0.5 A g^−1^	~76.4%, 5 A g^−1^	-	-	-	[315]
41	NS-C nanosphere-PPy	KOH 6 M; −0.8–0.0 V (vs. Hg/HgO)	416 F g^−1^, 0.2 A g^−1^	75.2%, 10 A g^−1^	-	-	95.8%, 5000 cycles, 1 A g^−1^	[316]
42	NS-C nanospheres PPy // NS-C nanospheres PPy	KOH 6 M; 1.0 V	-	-	100 W kg^−1^	18.1 Wh kg^−1^	98.3%, 5000 cycles, 1 A g^−1^	[316]

**Table 4:** PEDOT and other nanostructured PTh derivatives, in various configurations were evaluated for supercapacitor electrodes with their performance parameters.

#	Electrodes (Anode//Cathode)	Electrolyte; Potential Window	Capacitance or Capacity	Current Density	Power Density	Energy Density	Cycling Rate (%)	Ref
01	CC/PEDOT:PSS/ rGO (porous)	H_3_PO_4_/PVA (gel); 1.8 V	170 F g^−1^/3000 mF cm^−2^, 10 mV s^−1^	-	-	-	~100%, 2000 cycles, 400 mV s^−1^	[340]
02	CC/PEDOT:PSS/ rGO //CC/PEDOT:PSS/rGO (porous)	H_3_PO_4_/PVA (gel); 1.8 V	82 F g^−1^, 100 mV s^−1^	~78%, 1000 mV s^−1^	4460 W kg^−1^	11.0 Wh kg^−1^	-	[340]
03	MWCNTs/PEDOT (sponge shape)	H_2_SO_4_ 1 M; −0.2 to 0.8 V (vs. Ag/AgCl)	147 F g^−1^, 0.5 A g^−1^	70%, 10 A g^−1^	-	-	94.7%, 3000 cycles, 1 A g^−1^	[341]
04	MWCNTs/PEDOT (sponge shape) // MWCNTs/PEDOT (sponge shape)	H_2_SO_4_/PVA (gel); 1.4 V	51 F g^−1^, 0.5 A g^−1^	-	1.2 kW kg^−1^	12.6 Wh kg^−1^	-	[341]
05	SWCNTs (Nanostructure)/ PEDOT:PSS/CuHCF	H_2_SO_4_ 1 M; −0.2–0.8 V (vs. Ag/AgCl)	969.8 mF cm^−2^, 5 mV s^−1^	~69.9%, 500 mV s^−1^	-	-	95%, 15,000 cycles, 10 mA cm^−2^	[342]
06	Mo-WO_3_/SWCNTs//SWCNTs (nanostructured)/ PEDOT:PSS/CuHCF	H_2_SO_4_ 1 M; 1.4 V	530.3 mF cm^−2^, 10 mV s^−1^	75.1%, 1000 mV s^−1^	4.25–10.79 kW L^−1^	30.08–29.01 Wh L^−1^	88.3%, 10,000 cycles, 10 mA cm^−2^	[342]
07	CNTs@P3MT	TEATFB 1 M: −1.7 to 1.5 V (vs. Ag/AgCl)	~3110 mF cm^−2^, 5 mA cm^−2^	58%, 200 mA cm^−2^	-	-	-	[343]
08	CNTs@P3MT//CNTs	TEATFB 1 M: −1.7 to 1.5 V (vs. Ag/AgCl)	~640 mF cm^−2^, 5 mA cm^−2^	~50%, 500 mA cm^−2^	1.75 W cm^−2^	1.08 mWh cm^−2^	92%, 5000 cycles, 100 mA cm^−2^	[343]
09	Nanowire V_2_O_5_@PEDOT//Nanowire V_2_O_5_@PEDOT	Na_2_SO_4_ 1 M; 2 V	614 F g^−1^ a 0.5 A g^−1^	~49%, 10 A g^−1^	250 W kg^−1^	85 Wh kg^−1^	122%, 50,000 cycles, 10 A g^−1^	[344]
10	Nanowire of (NH_4_)_2_V_6_O_16_/PEDOT	KCl 2 M; 0.0–0.9 V (vs. SCE)	202 F g^−1^, 3 mV s^−1^	65%, 100 mV s^−1^	-	-	-	[345]
11	AC//nanowire of (NH_4_)_2_V_6_O_16_/PEDOT	KCl 2 M; 1.8 V	45.9 F g^−1^/62 mF cm^−1^, 0.67 A g^−1^	40%, 20 A g^−1^	600–18,000 W kg^−1^/ 0.8–24.3 mW cm^−2^	20.7–8.2 Wh kg^−1^/ 27.9–11.2 µWh cm^−2^	87%, 1000 cycles, 2 A g^−1^	[345]
12	Nanoparticles- RuO_2_·1.18H_2_O/nanosheets- PEDOT:PSS	H_2_SO_4_ 1 M; −0.2–0.8 V (vs. SCE)	630 F g^−1^, 15 A g^−1^	-	-	-	-	[346]
13	Nanoparticles- RuO_2_·1.18H_2_O/ nanosheets- PEDOT:PSS // Nanoparticles- RuO_2_·1.18H_2_O/ nanosheets- PEDOT:PSS	H_2_SO_4_ 1 M; 1.0 V	540 F g^−1^, 2 A g^−1^	94%, 20 A g^−1^	500–5000 W kg^−1^	19–18 Wh kg^−1^	91.5%, 5000 cycles, 10 A g^−1^	[346]
14	Nanotube-Ni@ MnO_2_@PEDOT	LiCl 1 M; 0.0–0.7 V (vs. SCE)	442.85 F g^−1^, 2.5 A g^−1^/88.71 mF cm^−2^, 0.5 mA cm^−2^	~55.8%, 10 A g^−1^	-	-	80.37%, 15,000 cycles, 100 mV s^−1^	[347]
15	AC//Nanotube-Ni@ MnO_2_@PEDOT	LiCl 1 M; 1.7 V	1.47 F cm^−3^, 4 mA cm^−2^	~61%, 10 mA cm^−2^	84.96 mW cm^−3^	0.59 mWh cm^−3^	81.16%, 10,000 cycles, 100 mV s^−1^	[347]
16	Ag nanowire/WO_3_@PEDOT:PSS (nanotubes)	LiClO_4_ 1 M; −2.4–0.0 V (vs. Ag/AgCl)	471.0 F g^−1^, 1 A g^−1^	~85%, 16 A g^−1^	7.7–19.1 kW kg^−1^	52.6–44.67 Wh kg^−1^	79.5%, 50,000 cycles	[348]
17	Nanosheets of MXeno Ti_3_C_2_T_x_/PEDOT:PSS	H_2_SO_4_ 1 M; −0.7–0.2 V (vs. Ag/AgCl)	614.5 F cm^−3^/258.2 F g^−1^, 5 mV s^−1^	~61%, 1000 mV s^−1^	-	-	-	[349]
18	Nanosheets of MXeno Ti_3_C_2_T_x_/PEDOT:PSS// Nanosheets of MXeno Ti_3_C_2_T_x_/PEDOT:PSS	Gel H_2_SO_4_/PVA; 0.6 V	361.4 F cm^−3^, 2 mV s^−1^	~53%, 200 mV s^−1^	142.16–8249 mW cm^−3^	7.13–5.04 mWh cm^−3^	~95%, 10,000 cycles, 100 mV s^−1^	[349]
19	MXeno Mo_1,33_C/PEDOT nanosheet	H_2_SO_4_ 1 M; −0.35–0.30 V (vs. Ag/AgCl)	452 F g^−1^/1310 F cm^−3^, 2 mV s^−1^	~45%, 1000 mV s^−1^	-	-	-	[350]
20	MXeno Mo_1,33_C/PEDOT nanosheets// MXeno Mo_1,33_C/PEDOT nanosheets	Gel H_2_SO_4_/PVA; 0.0–0.65 V (vs. Ag/AgCl)	568 F cm^−3^, 0.5 A cm^−3^	74.1%, 30 A cm^−3^	1947.0 mW cm^−3^	33.2 mWh cm^−3^	90%, 10,000 cycles, 5 A cm^−3^	[350]
21	PVA-GQDs-Co_3_O_4_@PEDOT (nanofibers)	H_2_SO_4_ 1 M; 0.0–1.0 V (vs. Ag/AgCl)	361.97 F g^−1^, 25 mV s^−1^	~89%, 200 mV s^−1^	496.10–2396.99 W kg^−1^	19.98–16.51 Wh kg^−1^	96%, 1000 cycles, 100 mV s^−1^	[351]
22	PVA-GO-MnO_2_ (microfibers)/ PEDOT (nanoparticles)//PVA-GO-MnO_2_ (microfibers)/ PEDOT (nanoparticles)	KCl 1.0 M; 0.0–1.0 V (vs. Ag/AgCl)	144.66 F g^−1^, 50 mV s^−1^	~55%, 200 mV s^−1^	243.72–475.09 W kg^−1^	9.60–9.21 Wh kg^−1^	91.18%, 1000 cycles, 100 mV s^−1^	[352]
23	Multicapas Co_9_S_8_/PEDOT:PSS- carbonizado/ rGO	KOH 2 M; 0.0–0.45 V (vs. Ag/AgCl)	788.9 F g^−1^, 1.0 A g^−1^	~56.9%, 20.0 A g^−1^	-	-	>100%, 10,000 cycles, 15.0 A g^−1^	[353]
24	AC//multilayer Co_9_S_8_/PEDOT:PSS/ rGO	KOH 2 M; 1.6 V	55.0 F g^−1^, 0.5 A g^−1^	-	400.9–8030.7 W kg^−1^	19.6–8.7 Wh kg^−1^	94.2%, 10,000 cycles, 5 A g^−1^	[353]
25	Nanocomposite of PEDOT@AQ-COF	H_2_SO_4_ 1 M; −0.2–0.6 V (vs. Ag/AgCl)	1663 F g^−1^, 1 A g^−1^	60%, 500 A g^−1^	-	-	~118%, 10,000 cycles, 50 A g^−1^	[354]
26	PEDOT nanowires	H_2_SO_4_ 1 M/PDA; −0.4–0.5 V (vs. Ag/AgCl)	667.5 mF cm^−2^, 1 mA cm^−2^	~74.9%, 20 mA cm^−2^	-	-	88.1%, 5000 cycles, 20 mA cm^−2^	[355]
27	PEDOT nanowires//PEDOT nanowires	Gel H_2_SO_4_/PVA/PDA; 0.9 V	413.5 mF cm^−2^, 1 mA cm^−2^	~74%, 50 mA cm^−2^	0.22–16.8 mW cm^−2^	48.3–19.1 µWh cm^−2^	-	[355]

**Table 5:** Other polymeric nanostructures evaluated for supercapacitor electrodes, with their performance parameters.

#	Electrodes (Anode//Cathode)	Electrolyte; Potential Window	Capacitance or Capacity	Current Density	Power Density	Energy Density	Cycling Rate (%)	Ref
01	rGO/CNT@PMTA//rGO/CNT@PMTA	KOH 6 M; 1.0 V	658 F g^−1^, 0.5 A g^−1^	90.5%, 2 A g^−1^	~120 W kg^−1^	23 Wh kg^−1^	92%, 1000 cycles, 2 A g^−1^	[370]
02	rGO-aniline/ QDs-P*o*PD//rGO-aniline/ QDs-P*o*PD	H_2_SO_4_ 1 M; 1.0 V	420 F g^−1^, 0.5 A g^−1^	~61%, 20 A g^−1^	125.2–5027.6 W kg^−1^	14.6–8.1 Wh kg^−1^	90%, 5000 cycles, 1 A g^−1^	[371]
03	ACM/MWCNTs@ PDAA	Et_4_NBF_4_ 1 M in CH_3_CN; −1.8–0.45 V (vs. Ag/AgCl)	20.2 F cm^−3^, 1 mA cm^−2^	58%, 20 mA cm^−2^	-	-	-	[372]
04	ACM/MWCNTs@ PANI//ACM/MWCNTs@ PDAA	Quasi-solid polymeric electrolyte ACM/Et_4_NBF_4_-CH_3_CN; 2.7 V	2.2 F cm^−3^, 1 mA cm^−2^	86%, 20 mA cm^−2^	0.021–0.50 mW cm^−3^	2.14–1.13 mWh cm^−3^	80.3%, 5000 cycles, 4 mA cm^−2^	[372]
05	MoO_3_/PPy nano-ribbons with nano-wires-PANI	H_2_SO_4_ 0.5 M; −0.2–0.8 V (vs. SCE)	1315 F g^−1^, 0.5 A g^−1^	~51.1%, 10 A g^−1^	-	-	86%, 20,000 cycles, 10 A g^−1^	[373]
06	MoO_3_/PPy nano-ribbons with nano-wires-PANI// MoO_3_/PPy nano-ribbons with nano-wires-PANI	H_2_SO_4_ 0.5 M; 1.0 V	908.1 F g^−1^, 0.5 A g^−1^	~55.5%, 20 A g^−1^	250 W kg^−1^	63 Wh kg^−1^	100%, 3000 cycles, 2 A g^−1^	[373]
07	PEDOT/PANI/Phytic acid hydrogels	H_2_SO_4_ 1 M; −0.2–0.8 (vs. Ag/AgCl)	112.6 F g^−1^, 5 mV s^−1^	-	-	-	80.8%, 5000 cycles, 7.5 A g^−1^	[374]
08	PEDOT/PANI/Phytic acid hydrogels// PEDOT/PANI/Phytic acid hydrogels	H_2_SO_4_(Gel)/PVA; 1.0 V	242.2 mF cm^−2^; 3.5 F cm^−3^, 1 mA cm^−2^	~50%, 15 mA cm^−2^	62.39–937 W kg^−1^	0.48–0.25 mWh cm^−3^; 4.20–2.18 Wh kg^−1^	82,5%, 5000 cycles, 15 mA cm^−2^	[374]
09	Hierarchically porous CNTs derived from MnO_2_@PPy-co-PANI	KOH 6 M; −1.1–0.1 V (vs. Hg/HgO)	286 F g^−1^, 0.1 A g^−1^	~71%, 50 A g^−1^	-	-	100%, 10,000 cycles, 20 A g^−1^	[375]
10	NHCS/ Nano-crystals of FeCo@ F-enriched C nano-layer derived from P(ANI-co-F-ANI) (NHCS/FeCo@FC)	KOH 6 M; −1.0–0.0 V (vs. Ag/AgCl)	302 F g^−1^, 0.2 A g^−1^	61.4%, 10 A g^−1^	-	-	100%, 5000 cycles, 5 A g^−1^	[376]
11	NHCS/FeCo@FC//NHCS/FeCo@FC	KOH/PVA; 1.5 V	51.2 F g^−1^, 0.2 A g^−1^	81.33%, 10 A g^−1^	11.1 W kg^−1^	15.3 Wh kg^−1^	~93%, 10,000 cycles, 2 A g^−1^	[376]
12	2D-CAP nano-porous P(3-TBQP)	KCl 2 M; 0.0–1.0 V (vs. SCE)	240 F g^−1^, 1 A g^−1^	-	-	-	-	[377]
13	AC//2D-CAP nano-porous P(3-TBQP)	KCl 2 M; 1.6 V	233 F g^−1^, 1 A g^−1^	~59%, 10 A g^−1^	8.7 kW kg^−1^	23 Wh kg^−1^	~80%, 10,000 cycles, 2 A g^−1^	[377]
14	2D-CMP poly(1,3,5-triethynyl benzene-ferrocene)/rGO	H_2_SO_4_ 1 M; 0.0–0.8 V (vs. Ag/AgCl)	470 F g^−1^, 0.5 A g^−1^	~66%, 10 A g^−1^	-	-	-	[378]
15	2D-CMP poly(1,3,5-triethynyl benzene-ferrocene)/rGO// 2D-CMP poly(1,3,5-triethynyl benzene-ferrocene)/rGO	H_2_SO_4_ 1 M; 1.0 V	231 F g^−1^; 238 mF cm^−3^, 0.5 A g^−1^	~58%, 10 A g^−1^	124–2532 W kg^−1^	8–5 Wh kg^−1^	91%, 8000 cycles, 5 A g^−1^	[378]
16	Hollow spheres with nano-layers of CMP-BPPB//Hollow spheres with nano-layers of CMP-BPPB	H_2_SO_4_ 1 M; −0.5–0.5 V (vs. Ag/AgNO_3_)	220 F g^−1^, 0.5 A g^−1^	49%, 20 A g^−1^	-	-	85%, 10,000 cycles, 6 A g^−1^	[379]
17	3D-CMP PAQTA	H_2_SO_4_ 0.5 M; 0.2–0.8 V (vs. Ag/AgCl)	576 F g^−1^, 1.0 A g^−1^	~71.2%, 10.0 A g^−1^	-	-	~80%, 6000 cycles, 2 A g^−1^	[380]
18	AC//3D-CMP PAQT	H_2_SO_4_ 0.5 M; 1.6 V (vs. Ag/AgCl)	168 F g^−1^, 1.0 A g^−1^	~63.1, 10.0 A g^−1^	1300 W kg^−1^	60 Wh kg^−1^ máx.	~95.5%, 2000 cycles, 2.0 A g^−1^	[380]
19	N,O-PC foam derived from PAQ	H_2_SO_4_ 1 M; 0.0 a 1.0 V (vs. Ag/AgCl)	410 F g^−1^, 1 A g^−1^	~55%, 50 A g^−1^	-	-	-	[381]
20	N,O-PC foam derived from PAQ//N,O-PC foam derived from PAQ	H_2_SO_4_ 1 M; 1.0 V	321 F g^−1^, 1 A g^−1^	69%, 50 A g^−1^	-	-	98%, 15,000 cycles, 5 A g^−1^	[381]
21	N,O-PC foam derived from PAQ//N,O-PC foam derived from PAQ	Li_2_SO_4_ 1 M; 1.6 V	216 F g^−1^, 0.5 A g^−1^	56%, 20 A g^−1^	0.4–8.0 kW kg^−1^	15.91–10.67 Wh kg^−1^	96%, 15,000 cycles, 5 A g^−1^	[381]
22	Microporous CTF-TCNQ	KOH 1 M; −0.8–1.0 V (vs. Hg/HgO)	383 F g^−1^, 0.2 A g^−1^	~45%, 10 A g^−1^	-	-	~100%, 10,000 cycles, 10 mV s^−1^	[382]
23	Microporous CTF-TCNQ//Microporous CTF-TCNQ	IL EMIMBF_4_; 3.5 V	~100 F g^−1^, 0.1 A g^−1^	~40%, 10 A g^−1^	8750 W kg^−1^	42,8 Wh kg^−1^	92%, 5000 cycles, 7 A g^−1^	[382]
24	3D-PC derived from PIn nanospheres	H_2_SO_4_ 1,0 M; −0,2–1.0 V (vs. SCE)	328 F g^−1^, 1.0 A g^−1^	-	-	-	-	[383]
25	3D-PC derived from PIn nanospheres// 3D-PC derived from PIn nanospheres	H_2_SO_4_ 1.0 M; 1.2 V	~295 F g^−1^, 0.2 A g^−1^	66%, 10 A g^−1^	120–6000 W kg^−1^	15–9.4 Wh kg^−1^	91.1%, 10,000 cycles, 10 A g^−1^	[383]

**Table 6:** Nanostructured NIBP in various configurations, evaluated for rechargeable battery electrodes, with their performance parameters.

#	Active Cathode Material; form (Mass Loading of Active Materials ^a^)	Active Anode Material; form (Mass Loading of Active Materials ^a^)	Electrolyte	Medium Operating Potential; Potential Window/V	Specific Initial Discharge Capacity ^b^; Coulombic Efficiency	Long-Term Performance: Reversible Capacity ^b^; Cycles; Speed or Current Density; Coulombic Efficiency; Energy Density	Speed Capability: Specific Capabilities; Velocities and Current Densities	Ref
01	LiMn_2_O_4_	LiTi_2_(PO_4_)_3_@NC; nanoparticles (5–8)	Li2SO4 in saturated aqueous solution	1.5; 0–1.85 ^g^	115.2; -	94.6; 100; 2 C; ~100%; 141.9	122.4–95.3; 0.2 C–10 C	[407]
02	Li	NSBDC (~2.21)	LiPF_6_ 1 M EC:DEC (1:1 *v*/*v*)	-; 0.01–3.0 ^g^	1513; ~75.8%	357; 200; 5.0 ^c^; ~96%; -	1111–363; 0.1–5.0 ^c^	[408]
03	Li	IGR-MnO-NC (~1)	LiPF_6_ 1 M EC:DMC (1:1 *v*/*v*)	-; 0.01–3.0 ^g^	1802; 56%	904; 500; 0.5 ^c^; ~99%; -	1055–547; 0.1–2.0 ^c^	[409]
04	Li	MnO@NC; nanocapsules (1.0)	LiPF_6_ 1 M EC:DMC (1:1 *v*/*v*)	0.49; 0.01–3.0 ^g^	1139; ~54%	624; 1000; 1000 ^d^; ~100%; -	762–458; 100–5000 ^d^	[410]
05	LiFePO_4_	nO@NC; nanocapsules (1.0)	LiPF_6_ 1 M EC:DMC (1:1 *v*/*v*)	-; 0.01–3.8 ^g^	127; -	60; 200; 85 ^d^; -; -	-	[410]
06	Li	CNTs@Si@PANI	LiPF_6_ 1 M EC:DEC:DMC (1:1:1 *v*/*v*)	0.2; 0.01–2.0 ^g^	1954; 65.0%	727; 100; 0,1 ^c^; ~99%; -	720–258; 0.1–1.0 ^c^	[411]
07	Li	ZnFe_2_O_4_@PANI; nanofibber	LiPF_6_ 1 M EC:DEC:EMC (1:1:1 *v*/*v*)	0.7; 0.01–3.0 ^g^	1470; 85.7%	1142; 50; 50 ^d^; -; 799.4	1470–539; 50–5000 ^d^	[412]
08	Li	HCs@Si@C (1.0)	LiPF_6_ 1 M	-; 0.01–3.0 ^g^	2708; 67.7%	1216.8; 600; 420 ^d^; 99.5%; -	2055.4–827; 0.1–4.2 ^c^	[413]
09	LiFePO_4_	HCs@Si@C (1.0)	LiPF_6_ 1 M	~2.7; 1.5–3.6 ^g^	111.6 ^f^; ~78.2%	117.7 ^f^; 100; 0.1 ^c^; ~99%; 317.8 ^f^	133–76 ^f^; 0.1–1 ^c^	[413]
10	3D EDA-CNTs/S@ NIBP; coated nanotube networks (2.0)	Li	LiTFSI 1 M DME:DOL (1:1 *v*/*v*)	~2.1; 1.4–2.8 ^g^	1215 ^f^; ~90%	975 ^f^; 200; 0.2 C; ~100%; -	1259–462 ^f^; 0.2 C–3.0 C	[414]
11	C-porous@ S-NIBP; coated nanospheres (2.1 ± 0.2)	Li	LiTFSI 1 M and LiNO_3_ 0.2 M DME:DOL (1:1 *v*/*v*)	-; 1.5–2.8	1182.5 ^f^; -	494.5 ^f^; 500; 2 ^c^; ~98%; -	1131.2–665.2 ^f^; 0.2–5 ^c^	[415]
12	S-impregnated NP-C/HKUST-1MOF; 3D monolithic structure (18.8)	Li	LiTFSI 1 M y LiNO_3_ 0.1 M DME:DOL (1:1 *v*/*v*)	-; 1.7–2.8 ^g^	923 ^f^ (17.6 ^k^; 0.2 C); -	757 ^f^; 300; 0.2 C; 98.83%; 2843.4 ^l^	1377–541 ^f^; 0.05 C–0.60 C	[416]
13	PB@NIBP; coated cubes (1.5)	Na	NaPF_6_ 1 M EC:DEC:DMC (1:1:1 *v*/*v*)	-; 2.0–3.6 ^h^	~108 ^f^; 95.4%	101.1 ^f^; 500; 100 ^d^; ~100%; -	116.5–63.3 ^f^; 60–5000 ^d^	[417]
14	Na	NiS2@NC@NC; coated stick	NaClO_4_ 1 M PC	-; 0.01–3.0 ^h^	840.7; 79.65%	580.8; 100; 0.1 ^c^; 100%; -	694–448; 0.05–1.6 ^c^	[418]
15	NIBP; nano-fibers (3.0)	K	KPF_6_/PMMA (Gel)	~3.2; 2.0–4.0 ^i^	-; ~99%	98% *; 100; 50 ^d^; 99.3%; ~442 ^f^	138–95 ^f^; 10–200 ^d^	[419]
16	NIBP; nano-pillars (0.8)	Zn	ZnCl_2_ 2 M y NH_4_Cl 3 M	-; 0.7–1.7 ^j^	-	~130 ^f^; 1000; 8 ^c^; 100%; 233.4 ^f^,	203.5–118.7 ^f^; 0.5–16 ^c^	[420]
17	CF@NIBP; coated fibers	Zn	ZnCl_2_ 1 M	-; 0.7–1.7 ^j^	-	80% *; 100; 1 C; -; 389	165–31.4 ^f^; 1 C–600 C	[421]

^a^ Mass charge in mg cm^−2^ unless otherwise specified/^b^ Specific capacity in mAh g^−1^ relative to the mass of active materials of the anode unless otherwise indicated/^c^ Current density expressed in A g^−1^/^d^ Current density expressed in mA g^−1^/^e^ Reported in Wh kg^−1^/^f^ With respect to the mass of active materials of the cathode/^g^ (vs. Li^+^/Li)/^h^ (vs. Na^+^/Na)/* Regarding initial capacity download.

**Table 7:** PPy nanostructured in various configurations, evaluated for rechargeable battery electrodes, with their performance parameters.

#	Active Cathode Material; form (Mass Loading of Active Materials ^a^)	Active Anode Material; form (Mass Loading of Active Materials ^a^)	Electrolyte	Medium Operating Potential; Potential Window/V	Specific Initial Discharge Capacity ^b^; Coulombic Efficiency	Long-Term Performance: Reversible Capacity ^b^; Cycles; Speed or Current Density; Coulombic Efficiency; Energy Density	Speed Capability: Specific Capabilities; Velocities and Current Densities	Ref
01	Li	Sb@NC; nanorods (1.2)	LiPF_6_ 1 M EC:DMC:EMC (1:1:1 *v*/*v*)	0.79; 0.01–3.0 ^g^	831.5; 78.3%	395; 3000; 2 ^c^; ~100%; 312.1	641.2–343.3; 0.2–20 ^c^	[422]
02	Li	MoS_2_/NC; nanowires	LiPF_6_ 1 M EC:DMC:EMC (1:1:1 *v*/*v*)	-; 0.01–3.0 ^g^	1024; 72.6%	520; 500; 5 ^c^; ~100%; -	822–453; 0.2–10 ^c^	[423]
03	Li	MoS_2_@NC; nanospheres (1.6)	LiPF_6_ 1 M EC:DMC (1:1 *v*/*v*)	-; 0.01–3.0 ^g^	1344; 75%	530; 500; 2 ^c^; ~100%; -	1112–616; 0.1–2 ^c^	[424]
04	Li	MoS_2_@PPy (2.5)	LiPF_6_ 1 M EC:DMC (1:1 *v*/*v*)	-; 0.01–3.0 ^g^	1427; 79.0%	1012; 200; 200 ^d^; 99%; -	1062–600; 200–4000 ^d^	[425]
05	Li	HUTS@C	LiPF_6_ 1 M EC:DEC (1:1 *v*/*v*)	1.75; 1.0–3.0 ^g^	227.2; 80.4%	165.1; 200; 1 C; ~98%; ~289	181.0–111.7; 2 C–10 C	[426]
06	Li	MOF-Co@NC/G; encapsulated nanocrystals (1.5)	LiPF_6_ 1 M EC:DMC (1:1 *v*/*v*)	-; 0.01–3.0 ^g^	1978; 60.6%	739; 2000; 5 ^c^; ~100%; -	1301–494; 0.1–40 ^c^	[427]
07	Li	Li_4_Ti_5_O_12_/C doped with N and TiO_2_; nanorods	LiPF_6_ 1 M EC:DMC (1:1 *v*/*v*)	1.55; 1.0–3.0 ^g^	-	~150; 3000; 10 C; -; -	1963–105.5; 0.5 C–100 C	[428]
08	LiFePO_4_	Li_4_Ti_5_O_12_/C doped with N and TiO_2_; nanorods	LiPF_6_ 1 M EC:DMC (1:1 *v*/*v*)	~1.8; 0.5–3.0 ^g^	150.7; -	~148; 100; 10 C; -; ~266	-	[428]
09	Li	H-TiO_2_@SnS_2_@PPy; encapsulated nanosheets	LiPF_6_ 1 M EC:DMC (1:1 *v*/*v*)	-; 0.01–3.0 ^g^	~967; 71.2%	508.7; 2000; 2.0 ^c^; ~100%; -	701.2–356.2; 0.2–10 ^c^	[429]
10	Li	3D NCW@Fe_3_O_4_/ NC (0.20)	LiPF_6_ 1 M EC:DMC (1:1 *v*/*v*)	0.01–3.0 ^g^	2867; 55.3%	1741; 600; 1 C; 99%; -	1309–723; 0.1 C–10 C	[430]
11	S/CoS@PPy; coated buckets (1.4–1.2)	Li	LiTFSI 1 M DME:DOL (1:1 *v*/*v*)	2.1; 1.7–2.8 ^g^	1165 ^f^; ~95%	700 ^f^; 500; 0.2 C; 99%; 1470	~1000–450 ^f^; 0.2 C–4 C	[431]
12	S-PPy at PCN-224; nanocages (0.8–1.4)	Li	LiTFSI 1 M, LiNO_3_ 0.1 M DME:DOL (1:1 *v*/*v*)	2.2; 1.8–2.7 ^g^	1330 ^f^; -	440 ^f^; 1000; 10.0 C; ~99%; -	1330–640 ^f^; 0.5 C–5.0 C	[432]
13	S@MnO_2_@PPy; encapsulated nanoparticles (3.3)	Li	LiTFSI 0.8 M LiNO_3_ 0.2 M DME:DOL (1:1 *v*/*v*)	~2.0; 1.5–2.8 ^g^	1500 ^f^ (0.1 C); -	~704.1 ^f^; 500; 0.5 C; 99%; -	1488.1–736.7 ^f^; 0.1–1 C	[433]
14	S@MnO_2_@ PPy; encapsulated spheres (0.5)	Li	LiTFSI 1 M DME:DOL (1:1 *v*/*v*)	-; 1.7–3.0 ^g^	1367 ^f^ (0.2 C); >95%	~800 ^f^; 500; 0.5 C; ~98%; -	1367–1050 ^f^; 0.2 C–0.5 C	[434]
15	AC@Fe3O4-NC@; coated fibers (4.7)	Li	LiTFSI 1 M DME:DOL (1:1 *v*/*v*)	2.1; 1.7–2.8 ^g^	~1316 ^f^; ~100%	780 ^f^; 1000; 0.2 C; 100%; -	~1316–531 ^f^; 0.1 C–4 C	[435]
17	S-Fe@PPy; coaxial tubes (4.0)	Li	LiTFSI 1 M en DME:DOL (1:1 *v*/*v*)	~2.0; 1.7–2.8 ^g^	1117 ^f^; -	525 ^f^; 200; 1 C; 90.9%; -	859–284 ^f^; 0.1 C–5 C	[436]
18	Na	Co_1−x_S/NC; nano-esferas (1–1.5)	CF_3_NaO_3_S 1 M en TGM	-; 0.01–3.0 ^k^	-	472; 120; 500 ^d^; -; -	513.5–220.4; 100–2000 ^d^	[436]
19	Na	Nano-hilos- CoP@PPy/CP (1.8)	NaClO_4_ 1 M en EC:DEC (1:1 *v*/*v*)	-; 0.01–2.5 ^k^	1318 ^h^; 69.06%	0.443 ^h^; 1000; 1.5 ^i^; ~99.5%; -	0.636–0.285 ^h^; 0.15–3 ^i^	[437]
20	SS@MnO_2_@ PPy	NT@Zn	Gel de ZnSO_4_ y MnSO_4_ en gelatina-bórax	-; 0.8–1.9 ^l^	-	60% *; 1000; 2 C; ~99%; -	174.2–60.0 ^f^; 0.5 C–4 C	[438]
21	MnO_x_/PPy (0.35)	Zn	ZnSO_4_ en gelatina-bórax	0.9; 0.6–1.5 ^l^	110 ^j;f^; -	21 mWh cm^−3^	110–38.5 ^j;f^; 0.2–1.0 ^i^	[439]

^a^ Mass charge in mg cm^−2^ unless otherwise specified/^b^ Specific capacity in mAh g^−1^ relative to the mass of active materials of the anode unless otherwise indicated/^c^ Current density expressed in A g ^−1^/^d^ Current density expressed in mA g^−1^/^e^ Reported in Wh kg^−1^, generally related to the capacity reported in the same column, except when referring to the maximum/^f^ Regarding the mass of cathode active materials/^g^ (vs. Li^+^/Li)/^h^ Capacity reported in mAh cm^−2^/^i^ Current density reported in mA cm^−2^/^j^ Capacity reported in µAh cm^−2^/^k^ (vs. Na^+^/Na)/^l^ (vs. Zn^2+^/Zn)/* Regarding initial discharge capacity/“-” is for unreported parameters.

**Table 8:** PEDOT and other nanostructured PTh derivatives in various configurations were evaluated for rechargeable battery electrodes, with their performance parameters.

#	Active Cathode Material; form (Mass Loading of Active Materials ^a^)	Active Anode Material; form (Mass Loading of Active Materials ^a^)	Electrolyte	Medium Operating Potential; Potential Window/V	Specific Initial Discharge Capacity ^b^; Coulombic Efficiency	Long-Term Performance: Reversible Capacity ^b^; Cycles; Speed or Current Density; Coulombic Efficiency; Energy Density	Speed Capability: Specific Capabilities; Velocities and Current Densities	Ref
01	Li	PEO-PEDOT:PSS/PEI/Si; coated nanoparticles (1.0)	LiPF_6_ 1 M EC:DEC (1:1 *p*/*p*)	-; 0.01–1.0 ^g^	2440; 82.0%	2027; 500; 1.0 ^c^; 100%; -	~3000–1500; 0.2–8.0 ^c^	[440]
02	Li	Si@PEDOT-VAA-D-Sorbitol; coated nanoparticles (1.0)	LiPF_6_ 1 M EC:DEC (1:1 *v*/*v*)	-; 0.01–1.0 ^g^	3788; 80%	1500; 700; 0.2 C; ~100%; -	2739–737; 0.1 C–2 C	[441]
03	Li	Spheres-Fe3O4@ PEG/CB/FWNT/ PPBT; linked networks (2.1–2.5)	LiPF_6_ 1.2 M EC:DEC (1:1 *v*/*v*)	-; 0.01–3.0 ^g^	~1000; 92%	880; 200; 0.5 C; ~99%; -	~900–100; 0.1 C–3.0 C	[442]
04	Li	NCA@P3HT-CNT; covered particles	LiPF_6_ 1 M EC:DMC (1:1 *v*/*v*)	-; 2.7–4.2 ^g^	156 (1 C); 85%	80% *; 1000; 16 C; -; -	156–83; 1 C–32 C	[443]
05	NCM811@poly(3-alkylthiophene); coated particles (10.0)	Li	LiPF_6_ 1 M EC:EMC (1:1 *v*/*v*)	-; 3.0–4.3 ^g^	~183.8 ^h^; -	174.4 ^h^; 50; 1 C; -; -	-	[444]
06	M-NVP@ PEDOT; coated particles (3.0)	Na	NaPF_6_ 0.5 M en PC	-; 4.5–2.2 ^l^	138.8 ^h^ (1 C); -	112.4 ^h^; 500; 1 C; -; -	143.3–109.8 ^h^; C/50–10 C	[445]
07	DC/V2O5@ PEDOT; nanosheets (2.0)	Zn	Zn(CH_3_O_3_F)_2_ 2.5 M	-; 0.2–1.6 ^i^	448.2 ^h^; 76.6%	223.6 ^h^; 1000; 5.0 ^c^; ~100%; 243.3	356.2–232.1 ^h^; 0.1–20.0 ^c^	[446]
08	OD-ZnMn2O4@PEDOT; coated fibers (6.0)	Zn	ZnSO_4_ 1 M	-; 0.8–1.9 ^i^	221 ^h^ (0.5 mA cm^−2^); -	93% *; 300; 8 ^j^; -; -	221–62.5 ^h^; 0.5–10 ^j^	[447]
09	OD-ZnMn2O4@PEDOT; coated fibers (6.547)	Zn	LiCl; ZnCl_2_; MnSO_4_ in PVA	~1.3; 0.8–1.9 ^i^	-	80.5% *; 300; 8 ^j^; ~100%; 273.4	207–140 ^h^; 0.5–10 ^j^	[447]
10	[EMIM]PF_6_- PEDOT:PSS/ Bi_2_S_3_	Zn	Zn(TSFI)_2_ 1 M + LiTFSI 21 M	1.4; 0.1–2.3 ^i^	137.5 ^h^ (2 ^c^); -	131 ^h^; 5300; 2 ^c^; 100%; -	275–105 ^h^; 0.3–6 ^c^	[448]
11	[EMIM]PF_6_- PEDOT:PSS/ Bi_2_S_3_	Zn	Zn(TSFI)_2_ y LiTFSI en PAM	1.4; 0.1–2.3 ^i^	192.4 ^h^ (1 ^c^); -	177 ^h^; 5300; 1 ^c^; 100%; 315 máx,	101–50 ^h^; 0.3–6 ^c^	[448]
12	S/PEDOT; 3D mesoporous (2.4)	C; 3D double mesoporous gyroid	Li^+^ conductive PPO polymer	2.5; 1.0–3.0 ^g^	0.9 ^k^ (0.125 ^j^); -	375 ^h^; 20; 30 ^d^; -; -	-	[449]

^a^ Mass charge in mg cm^−2^ unless otherwise specified/^b^ Specific capacity in mAh g^−1^ relative to the mass of active materials of the anode unless otherwise indicated/^c^ Current density expressed in A g^−1^/^d^ Current density expressed in mA g^−1^/^e^ Energy density in Wh kg^−1^/^f^ Regarding the total mass of the anode/^g^ (vs. Li/Li^+^)/^h^ Regarding the mass of active materials of the cathode/^i^ (vs. Zn^2+^/Zn)/^j^ Current density expressed in mA cm^−2^/^k^ Expressed in mAh cm^−2^/^l^ (vs. Na^+^/Na)/“-” is for parameters not reported/* Regarding initial capacity download.

**Table 9:** Other polymeric nanostructures are evaluated for rechargeable battery electrodes, with their performance parameters.

#	Active Cathode Material; form (Mass Loading of Active Materials ^a^)	Active Anode Material; form (Mass Loading of Active Materials ^a^)	Electrolyte	Medium Operating Potential; Potential Window/V	Specific Initial Discharge Capacity ^b^; Coulombic Efficiency	Long-Term Performance: Reversible Capacity ^b^; Cycles; Speed or Current Density; Coulombic Efficiency; Energy Density	Speed Capability: Specific Capabilities; Velocities and Current Densities	Ref
01	PPTO; nanoparticles (1.5–2.5)	Li	LiTFSI 1.0 M DOL/DME (1:1 *v*/*v*)	2.44; 1.5–3.5 ^f^	~180 ^h^; 77%	97 ^h^; 1000; 100 ^d^; 100%; 530	230–22 ^h^; 20–300 ^d^	[450]
02	PEPTO; nanoparticles (1.5–2.5)	Li	LiTFSI 1.0 M DOL/DME (1:1 *v*/*v*)	2.47; 1.5–3.5 ^f^	~185 ^h^; 76%	110 ^h^; 1000; 800 ^d^; 99.8%; 507	249–98 ^h^; 20–1500 ^d^	[448]
03	X-PVMPT; nanoparticles (0.41–0.74)	Li_4_Ti_5_O_12_	LiPF_6_ 1.0 M EC:DMC (1:1 *v*/*v*)	1.9; 1.3–2.3 ^f^	84 (1 C); ~95%	83.5; 100; 1 C; 100%; -	-	[451]
04	X-PVMPT; nanopartículas (0.41–0.74)	Li_4_Ti_5_O_12_	LiPF_6_ 1,0 M en EC:DMC (1:1 *v*/*v*)	1.9; 1.3–2.3 ^f^	84 (1 C); ~95%	83.5; 100; 1 C; 100%; -	-	[451]
05	LiFePO4-PFA; coated particles (1.56–5.19)	Li	LiPF_6_ 1.0 M EC:DEC (1:1 *v*/*v*)	3.3; 2.5–4.2 ^f^	~140 ^h^ (0.5 C); -	80% *; 500; 2 C; 99.9%; 1350.5 ^g^	160.3–103.4 ^h^; 0.1 C–5 C	[452]
06	LiFePO4-PFA; coated particles (1.56–5.19)	Graphite	LiPF_6_ 1.0 M EC:DEC (1:1 *v*/*v*)	3.3; 2.5–4.2 ^f^	70.5 ^h^; 98.1%	53.2 ^h^; 100; -; 99%; -	-	[452]
07	LiNi_0.6_Co_0.2_Mn_0.2_ O_2_@ polyphenylene; coated particles (3.0)	Li	LiPF_6_ 1.0 M EC:DEC (1:1 *v*/*v*)	-; 3.0–4.4 ^f^	148 ^h^ (0.1 C); -	113 ^h^; 1150; 0.5 C; 100%; -; (−20 °C)	128–43 ^h^; 0.5 C–2 C	[453]
08	Li	CMP-PBIM; nanosheets	LiPF_6_ 1.0 M EC:DEC (1:1 *p*/*p*)	-; 0.0–3.0 ^f^	1567; 55.07%	510; 1000; 1000 ^d^; ~99%; -	807–214; 0.05 C–2.5 C	[454]
09	Li	Si@C/derivative of P(iso); agglutinated nanoparticles	LiPF_6_ 1.0 M EC:DEC (1:1 *v*/*v*)	-; 0.01–1.0 ^f^	3507; 49%	~1450; 300; 0.2 C; 99.56%; -	~1400–100; 0.2 C–2 C	[455]
10	Fe3O4@NC- derived from PoPD; nanospheres (2.5)	Li	LiPF_6_ 1.0 M EC:DEC:DMC (1:1:1 *v*/*v*)	-; 0.01–3.0 ^f^	~1550 ^h^; 62.5%	918 ^h^; 200; 0.1 C; ~99%; -	902 ^h^-458; 0.1 C–2 C	[456]
11	COF-PIBN/ graphene; microporous (2.0)	Li	LiTFSI 1.0 M DOL/DME (1:1 *v*/*v*)	~2.3; 1.5–3.5 ^f^	242.3 ^h^ (1 C); -	182.3 ^h^; 300; 5 C; ~100%; 601	271–244,8 ^h^; 0.1 C–10 C	[457]
12	Py-COF/S; microporous (0.8–1.2)	Li	LiTFSI 1.0 M DOL:DME (1:1 *v*/*v*)	~2.0; 1.8–2.7 ^f^	1200 ^h^ (1.0 C); ~90%	418 ^h^; 500; 5.0 C; ~100%; -	1145–659 ^h^; 0.5 C–5.0 C	[458]
13	S-TAPB-DVA-COF(S-COF-V); Microporous (~0.7)	Li	LiTFSI 1.0 M DOL:DME (1:1 *v*/*v*)	~2.0; 1.7–2.8 ^f^	1400 ^h^; 94.5%	416 ^h^; 1000; 1 C; ~100%; -	1324–431 ^h^; 0.2 C–6 C	[459]
14	SPAN; nanoparticles (0.8)	K	KPF_6_ 0.5 M EC:DMC (1:1 *v*/*v*)	-; 0.01–3.0 ^j^	~1700 ^h^; -	1050 ^h^; 100; 0.5 C; ~99%	1050–550 ^h^ 0.5 C–3 C	[460]
15	Na	MOF-TFPB- TAPT; microporous microspheres (1.65)	NaPF_6_ 1.0 M EC:DMC (1:1 *v*/*v*)	0.65; 0.01–1.6 ^i^	770; 44%	125; 500; 30 ^d^; ~98%; 81.25	245–145; 30–200 ^d^	[461]
16	Na	cPAN; nanofibers (~1.3)	NaPF_6_ 1.0 M DME	-; 0.001–3.0 ^i^	547; 53.9%	196; 3500; 5 ^c^; 99.9%; -	527–200; 0.05–5 ^c^	[462]
17	Se-CPSe; nanosheets (~2.0)	Na	NaClO_4_ 1.0 M EC:PC (1:1 *v*/*v*)	~1.2; 0.5–3.0 ^i^	420 ^h^ (1000 ^d^); 71%	382 ^h^; 1000; 1000 ^d^; ~100%; -	570–325 ^h^; 100–3200 ^d^	[463]
18	K	CMP-PyBT; nanoparticles	KPF_6_ 0.6 M EC:DEC (1:1 *v*/*v*)	-; 0.1–3 ^j^	~1050; ~40%	272; 500; 50 ^d^; 99.5%; -	428–104; 30–500 ^d^	[464]

^a^ Mass charge in mg cm^−2^/^b^ Specific capacity in mAh g^−1^ with respect to the mass of active materials of the anode unless otherwise indicated/^c^ Current density expressed in A g^−1^/^d^ Current density expressed in mA g^−1^/^e^ Energy density in Wh kg^−1^/^f^ (vs. Li/Li^+^)/^g^ Expressed in Wh L^−1^/^h^ Referred to the mass of active materials of the cathode/^i^ (vs. Na^+^/Na)/^j^ (vs. K^+^/K)/“-” is for parameters not reported/* Regarding initial capacity download.

## Data Availability

Not applicable.
